# Bibliometric Analysis of Global Circular RNA Research Trends from 2007 to 2018

**DOI:** 10.22074/cellj.2021.7143

**Published:** 2021-05-26

**Authors:** Ran Wu, Fei Guo, Chen Wang, Baohua Qian, Fuming Shen, Fang Huang, Weidong Xu

**Affiliations:** 1. Department of Pharmacy, Shanghai Tenth People’s Hospital, Tongji University School of Medicine, Shanghai, China; 2. Department of Transfusion Medicine, Changhai Hospital, Second Military Medical University, Shanghai, China; 3. Department of Orthopaedics, Changhai Hospital, Second Military Medical University, Shanghai, China

**Keywords:** Bibliometric, Circular RNA, Citation, CiteSpace, VOSviewer

## Abstract

**Objective:**

Circular RNA (circRNA) is of significant interest in genetic research. The aim of this study was to assess global trends in circRNA research production in order to shed new light on future research frontiers.

**Materials and Methods:**

In this retrospective study, we conducted a literature search using the Web of Science Core Collection (WoSCC) database on March 21, 2019 to retrieve publications from 2007 to 2018. Excel 2013, CiteSpace V, and VOSviewer were used to evaluate bibliometric features that included publication output, countries/regions, institutions, journals, citation frequency, H-index, and research hotspots.

**Results:**

Global cumulative publication output on circRNA consisted of 998 papers with a total citation of 28 595 during 2007-2018. China, the US, and Germany were the most prolific countries. China ranked first in H-index (60 times) and citations (13 333 times). The most productive institution was Nanjing Medical University with 73 papers. Biochemical and Biophysical Research Communications (impact factor [IF]2017:2.559) ranked first among journals in the number of publications (64 papers). The keywords shifted from "sequence", "intron", and "splice-site" to "transcriptome", "microRNA sponge", "exon circularization", and "circRNA biogenesis" overtime. The burst keywords "transcriptome", "microRNA sponge", "exon circularization", and "circRNA biogenesis" were the latest frontiers by 2018.

**Conclusion:**

This is a relatively novel bibliometric analysis to inspect research related to circRNA. The results show that publications have continuously increased in the past decade. China, the US, and Germany were the leading countries/regions in terms of quantity. Recent studies on topics related to circRNA biogenesis and function should be closely followed in this field.

## Introduction

The concept of "circular RNA (circRNA)" was proposed by Sanger et al. (1) when they reported that viroids are pathogenic to certain higher plants with single-stranded covalently closed circRNA molecules. circRNAs mostly stem from either exons (2, 3) or introns (4, 5). The covalently closed loop is characterized by neither 5´-3´ polarity nor a polyadenylated tail (6), and this distinguishes circRNAs from linear RNAs. Meanwhile, circRNAs are more stable, even when treated with RNase R (7). Researchers initially believed that circRNAs were by-products in the aberrant splicing process, and had little role in biological processes (2). With the rapid advances of high-throughput RNA sequencing (RNA-seq) and bioinformatics, numerous endogenous, diverse, widespread and conserved circRNAs have been identified (8-10). Therefore, these molecules caused a resurgence in interest by researchers. Of particular note, some studies have shown that circRNAs could act as microRNA (miRNA) sponges and regulate line RNA transcription and protein production to modulate gene expression (11-13).

Recent evidences indicated that circRNA plays a role in aging (9, 14) and tissue development (15). circRNAs might be involved in neurological disorders (16), atherosclerotic vascular disease risk (17), Alzheimer’s disease (18), and cancer (19). Thus, they might be potentially valuable in disease diagnosis, prognosis, and precise therapy (20, 21). Simultaneously, database setups for circRNA in the last few years include circBase, CIRCpediav2, and CircInteractome (TableS1, See Supplementary Online Information at www.celljournal.org). These databases make it more convenient for researchers to access and study circRNA, and facilitates progress in this field.

Although researches related to circRNA have flourished in recent years, there have been limited attempts to systematically explore the development of scientific productivity in this area. To our knowledge, there are a few reports on research activity in circRNA that have been published internationally. The focus of bibliometrics is on literature systems and literature metrology characteristics; they statistically and mathematically analyse written publications such as books and periodicals (22). This is a reliable method to analyse literature in the field of science and characterize the tendency of research activity over time. Bibliometrics has contributed to research trends in cardiovascular diseases (23), gastrointestinal diseases (24), and diabetes (25).

The aims of present study were to systematically evaluate the international publication productivity of circRNA research using the Web of Science (WoS) from 2007 to 2018; analyse the most productive countries/ institutions/journals; and measure geographic and time distribution of literature that pertained to circRNA.

## Materials and Methods

### Patient and public involvement

In this retrospective study, no patient or public involvement was available.

### Sources of data and the search strategy

We searched literature in the online version of Science Citation Index-Expanded (SCIE), Web of Science Core Collection (WoSCC), and Essential Science Indicator (ESI) databases on March 21, 2019. We downloaded the data from a public database as secondary data, which did not involve ethical considerations. Thus, ethical approval was not applicable in this situation. 

We used the following search strategy: (TI=("circRNA*") OR TI=("circular RNA*") OR TI=("circRNA_*") OR TI=("circular noncoding RNA*") OR TI=("circular non coding RNA*") OR TI=("circular untranslated RNA*") OR TI=("circular non translated RNA*") OR TI=("circular non protein coding RNA*") OR TI=("circular ncRNA*")) AND publishing year=(2007- 2018) AND Language=(English). Refining for certain document types: the document types were selected as "article" or "review", and we only considered peer-reviewed documents. We chose 2007 as the start check point because it articles in this field began to emerge continuously in 2007.

### Data collection

WoSCC was used to analyse the characteristics of the publications, such as annual publications, countries/ regions, institutions, journal sources, citation frequency, impact factor (IF), weighted IF (IF^2^), H-index, etc. The H-index, citation frequency, IF, and IF^2 ^ were used to qualitatively measure the scientific research performance. IFs were obtained based on the Journal Citation Reports (JCR) 2017 and IF^2 ^ was calculated according to Rasim et al. (26).

The H-index, created by Hirsch (27) in 2005, can more perfectly reveal a country’s or individual’s achievement. This index takes both the quantity of published papers and the citation frequency into account, which means that H papers published by a researcher/institution/country received at least H citations. A higher H-index shows the larger influential power.

All data were gathered and verified by two authors independently (Ran Wu and Fei Guo). The data in "txt" form were downloaded from WoS and imported into Microsoft Excel 2013, CiteSpace V (64 bits), and VOSviewer (Version1.6.6, Leiden University, Leiden, The Netherlands). 

### Statistical analysis

A fitting mathematical model that used Microsoft Excel 2013 was employed to analyse the temporal tendency of the publications. The model: f(x)=ax^4^ +bx^3^ +cx^2^ +dx+e was applied to model the cumulative number of publications and present a prediction of the future tendency of circRNA outputs. The symbol x represented the year, and f(x) represented the annual number of publications by year.

The world map of publication distribution was generated by GunnMap 2 (http://www.lert.co.nz/map/). GraphPad Prism version 6.01 (San Diego, CA, USA) was employed to analyse Pearson’s correlation between publication number and gross domestic product (GDP) or the population number. P<0.05 were considered to be statistically significant. VOSviewer was used for the bibliometric analysis and visualization of the literature (28). In this study, it was used to analyse the collaboration between countries/regions and institutions. Network visualization of journals’ citation analysis was also derived through VOSviewer. CiteSpace V was used to construct a knowledge map of journals and keywords, and to obtain burst keywords that had the strongest citation.

### Results

#### Distribution of countries/regions according to circular RNA 

**Fig.1:**
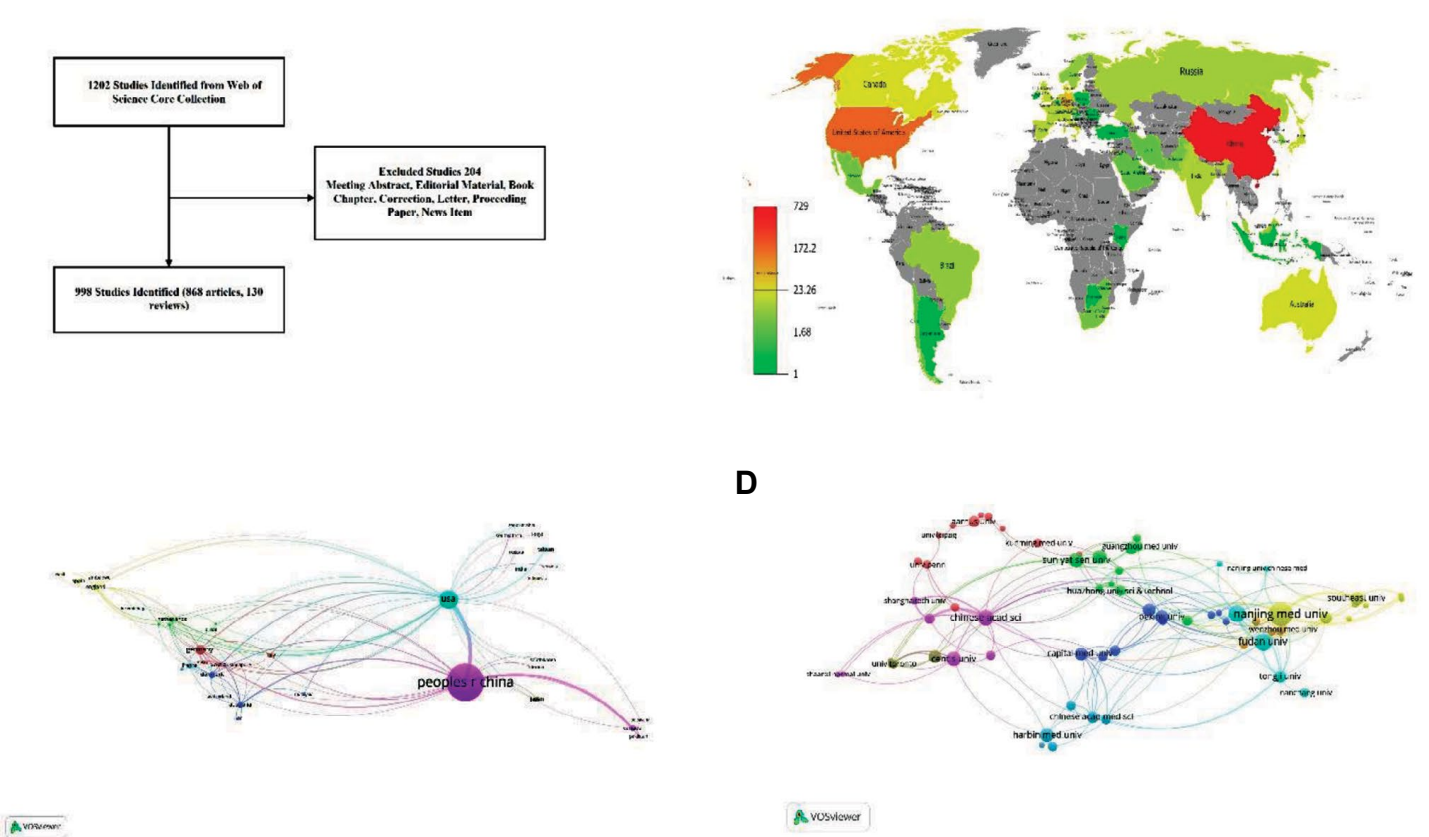
Publication distribution and collaboration analysis. **A.** Flowchart of included circular RNA (circRNA) research, **B. **Geographical distribution of publications related to circRNA research, **C. **Collaboration networks of countries/regions with at least one publication of circRNA research, and D. Collaboration networks of institutions with at least five publications of circRNA research.

A total of 998 studies fulfilled the search criteria (Fig .1A, Fig .S1, See Supplementary Online Information at www. celljournal.org), of which the majority were articles (868, 87.0%), followed by reviews (130, 13%). Figure 1B shows the geographical distribution of publications by individual countries/regions. There were a total of 46 countries/regions. Table 1 lists the top 10 most productive countries/regions; China, with 729 publications ranked first, followed by the US (181), Germany (45), Denmark (23), and Canada (21). After adjustments for GDP and population, we noted that Demark had the most publications per GDP (0.071) and the most publications per million people (3.986). There was an excellent correlation between publication numbers and population (r=0.996, P<0.0001) (Fig .S2A, See Supplementary Online Information at www.celljournal.org). No correlation was found between publication numbers and GDP (r=0.606, P=0.063) (Fig. S2B, See Supplementary Online Information at www. celljournal.org). The VOSviewer result showed extensive collaborations between countries/regions (Fig .1C). 

#### Distribution of institutions that published research related to circular RNA

A total of 919 institutions published researches related to circRNA (Table S2, See Supplementary Online Information at www.celljournal.org). The most productive institution was Nanjing Medical University, which published a total of 73 papers. The Chinese Academy of Sciences and Fudan University tied for second with 41 papers. Publications from the top 10 institutions accounted for 34.47% of all literature on circRNA. Figure 1D shows the collaborations between institutions with at least five publications.

**Table 1:** Top 10 most prolific countries in the field of circRNA research


Rank	Country	Number	Number per GDP* USD (billion)	Number per million population

1	China	729	0.060	0.526
2	USA	181	0.009	0.556
3	Germany	45	0.012	0.544
4	Denmark	23	0.071	3.986
5	Canada	21	0.013	0.572
6	Australia	20	0.015	0.813
7	England	14	0.005	0.212
8	Japan	14	0.003	0.110
9	Italy	13	0.007	0.215
10	France	12	0.005	0.179


*circRNA; Circular RNA and GDP; Gross domestic product.

### Publication outputs and growth prediction

**Fig.2:**
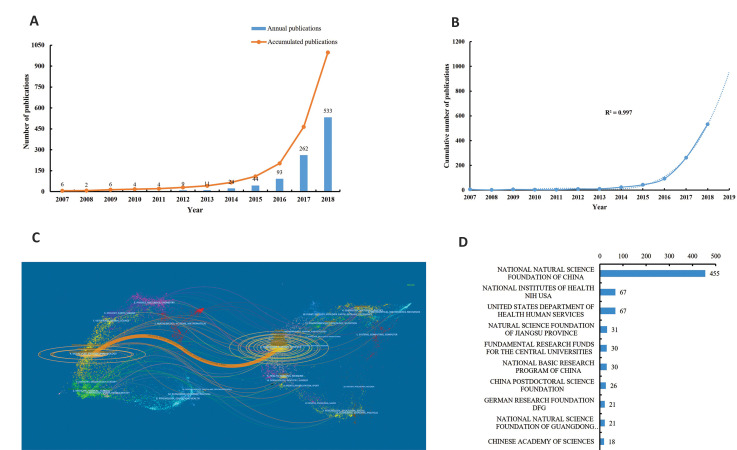
Publication output and growth prediction. **A.** Annual and accumulated publications of circular RNA (circRNA) research from 2007 to 2018, **B.** The model fitting curve of circRNA publication growth, **C.** Dual-map overlay of journals. There was one main citation path coloured with orange. Publications about circRNA research in molecular, biology, and immunology journals mainly cited journals in the molecular, biology, and genetics areas, and **D.** The top 10 funding agencies that supported circRNA research.

The annual publication numbers and accumulated publications are presented in Figure 2A. The annual publications were stably low from 2007 to 2013, and remarkable growth was observed since 2014. In total, the publications related to circRNA consistently increased during the last decade.

As shown in Figure 2B, there was a significant correlation between the publication year and annual number of circRNA publications (R^2^ =0.997). Worldwide, this was estimated to reach 955 publications in 2019.

### Distribution of published journals and funding agencies that focused on circular RNA 

The 998 publications on circRNA research appeared in 331 journals (Table S3, See Supplementary Online Information at www.celljournal.org). Table 2 shows the top 20 prolific journals. The Biochemical and Biophysical Research Communications journal (IF 2017: 2.559, IF^2^ 2017: 7.321) published the most literature related to circRNA research (64 articles, 6.413%), followed by Cellular Physiology and Biochemistry (IF 2017: NA, IF^2^ 2017: 14.373, 37 articles, 3.707%), Oncotarget (IF 2017: NA, IF^2 ^ 2017: 5.503, 36 articles, 3.607%), and Scientific Reports (IF 2017: 4.122, IF^2 ^ 2017: 3.706, 35 articles, 3.507%). There was one review in Nature Review Genetics (IF 2017: 41.465), which had the highest IF among the 331 journals. Among the top 20 prolific journals, Molecular Cancer had the highest IF^2^ (93.945). 

Figure 2C presents the dual-map overlay for the journals. The citing journal map is shown on the left and the cited journal map is displayed on the right. The disciplines covered by journals are marked in the label. Citation links that start from the journals on the left and end with those on the right are presented with lines. The map shows one main citation path, which indicates that most publications appeared in molecular, biology, and immunology journals. These publications were mostly cited from the molecular, biology, and genetics fields.

**Table 2:** Top 20 journals with most publications related to circRNA research


Rank	Journal	Count	Percent	IF 2017	IF^2^ 2017

1	Biochemical and Biophysical Research Communications	64	6.413	2.559	7.321
2	Cellular Physiology and Biochemistry	37	3.707	5.5	14.373
3	Oncotarget	36	3.607	NA*	5.503
4	Scientific Reports	35	3.507	4.122	3.706
5	Advances in Experimental Medicine and Biology	28	2.806	1.76	6.024
6	Circular RNAs Biogenesis and Functions	27	2.705	NA*	NA*
7	RNA Biology	23	2.305	5.216	55.590
8	PLOS One	20	2.004	2.766	1.655
9	Nucleic Acids Research	16	1.603	11.561	62.190
10	Biomedicine Pharmacotherapy	15	1.503	3.457	13.020
11	Molecular Cancer	14	1.403	7.776	93.945
12	Cancer Letters	13	1.303	6.491	32.335
13	Gene	13	1.303	2.498	13.970
14	International Journal of Clinical and Experimental Pathology	12	1.202	1.396	4.509
15	BMC Genomics	11	1.102	3.73	17.757
16	Epigenomics	11	1.102	4.979	22.944
17	European Review for Medical and Pharmacological Sciences	11	1.102	2.387	4.569
18	Oncology Letters	11	1.102	1.664	5.372
19	Aging US	10	1.002	5.179	36.308
20	Molecular Therapy Nucleic Acids	10	1.002	5.66	42.156


*circRNA; Circular RNA, NA; Not available, IF; Impact factor, and IF^2^ ; Weighted impact factor.

Totally, 998 publications on circRNA research were funded by 1241 funding agencies (Table S4, See Supplementary Online Information at www.celljournal. org). The National Natural Science Foundation of China supported 455 publications, which accounted for nearly half of all the literature in this case (45.5%). The top 10 funding agencies that supported circRNA research are presented in Figure 2D.

### Citation and H-index analysis

**Fig.3:**
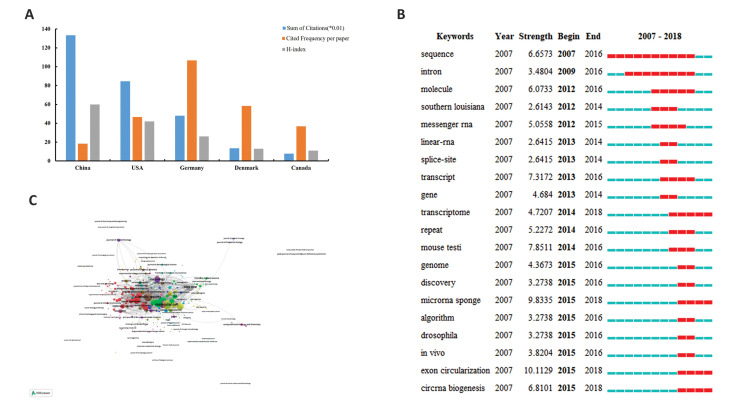
Quality analysis of countries/regions and journals. **A.** The distribution of citation (x0.01), cited frequency per paper, and H-index in the top five countries/regions, **B.** Network visualization of journal citation analysis. The larger spot indicates a higher citation frequency, and **C. **Top 20 keywords with the strongest citation bursts on circular RNA (circRNA) research published during 2007 and 2018.

Based on our analysis, the citation frequency number of all articles associated with circRNA was 28 595 by 2018. In terms of citations, China ranked first with 13 333 citations, followed by the US with 8460, Germany with 4798, Israel with 1615, and Denmark with 1344. The citation frequency per paper was 28.65 times, and Argentina had the highest frequency per paper (385), followed by Israel (179.44) and Germany (106.62) (Table S5, See Supplementary Online Information at www.celljournal.org). Figure 3A shows the citations and H-index results of the top five productive countries/regions. China, with an H-index value of 60, ranked first.

Citations analysis was conducted within all 331 journals. Our results demonstrated that Molecular Cell had the highest citation frequency (1908), followed by Nature (1519), and Scientific Reports (1464) (Fig .3B).

### Hotspots of studies on circular RNA

The total citations of the top 10 most cited publications varied from 386 to 1519 (Table 3). The IF numbers of the listed papers ranged from 2.766 to 41.577. The article that achieved the most citations (1519 times) was published by Memczak et al. (8).

Keywords used in the 998 papers were analysed with CiteSpace V. Totally, we extracted 202 keywords with 648 links, which were defined as the top 50 of the most frequent items from each year with the title, abstract, and keywords field under the condition of the CiteSpace V default setting (Fig. S3, See Supplementary Online Information at www. celljournal.org). The top 20 keywords with strongest citation bursts are shown in Figure 3C. According to the timeline, keywords shifted from "sequence", "intron", and "splice-site" to "transcriptome", "microRNA sponge", "exon circularization", and "circRNA biogenesis. The strongest ones included "exon circularization", "microRNA sponge", "mouse testi", "transcript", and "circRNA biogenesis".

**Table 3:** Top 10 studies with the most citation frequencies related to circular RNA research


Title	Journal	First author	Year	Cited by

Circular RNAs are a Large Class of Animal RNAs with Regulatory Potency (8)	Nature	Memczak, Sebastian	2013	1519
Circular RNAs are Abundant, Conserved, and Associated with ALU Repeats (29)	RNA-A Publication of the RNA Society	Jeck, William R.	2013	817
Circular RNAs are the Predominant Transcript Isoform from Hundreds of Human Genes in Diverse Cell Types (12)	PLOS ONE	Salzman, Julia	2012	587
circRNA Biogenesis Competes with Pre-mRNA Splicing (30)	Molecular Cell	Ashwal-Fluss, Reut	2014	522
Detecting and Characterizing Circular RNAs (31)	Nature Biotechnology	Jeck, William R.	2014	484
Exon-intron Circular RNAs Regulate Transcription in the Nucleus (32)	Nature Structural& Molecular Biology	Li, Zhaoyong	2015	465
Circular Intronic Long Noncoding RNAs (33)	Molecular Cell	Zhang, Yang	2013	457
Cell-Type Specific Features of Circular RNA Expression (34)	PLOS Genetics	Salzman, Julia	2013	430
Expanded Identification and Characterization of Mammalian Circular RNAs (35)	Genome Biology	Guo, Junjie U.	2014	396
Expression of Linear and Novel Circular Forms of an INK4/ARF-Associated Noncoding RNA Correlates with Atherosclerosis Risk (36)	PLOS Genetics	Burd, Christin E.	2010	386


## Discussion

Researchers previously focused on RNA with protein coding functions derived from DNA. In-depth studies and advanced technology make it clear that there are abundant and widespread noncoding RNAs (ncRNAs), which include miRNA, lncRNA, and circRNA. These RNAs could play significant roles in the life process (11, 21, 22). circRNA is an ncRNA, which was believed to be a by-product and have little function (2, 3). However, recent advances have implied that circRNA might participate in both physiological and pathological processes (9, 14- 19). This study aimed to quantitatively and qualitatively evaluate the bibliometric characteristics of circRNA research, and to inspect the future research frontier. Publications, to some extent, could be considered a judgment of development within a certain research field.

Researches related to circRNA have rapidly developed. To the best of our knowledge, this bibliometric analysis is the first attempt in this field. According to the results, the publication year can be separated into two stages. The first stage (2007-2013) had a slow increase in publications and was the initial phase of circRNA research. The second stage (2014-2018) had a sharp growth trend and was the flourishing phase of cicrRNA research. The number of publications in last few years exceeded the accumulative numbers in the early stage. With rapid and substantial progress in this field, the whole world was expected to maintain publishing papers about circRNA in a productive way. According to the prediction curve, more literature will be published in the circRNA research field in the future.

China, the US, and Germany were the leading countries in quantity (total publication number). After standardizing for GDP and population, Denmark ranked first with 0.071 publications per GDP and 3.986 publications per million people. Although Demark ranked fourth with 23 publications, we believed that a highly developed economy and smaller population compared to China and the US placed Denmark first after standardization. GDP and population are relevant to the publication output (37). In the present study, we found no correlation between publication numbers and GDP; however, the population number showed a positive correlation with publication numbers. We employed citations, cited frequency per paper, and H-index to analyse the quality. Among the top five prolific countries/regions, China, with an absolute advantage in publication numbers, scored the highest in both citations and H-index. However, Germany received the largest number of cited frequencies per paper. In terms of collaboration network, far-ranging cooperations were identified worldwide. The strongest cooperation was found between China and the US. Meanwhile, China and the US also had extensive cooperation with other countries/regions, respectively. Generally speaking, international cooperation is a result of cooperation between institutions worldwide (38). However, we found that Chinese institutions tend to collaborate nationally. This may partly explain the large output by China.

Chinese institutions preceded the quantity on circRNA research. The most productive worldwide was Nanjing Medical University. We mentioned that national collaborations were widespread in China. There were over 10 links between the prolific institutions (e.g., Nanjing Medical University, Fudan University, and Shanghai Jiao Tong University) and other institutions. Cooperation facilitates the progress of circRNA research from this perspective. Another interesting finding was that the majority of funding agencies were from China in this field. If one researcher in China successfully applied for major funding, such as the National Natural Science Foundation, and published high-quality articles, he or she might have priority to receive more funding, which becomes a cycle. This could also explain the productivity in China.

In terms of the top 20 prolific journals, Biochemical and Biophysical Research Communications, Cellular Physiology and Biochemistry, Oncotarget, and Scientific Reports were the main journals with over 30 publications. The first one was quantitative (64 publications) but not very qualitative (IF 2017: 2.559). The third one was removed from SCIE in 2018, although there were 36 publications. There were 16 papers in Nucleic Acids Research, of which the IF (11.561) was the highest in the top 20 prolific journal list. IF^2^ is a novel and more accurate indicator that assesses journal impact, which considers both the quantity of citations and the quality of cited journals (26). Molecular Cancer, with 14 papers, had the highest IF^2^ (93.945) among the top 20 productive journals in circRNA research. In general, future developments that pertain to circRNA would be likely showcased within the top 20 journals.

This study ranked the top 10 cited publications related to circRNA research. The evaluation presented informative insight into the development of popular opinion in the field of circRNA. The number of citations in circRNA varied from 386 to 1519. Undoubtedly, Memczak et al. (8) had a fundamental influence in the circRNA literature. The most influential article, titled "Circular RNAs are a Large Class of Animal RNAs with Regulatory Potency", was published in Nature in 2013 and was cited at least 1519 times. Memcazk et al. (8) provided evidence regarding the regulatory potential of circRNA. The second most frequently cited article by Jeck et al. (29) was published in RNA in 2013. This study reported the involvement of circRNA in control of gene expression.

Keywords assigned in each article or review can make delineation of the topics involved in circRNA research. Burst keywords, which were captured by CiteSpace V in this study, could make a reasonable prediction of research frontiers over time (39). The blue and red lines indicated time intervals and periods of citation bursts, respectively. With advanced technology, the research fields of circRNA transferred from discovery to in-depth mechanism and function, which was in line with the objective law of research. Below are the top four research frontiers of circRNA research:

i. Transcriptome: To date, circRNA that had been derived from pre-mRNA was primarily identified through high-throughput RNA-seq. It was not until the advanced RNA-seq detecting non-polyadenylated transcriptomes emerged that circRNA was found to be diverse and widespread (8, 10, 12, 29). Thus, transcriptome analysis was of great significance for circRNA identification and research.

ii. miRNA sponge: miRNAs are regulatory RNAs derived from hairpin transcripts. The results of recent studies show that some circRNAs might regulate gene expression at multiple levels (6). Of note, the primary finding was that circRNA could function as a miRNA sponge in the cytoplasm. circRNA competed with mRNA for miRNA biding and then regulated gene expressions (29).

iii. Exon circularization and iv. circRNA biogenesis: The biogenesis of circRNA has been uncovered after in-depth study. For instance, circRNAs are transcribed by RNA polymerase II (30, 40), and this biogenesis is regulated by the cis-regulatory elements and trans-acting factors that control splicing (6). Exon circularization is one of the necessary procedures of circRNA formation.

Although this is the first bibliometric study to comprehensively and objectively estimate global trends in circRNA research, there are some limitations. First, the total number of publications differs among the major databases - PubMed, Scopus, and Google Scholar. The use of the WoSCC database could have overlooked relevant publications from analysis. Second, the publications included in this analysis were restricted to the English language. Therefore, non-English papers, which are important, were excluded from the present study. Last but not least, all the searches were conducted over one day (March 21, 2019) to avoid bias; however, the database is constantly updating. Some high-quality publications are still being cited and this information may be omitted. Despite the aforementioned limitations, we believe that the overall results may not have changed.

## Conclusion

This study firstly provides a bibliometric analysis on global trends of circRNA research during 2007- 2018. Researches in this field have notably increased in recent years and will continue to emerge. Most studies associated with circRNA arose from China, the US, and Germany. China was the leading country with the highest H-index and citations. International cooperation was widely found throughout the world. The most prolific institution, Nanjing Medical University, was from China. Biochemical and Biophysical Research Communications had the most circRNA publications. "Transcriptome", "microRNA sponge", "exon circularization", and "circRNA biogenesis" might be the latest research frontiers that relate to the future for circRNA research.

## Supplementary PDF


