# Supplementary material for: Bibliometric Analysis of Global Circular RNA Research Trends from 2007 to 2018
Source: Cell J. 2021 May 26;23(2):238–46. doi: 10.22074/cellj.2021.7143 (PMC8181316; doi:10.22074/cellj.2021.7143)
Supplement: Supplementary file 1 [file Cell-J-23-238-s01.pdf]

www.celljournal.org

Supplementary Information for

Bibliometric Analysis of Global Circular RNA Research Trends from 2007 to 2018

Ran Wu, M.D.[1#], Fei Guo, B.M.[2#], Chen Wang, Ph.D.[3#], Baohua Qian, Ph.D.[2], Fuming Shen, Ph.D.[1],

Fang Huang, M.D.[1*], Weidong Xu, Ph.D.[3*]

1. Department of Pharmacy, Shanghai Tenth People's Hospital, Tongji University School of Medicine, Shanghai, China
2. Department of Transfusion Medicine, Changhai Hospital, Second Military Medical University, Shanghai, China
3. Department of Orthopaedics, Changhai Hospital, Second Military Medical University, Shanghai, China

# The first three authors contributed equally to this work.

**Corresponding Addresses: Department of Pharmacy, Shanghai Tenth People's Hospital, Tongji University School of Medicine, Shanghai, China*
Department of Orthopaedics, Changhai Hospital, Second Military Medical University, Shanghai, China
Emails: hazel_huang@126.com, xuwdshanghaichyy@163.com

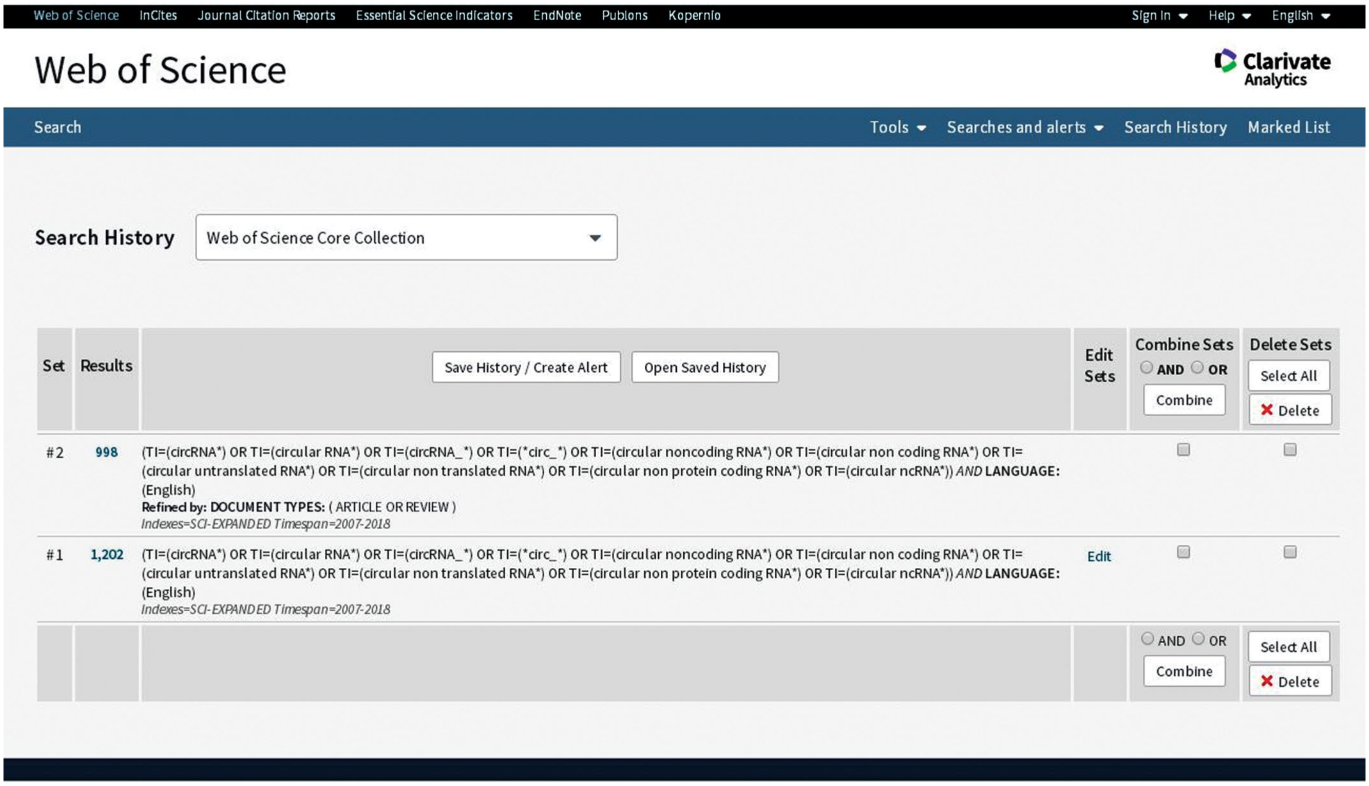

Fig.S1: Search history of circular RNA (circRNA) research.

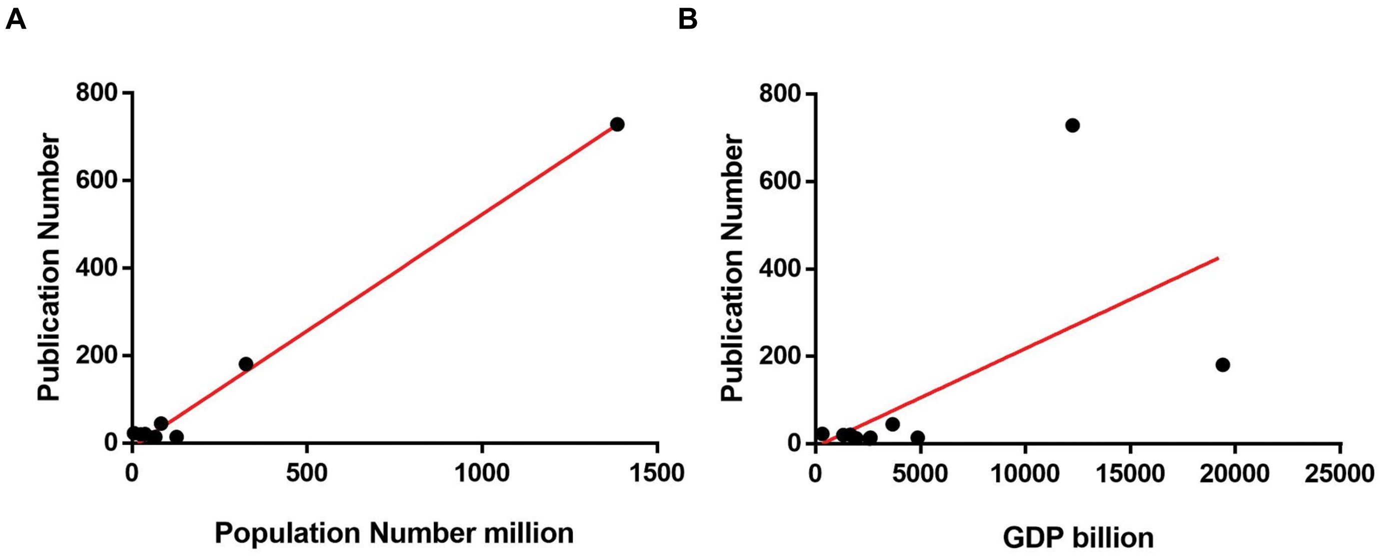

Fig.S2: Correlations between the publication numbers of circular RNA (circRNA) research and: **A.** the population number (r=0.996, P<0.001) and **B.** GDP (r=0.606, P=0.063). GDP; Gross domestic product.

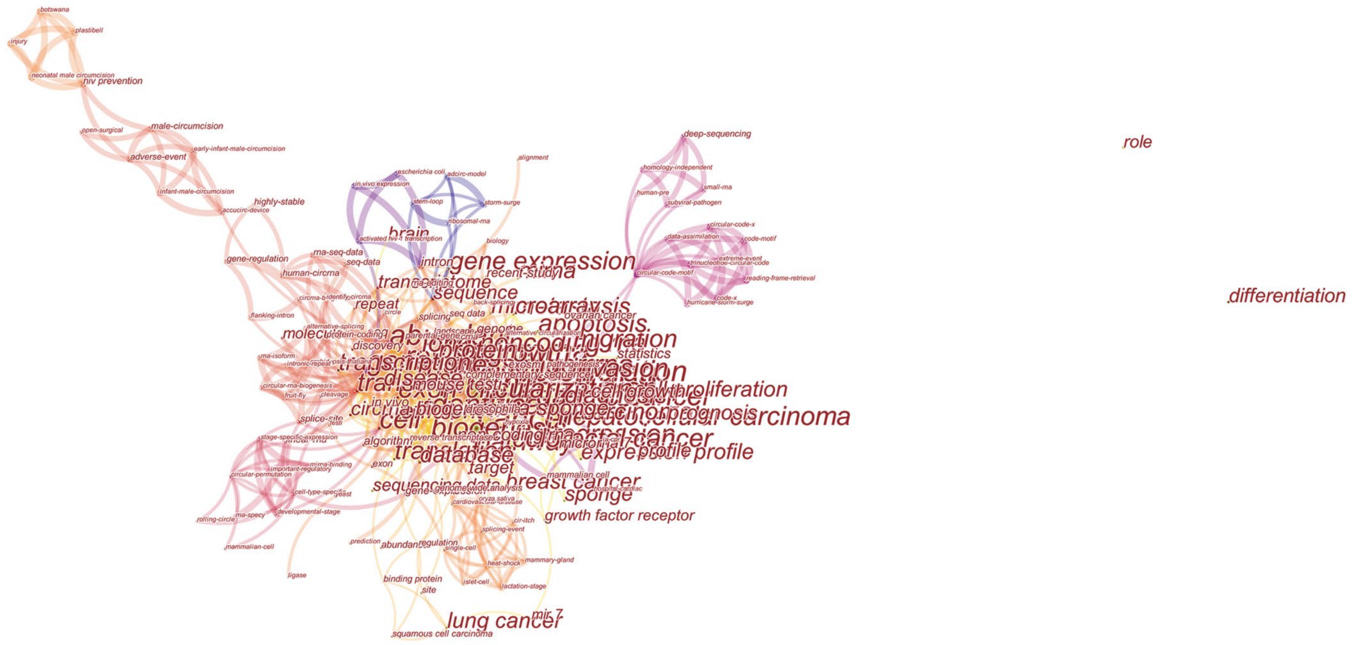

Fig.S3: Keyword network based on articles related to circular RNA (circRNA) research from 2007 to 2018.

Table S1: Databases set up for circRNA

Name	Website	Authors (Reference)	Journal	Year	PMID
circBase	http://circrna.org/	Glazar P et al. (1)	RNA	2014	25234927
CIRCpediav2	http://www.picb.ac.cn/rnomics/circpedia/	Dong et al. (2)	Genom Proteom Bioinf	2018	30172046
CircInteractome	http://circinteractome.nia.nih.gov	Dudekula et al. (3)	RNA Biol	2016	26669964
circRNABase	http://starbase.sysu.edu.cn/starbase2/mirCircRNA.php	Li et al. (4)	Nucleic Acids Res	2014	24297251
Circ2Traits	http://gyanxet-beta.com/circdb/	Ghosal et al. (5)	Front Genet	2013	24339831
CSCD (Cancer Specific CircRNA Database)	http://gb.whu.edu.cn/CSCD	Xia et al. (6)	Nucleic Acids Res	2018	29036403
TSCD (Tissue Specific CircRNA Database)	http://gb.whu.edu.cn/TSCD/	Xia et al. (7)	Nucleic Acids Res	2017	27543790
circNet	http://circnet.mbc.nctu.edu.tw/	Liu et al. (8)	Nucleic Acids Res	2016	26450965
deepBase v2.0	http://deepbase.sysu.edu.cn/	Zheng et al. (9)	Nucleic Acids Res	2016	26590255
circRNADb	http://202.195.183.4:8000/circrnadb/circRNADb.php	Chen et al. (10)	Sci Rep	2016	27725737
MiOncoCirc	http://mioncocirc.github.io/	Vo et al. (11)	Cell	2019	30735636
exoRBase	http://www.exoRBase.org	Li et al. (12)	Nucleic Acids Res	2018	30053265
circRNADisease	http://cgga.org.cn:9091/circRNADisease	Zhao et al. (13)	Cell Death Dis	2018	29700306
Circ2Disease	http://bioinformatics.zju.edu.cn/Circ2Disease/	Yao et al. (14)	Sci Rep	2018	30030469
PlantcircBase	http://ibi.zju.edu.cn/plantcircbase/	Chu et al. (15)	Mol Plant	2017	28315753
PlantcircNet	http://bis.zju.edu.cn/plantcircnet/	Zhang et al. (16)	Database-Oxford	2017	31725858

Table S2: All Institutions that published research related circRNA

**Institutions**	**Record count**	**% of 998**
NANJING MEDICAL UNIVERSITY	73	7.315
CHINESE ACADEMY OF SCIENCES	41	4.108
FUDAN UNIVERSITY	41	4.108
SHANGHAI JIAO TONG UNIVERSITY	37	3.707
SUN YAT SEN UNIVERSITY	29	2.906
CENTRAL SOUTH UNIVERSITY	28	2.806
HARBIN MEDICAL UNIVERSITY	24	2.405
NINGBO UNIVERSITY	24	2.405
PEKING UNIVERSITY	24	2.405
SOUTHEAST UNIVERSITY CHINA	23	2.305
CAPITAL MEDICAL UNIVERSITY	21	2.104
JILIN UNIVERSITY	21	2.104
MAX PLANCK SOCIETY	21	2.104
SOUTHERN MEDICAL UNIVERSITY CHINA	21	2.104
ZHEJIANG UNIVERSITY	21	2.104
TONGJI UNIVERSITY	20	2.004
UNIVERSITY OF TEXAS SYSTEM	20	2.004
CHINESE ACADEMY OF MEDICAL SCIENCES PEKING UNION MEDICAL COLLEG	19	1.904
HELMHOLTZ ASSOCIATION	19	1.904
SHANDONG UNIVERSITY	19	1.904
AARHUS UNIVERSITY	17	1.703
HARVARD UNIVERSITY	16	1.603
ANHUI MEDICAL UNIVERSITY	15	1.503
HUAZHONG UNIVERSITY OF SCIENCE TECHNOLOGY	15	1.503
SHANGHAI INSTITUTES FOR BIOLOGICAL SCIENCES CAS	15	1.503
UNIVERSITY OF CHINESE ACADEMY OF SCIENCES CAS	15	1.503
MAX DELBRUCK CENTER FOR MOLECULAR MEDICINE	14	1.403
SUZHOU UNIVERSITY	14	1.403
CHINA MEDICAL UNIVERSITY	13	1.303
QINGDAO UNIVERSITY	13	1.303
UNIVERSITY OF NORTH CAROLINA	13	1.303
CHINESE ACADEMY OF AGRICULTURAL SCIENCES	12	1.202
NANCHANG UNIVERSITY	12	1.202
SECOND MILITARY MEDICAL UNIVERSITY	12	1.202

Table S2: Continued

Institutions	Record count	% of 998
UNIVERSITY OF NORTH CAROLINA CHAPEL HILL	12	1.202
GUANGZHOU MEDICAL UNIVERSITY	11	1.102
SICHUAN UNIVERSITY	11	1.102
UNIVERSITY OF CALIFORNIA SYSTEM	11	1.102
UNIVERSITY OF TORONTO	11	1.102
KUNMING MEDICAL UNIVERSITY	10	1.002
STANFORD UNIVERSITY	10	1.002
SUNNYBROOK HEALTH SCIENCE CENTER	10	1.002
SUNNYBROOK RESEARCH INSTITUTE	10	1.002
UNIVERSITY OF PENNSYLVANIA	10	1.002
WENZHOU MEDICAL UNIVERSITY	10	1.002
ZHENGZHOU UNIVERSITY	10	1.002
CENTRE NATIONAL DE LA RECHERCHE SCIENTIFIQUE CNRS	9	0.902
CONSEJO SUPERIOR DE INVESTIGACIONES CIENTIFICAS CSIC	9	0.902
NANJING UNIVERSITY	9	0.902
NANTONG UNIVERSITY	9	0.902
SHANGHAITECH UNIVERSITY	9	0.902
XI AN JIAOTONG UNIVERSITY	9	0.902
HOWARD HUGHES MEDICAL INSTITUTE	8	0.802
JINAN UNIVERSITY	8	0.802
NATIONAL INSTITUTES OF HEALTH NIH USA	8	0.802
PEKING UNION MEDICAL COLLEGE HOSPITAL	8	0.802
TIANJIN MEDICAL UNIVERSITY	8	0.802
UTMD ANDERSON CANCER CENTER	8	0.802
ARMY MEDICAL UNIVERSITY	7	0.701
GERMAN CENTRE FOR CARDIOVASCULAR RESEARCH	7	0.701
MASSACHUSETTS GENERAL HOSPITAL	7	0.701
NEVADA SYSTEM OF HIGHER EDUCATION NSHE	7	0.701
NORTHWEST A F UNIVERSITY CHINA	7	0.701
RUSSIAN ACADEMY OF SCIENCES	7	0.701
SHANGHAI UNIVERSITY	7	0.701
UNIVERSITY OF NEVADA RENO	7	0.701
VA BOSTON HEALTHCARE SYSTEM	7	0.701
WUHAN UNIVERSITY	7	0.701

Table S2: Continued

Institutions	Record count	% of 998
YANGZHOU UNIVERSITY	7	0.701
HEBREW UNIVERSITY OF JERUSALEM	6	0.601
NANJING UNIVERSITY OF CHINESE MEDICINE	6	0.601
NIH NATIONAL INSTITUTE ON AGING NIA	6	0.601
PENNSYLVANIA COMMONWEALTH SYSTEM OF HIGHER EDUCATION PCSHE	6	0.601
RUPRECHT KARLS UNIVERSITY HEIDELBERG	6	0.601
STANFORD CANCER INSTITUTE	6	0.601
UNIVERSITY OF COPENHAGEN	6	0.601
UNIVERSITY OF LEIPZIG	6	0.601
UNIVERSITY OF TEXAS HEALTH SCIENCE CENTER HOUSTON	6	0.601
XUZHOU MEDICAL COLLEGE	6	0.601
BAYLOR COLLEGE OF MEDICINE	5	0.501
BENGBU MEDICAL COLLEGE	5	0.501
CHINESE PEOPLE S LIBERATION ARMY GENERAL HOSPITAL	5	0.501
CHONGQING MEDICAL UNIVERSITY	5	0.501
CONSIGLIO NAZIONALE DELLE RICERCHE CNR	5	0.501
CSIC INSTITUTE OF MOLECULAR CELLULAR BIOLOGY OF PLANTS IBMCP	5	0.501
FOURTH MILITARY MEDICAL UNIVERSITY	5	0.501
FREE UNIVERSITY OF BERLIN	5	0.501
GUANGDONG INST MICROBIOL	5	0.501
GUANGDONG MEDICAL UNIVERSITY	5	0.501
HUAZHONG AGRICULTURAL UNIVERSITY	5	0.501
NANJING MED UNIV	5	0.501
QIQIHAR MEDICAL UNIVERSITY	5	0.501
SHAANXI NORMAL UNIVERSITY	5	0.501
UNIVERSITY OF AMSTERDAM	5	0.501
UNIVERSITY OF SASKATCHEWAN	5	0.501
UNIVERSITY OF SCIENCE TECHNOLOGY OF CHINA	5	0.501
XIAMEN UNIVERSITY	5	0.501
ACADEMIC MEDICAL CENTER AMSTERDAM	4	0.401
CHARITE MEDICAL UNIVERSITY OF BERLIN	4	0.401
CLEVELAND CLINIC FOUNDATION	4	0.401
COMMUNAUTE UNIVERSITE GRENOBLE ALPES	4	0.401
CORNELL UNIVERSITY	4	0.401

Table S2: Continued

Institutions	Record count	% of 998
HUMBOLDT UNIVERSITY OF BERLIN	4	0.401
IMPERIAL COLLEGE LONDON	4	0.401
JIANGXI CHEST HOSP	4	0.401
LOUISIANA STATE UNIVERSITY	4	0.401
LOUISIANA STATE UNIVERSITY SYSTEM	4	0.401
MINIST EDUC	4	0.401
NANJING AGRICULTURAL UNIVERSITY	4	0.401
NANJING UNIVERSITY OF AERONAUTICS ASTRONAUTICS	4	0.401
NANKAI UNIVERSITY	4	0.401
NATIONAL UNIVERSITY OF SINGAPORE	4	0.401
PENN STATE UNIVERSITY	4	0.401
PIROGOV RUSSIAN NATIONAL RESEARCH MEDICAL UNIVERSITY	4	0.401
SHANDONG ACADEMY OF MEDICAL SCIENCES	4	0.401
SHANDONG AGRICULTURAL UNIVERSITY	4	0.401
SHANDONG PROVINCIAL HOSPITAL	4	0.401
SHANGHAI KEY LAB VISUAL IMPAIRMENT RESTORAT	4	0.401
SOUTH CHINA AGRICULTURAL UNIVERSITY	4	0.401
STATE KEY LAB ONCOLOGY SOUTH CHINA	4	0.401
TECHNICAL UNIVERSITY OF MUNICH	4	0.401
UNIVERSITAT POLITECNICA DE VALENCIA	4	0.401
UNIVERSITE DE STRASBOURG	4	0.401
UNIVERSITE GRENOBLE ALPES UGA	4	0.401
UNIVERSITES DE STRASBOURG ETABLISSEMENTS ASSOCIES	4	0.401
UNIVERSITI MALAYA	4	0.401
UNIVERSITY OF COLORADO SYSTEM	4	0.401
UNIVERSITY OF JINAN	4	0.401
UNIVERSITY OF MUNICH	4	0.401
UNIVERSITY OF NEW SOUTH WALES SYDNEY	4	0.401
UNIVERSITY OF TEXAS AUSTIN	4	0.401
ACADEMIA SINICA TAIWAN	3	0.301
ANHUI PROV HOSP	3	0.301
ANHUI PROV KEY LAB MAJOR AUTOIMMUNE DIS	3	0.301
BEIJING GENOMICS INSTITUTE BGI	3	0.301
CHINA AGRICULTURAL UNIVERSITY	3	0.301

Table S2: Continued

Institutions	Record count	% of 998
COUNCIL OF SCIENTIFIC INDUSTRIAL RESEARCH CSIR INDIA	3	0.301
DALIAN MEDICAL UNIVERSITY	3	0.301
FU WAI HOSPITAL CAMS	3	0.301
FUJIAN MEDICAL UNIVERSITY	3	0.301
GOETHE UNIVERSITY FRANKFURT	3	0.301
GUANGDONG ACADEMY OF MEDICAL SCIENCES	3	0.301
GUANGDONG GENERAL HOSPITAL	3	0.301
GUIZHOU MEDICAL UNIVERSITY	3	0.301
HANGZHOU MEDICAL COLLEGE	3	0.301
HEBEI MEDICAL UNIVERSITY	3	0.301
HELMHOLTZ CENTER MUNICH GERMAN RESEARCH CENTER FOR ENVIRONMENTA	3	0.301
HENAN UNIVERSITY	3	0.301
HOKKAIDO UNIVERSITY	3	0.301
INSTITUTE OF CROP SCIENCES ICS	3	0.301
INSTITUTE OF MEDICAL BIOLOGY CAMS	3	0.301
JAPAN SCIENCE TECHNOLOGY AGENCY JST	3	0.301
JIANGSU ACADEMY OF AGRICULTURAL SCIENCES	3	0.301
JIANGSU NORMAL UNIVERSITY	3	0.301
JIANGSU UNIVERSITY	3	0.301
JOHNS HOPKINS UNIVERSITY	3	0.301
JUSTUS LIEBIG UNIVERSITY GIESSEN	3	0.301
LANZHOU UNIVERSITY	3	0.301
MASSACHUSETTS INSTITUTE OF TECHNOLOGY MIT	3	0.301
MEDICAL UNIVERSITY OF VIENNA	3	0.301
MINIST AGR	3	0.301
MINIST HLTH	3	0.301
NATIONAL CHIAO TUNG UNIVERSITY	3	0.301
NATIONAL UNIVERSITY OF SCIENCES TECHNOLOGY PAKISTAN	3	0.301
NINGXIA MEDICAL UNIVERSITY	3	0.301
RIKEN	3	0.301
RUHR UNIVERSITY BOCHUM	3	0.301
SHANGHAI KEY LAB CHILDRENS DIGEST NUTR	3	0.301
SHANGHAI UNIVERSITY OF TRADITIONAL CHINESE MEDICINE	3	0.301
SHENZHEN UNIVERSITY	3	0.301

Table S2: Continued

Institutions	Record count	% of 998
STATE KEY LABORATORY OF RESPIRATORY DISEASE	3	0.301
UNIVERSIDADE FEDERAL DO PARA	3	0.301
UNIVERSITY OF CALIFORNIA LOS ANGELES	3	0.301
UNIVERSITY OF CALIFORNIA RIVERSIDE	3	0.301
UNIVERSITY OF KENTUCKY	3	0.301
UNIVERSITY OF NEW ENGLAND USA	3	0.301
UNIVERSITY OF QUEENSLAND	3	0.301
UNIVERSITY OF SOUTHERN DENMARK	3	0.301
UNIVERSITY OF VIENNA	3	0.301
UNIVERSITY OF VIRGINIA	3	0.301
UNIVERSITY SYSTEM OF GEORGIA	3	0.301
VANDERBILT UNIVERSITY	3	0.301
WANNAN MEDICAL COLLEGE	3	0.301
WEIFANG MEDICAL UNIVERSITY	3	0.301
XUZHOU CENT HOSP	3	0.301
YORK UNIVERSITY CANADA	3	0.301
YUNNAN KEY LAB VACCINE RES DEV SEVERE INFECT DI	3	0.301
ZHEJIANG ACADEMY OF AGRICULTURAL SCIENCES	3	0.301
ZHEJIANG CANCER HOSPITAL	3	0.301
181 HOSP PLA	2	0.2
ACADEMY OF MILITARY MEDICAL SCIENCES CHINA	2	0.2
AGENCY FOR SCIENCE TECHNOLOGY RESEARCH ASTAR	2	0.2
BEIJING ACADEMY OF AGRICULTURE FORESTRY	2	0.2
BEIJING NEUROSURGICAL INSTITUTE	2	0.2
BERLIN INST HLTH	2	0.2
BINZHOU MEDICAL UNIVERSITY	2	0.2
CANGZHOU CENT HOSP	2	0.2
CEA	2	0.2
CENT HOSP PANYU DIST	2	0.2
CENTRE FOR CANCER BIOLOGY	2	0.2
CESHHAR ZIMBABWE	2	0.2
CHINA NATL CLIN RES CTR NEUROL DIS	2	0.2
CHINESE CENTER FOR DISEASE CONTROL PREVENTION	2	0.2

Table S2: Continued

Institutions	Record count	% of 998
COLLABORAT INNOVAT CTR BIOTHERAPY	2	0.2
COMMONWEALTH SCIENTIFIC INDUSTRIAL RESEARCH ORGANISATION CSIRO	2	0.2
CSIC INSTITUTO DE PARASITOLOGIA Y BIOMEDICINA LOPEZ NEYRA IPBLN	2	0.2
CSIC JA USE INSTITUTO DE BIOMEDICINA DE SEVILLA IBIS	2	0.2
CSIR CENTRAL DRUG RESEARCH INSTITUTE	2	0.2
CTR SEXUAL HLTH HIV AIDS RES CESHHAR	2	0.2
DARTMOUTH COLLEGE	2	0.2
DUKE UNIVERSITY	2	0.2
EAST CHINA UNIVERSITY OF SCIENCE TECHNOLOGY	2	0.2
EMORY UNIVERSITY	2	0.2
FIRST PEOPLES HOSP YANCHENG CITY	2	0.2
FONDAZIONE CENCI BOLOGNETTI	2	0.2
FRAUNHOFER GESELLSCHAFT	2	0.2
FUJIAN AGRICULTURE FORESTRY UNIVERSITY	2	0.2
GARVAN INSTITUTE OF MEDICAL RESEARCH	2	0.2
GOLD CROSS AMBULANCE SERV	2	0.2
GUANGDONG LAB ANIM MONITORING INST	2	0.2
GUANGXI MEDICAL UNIVERSITY	2	0.2
GUANGXI UNIVERSITY OF CHINESE MEDICINE	2	0.2
GUILIN MEDICAL UNIVERSITY	2	0.2
HANNOVER MEDICAL SCHOOL	2	0.2
HEBEI UNIVERSITY OF ENGINEERING	2	0.2
HEILONGJIANG PROV HOSP	2	0.2
HENAN AGRICULTURAL UNIVERSITY	2	0.2
HENAN UNIVERSITY OF SCIENCE TECHNOLOGY	2	0.2
HILLSBOROUGH CTY FIRE RESCUE	2	0.2
HONG KONG UNIVERSITY OF SCIENCE TECHNOLOGY	2	0.2
HUNAN AGRICULTURAL UNIVERSITY	2	0.2
HUNAN UNIVERSITY OF CHINESE MEDICINE	2	0.2
ICAHN SCHOOL OF MEDICINE AT MOUNT SINAI	2	0.2
INSTITUT NATIONAL DE LA RECHERCHE AGRONOMIQUE INRA	2	0.2
INSTITUT NATIONAL DE LA SANTE ET DE LA RECHERCHE MEDICALE INSER	2	0.2
INSTITUTE OF ANIMAL SCIENCE IAS	2	0.2

Table S2: Continued

Institutions	Record count	% of 998
INSTITUTE OF CHEMICAL BIOLOGY FUNDAMENTAL MEDICINE SIBERIAN BRA	2	0.2
INSTITUTE OF HYDROBIOLOGY CAS	2	0.2
INSTITUTE OF PLANT PROTECTION IPP	2	0.2
IOWA STATE UNIVERSITY	2	0.2
IRCCS POLICLINICO SAN DONATO	2	0.2
ISTITUTO DI BIOLOGIA E PATOLOGIA MOLECOLARI IBPM CNR	2	0.2
ISTITUTO ITALIANO DI TECNOLOGIA IIT	2	0.2
JIANGSU CANC HOSP	2	0.2
JIANGSU INST CANC RES	2	0.2
JIANGSU PROV HOSP	2	0.2
JIANGXI MATERNAL CHILD HLTH HOSP	2	0.2
JIANGXI PROV BLOOD CTR	2	0.2
JINAN CENTRAL HOSPITAL	2	0.2
KAOHSIUNG MEDICAL UNIVERSITY	2	0.2
KAROLINSKA INSTITUTET	2	0.2
KING ABDULLAH UNIVERSITY OF SCIENCE TECHNOLOGY	2	0.2
KUNMING INSTITUTE OF ZOOLOGY	2	0.2
KYOTO UNIVERSITY	2	0.2
LANCASTER UNIVERSITY	2	0.2
LIAOCHENG PEOPLES HOSP	2	0.2
LIAONING RES CTR TRANSLAT MED NERVOUS SYST DIS	2	0.2
LINYI CENT HOSP	2	0.2
LOMA LINDA UNIVERSITY	2	0.2
MAYO CLINIC	2	0.2
MEDICAL COLLEGE OF WISCONSIN	2	0.2
METROWEST MED CTR	2	0.2
MINIST HLTH CHILD CARE	2	0.2
MINISTRY OF HEALTH OF THE RUSSIAN FEDERATION	2	0.2
MUDANJIANG MEDICAL UNIVERSITY	2	0.2
MUNICH HEART ALLIANCE	2	0.2
NATIONAL CHENG KUNG UNIVERSITY	2	0.2
NATIONAL CHUNG HSING UNIVERSITY	2	0.2
NATIONAL TAIWAN UNIVERSITY	2	0.2

Table S2: Continued

Institutions	Record count	% of 998
NINGBO FIRST HOSP	2	0.2
NORRIS COTTON CANCER CENTER	2	0.2
PLA	2	0.2
POPULAT SERV INT	2	0.2
RADBOUD UNIVERSITY NIJMEGEN	2	0.2
REG AMBULANCE SERV GELDERLAND ZUID	2	0.2
SA PATHOLOGY	2	0.2
SAPIENZA UNIVERSITY ROME	2	0.2
SEAHORSE COASTAL CONSULTING	2	0.2
SEOUL NATIONAL UNIVERSITY	2	0.2
SHANGHAI UNIV MED HLTH SCI	2	0.2
SICHUAN AGRICULTURAL UNIVERSITY	2	0.2
SOUTHWEST MEDICAL UNIVERSITY	2	0.2
SOUTHWEST UNIVERSITY CHINA	2	0.2
ST JOSEF HOSP	2	0.2
STELLENBOSCH UNIVERSITY	2	0.2
STOCKHOLM UNIVERSITY	2	0.2
TAIZHOU PEOPLES HOSP	2	0.2
TENNESSEE STATE UNIVERSITY	2	0.2
THE SANTA FE INSTITUTE	2	0.2
THEDA CLARK REG MED CTR	2	0.2
TIANJIN HUANHU HOSP	2	0.2
TONGDE HOSP ZHEJIANG PROV	2	0.2
TOYOHASHI UNIVERSITY OF TECHNOLOGY	2	0.2
TSINGHUA UNIVERSITY	2	0.2
TULANE UNIVERSITY	2	0.2
TYGERBERG HOSPITAL	2	0.2
U S ARMY CORPS OF ENGINEERS	2	0.2
U S ARMY ENGINEER RESEARCH DEVELOPMENT CENTER ERDC	2	0.2
ULM UNIVERSITY	2	0.2
UNITED STATES ARMY	2	0.2
UNITED STATES DEPARTMENT OF AGRICULTURE USDA	2	0.2
UNITED STATES DEPARTMENT OF DEFENSE	2	0.2

Table S2: Continued

Institutions	Record count	% of 998
UNIVERSITE PARIS SACLAY	2	0.2
UNIVERSITE PARIS SACLAY COMUE	2	0.2
UNIVERSITI KEBANGSAAN MALAYSIA	2	0.2
UNIVERSITY COLLEGE LONDON	2	0.2
UNIVERSITY OF ARIZONA	2	0.2
UNIVERSITY OF CALIFORNIA SAN DIEGO	2	0.2
UNIVERSITY OF CAMBRIDGE	2	0.2
UNIVERSITY OF COLORADO BOULDER	2	0.2
UNIVERSITY OF COLORADO DENVER	2	0.2
UNIVERSITY OF CONNECTICUT	2	0.2
UNIVERSITY OF ILLINOIS CHICAGO	2	0.2
UNIVERSITY OF ILLINOIS CHICAGO HOSPITAL	2	0.2
UNIVERSITY OF ILLINOIS SYSTEM	2	0.2
UNIVERSITY OF LEICESTER	2	0.2
UNIVERSITY OF LONDON	2	0.2
UNIVERSITY OF MELBOURNE	2	0.2
UNIVERSITY OF MICHIGAN	2	0.2
UNIVERSITY OF MICHIGAN SYSTEM	2	0.2
UNIVERSITY OF MINNESOTA SYSTEM	2	0.2
UNIVERSITY OF MINNESOTA TWIN CITIES	2	0.2
UNIVERSITY OF OKLAHOMA NORMAN	2	0.2
UNIVERSITY OF OKLAHOMA SYSTEM	2	0.2
UNIVERSITY OF OSLO	2	0.2
UNIVERSITY OF PITTSBURGH	2	0.2
UNIVERSITY OF ROCHESTER	2	0.2
UNIVERSITY OF SEVILLA	2	0.2
UNIVERSITY OF SOUTH AUSTRALIA	2	0.2
UNIVERSITY OF TEXAS MEDICAL BRANCH GALVESTON	2	0.2
UNIVERSITY OF TEXAS SOUTHWESTERN MEDICAL CENTER DALLAS	2	0.2
UNIVERSITY OF ZIMBABWE	2	0.2
UPPSALA UNIVERSITY	2	0.2
UTRECHT UNIVERSITY	2	0.2
UTRECHT UNIVERSITY MEDICAL CENTER	2	0.2
VRIJE UNIVERSITEIT AMSTERDAM	2	0.2

Table S2: Continued

Institutions	Record count	% of 998
WRIGHT STATE UNIVERSITY DAYTON	2	0.2
WUHAN INSTITUTE OF VIROLOGY CAS	2	0.2
XI AN MEDICAL UNIVERSITY	2	0.2
XINYANG NORMAL UNIVERSITY	2	0.2
YANGTZE UNIVERSITY	2	0.2
YUEWEI EDIBLE FUNGI TECHNOL CO LTD	2	0.2
YUNNAN PROV KEY LAB VECTOR BORNE DIS CONTROL RE	2	0.2
ZAOZHUANG MUNICIPAL HOSP	2	0.2
ZHEJIANG CHINESE MEDICAL UNIVERSITY	2	0.2
ZHEJIANG PROVINCIAL PEOPLE S HOSPITAL	2	0.2
ZHENGXING HOSP	2	0.2
ZHONGKAI UNIVERSITY OF AGRICULTURE ENGINEERING	2	0.2
ZOLL MED CORP	2	0.2
141 HOSP	1	0.1
302 MILITARY HOSPITAL OF CHINA	1	0.1
323 HOSP PLA	1	0.1
4 HOSP 1946 JINAN SHANDONG	1	0.1
88 HOSP PEOPLES LIBERAT ARMY	1	0.1
ABLIFE INC	1	0.1
ADAM MICKIEWICZ UNIVERSITY	1	0.1
AFFILIATED HOSP QINGDAO UNIV	1	0.1
AGENCY FOR THE ASSESSMENT APPLICATION OF TECHNOLOGY BPPT	1	0.1
ALBERT EINSTEIN COLLEGE OF MEDICINE	1	0.1
ALBIHNS ZACCO STOCKHOLM	1	0.1
ANHUI 2 PROV PEOPLES HOSP	1	0.1
ANHUI AGRICULTURAL UNIVERSITY	1	0.1
APPLETON FIRE DEPT	1	0.1
ARIZONA ALZHEIMERS CONSORTIUM	1	0.1
ARIZONA STATE UNIVERSITY	1	0.1
ATMOSPHERIC ENVIRONMENTAL RESEARCH	1	0.1
AUGUSTA UNIVERSITY	1	0.1
AV UNIV S N COL CHAMILPA	1	0.1
BAR ILAN UNIVERSITY	1	0.1

Table S2: Continued

Institutions	Record count	% of 998
GANSU AGRICULTURAL UNIVERSITY	1	0.1
GANSU PROV HOSP TRADIT CHINESE MED	1	0.1
GANSU RES CTR SWINE PROD ENGN TECHNOL	1	0.1
GANZHOU PEOPLES HOSP	1	0.1
GAOMI PEOPLES HOSP	1	0.1
GEN HOSP DAQING OIL FIELD	1	0.1
GEN HOSP JINAN MIL COMMAND	1	0.1
GEN HOSP SHENYANG MIL AREA COMMAND	1	0.1
GENOME INSTITUTE OF SINGAPORE GIS	1	0.1
GEORGE INSTITUTE FOR GLOBAL HEALTH	1	0.1
GEORGE MASON UNIVERSITY	1	0.1
GEORGIA INSTITUTE OF TECHNOLOGY	1	0.1
GERMAN CANCER RESEARCH CENTER DKFZ	1	0.1
GERMAN REGISTRY CONGENITAL HEART DEFECTS	1	0.1
GERSTNER SLOAN KETTERING GRAD PROGRAM BIOMED SCI	1	0.1
GISC	1	0.1
GOETHE UNIVERSITY FRANKFURT HOSPITAL	1	0.1
GONGLI HOSP PUDONG NEW AREA	1	0.1
GOPATH DIAGNOST LAB CO LTD 801	1	0.1
GOPATH LABS LLC	1	0.1
GRAND CHUTE FIRE DEPT	1	0.1
GUANG ZHOU MED UNIV	1	0.1
GUANGDONG CARDIOVASC INST	1	0.1
GUANGDONG HIGHER EDUC INST GUANDONG PROV	1	0.1
GUANGDONG KEY LAB ORTHOPAED TECHNOL IMPLANT MAT	1	0.1
GUANGDONG MED LAB ANIM CTR	1	0.1
GUANGDONG PROV KEY LAB AGROANIM GENOM MOL BREED	1	0.1
GUANGDONG PROV KEY LAB APPL BOT	1	0.1
GUANGDONG PROV KEY LAB MED MOL DIAGNOST	1	0.1
GUANGDONG PROV KEY LAB MOL TUMOR PATHOL	1	0.1
GUANGDONG PROV KEY LAB PITUITARY TUMOR	1	0.1
GUANGDONG SECOND PROV GEN HOSP	1	0.1
GUANGXI NORMAL UNIVERSITY	1	0.1

Table S2: Continued

Institutions	Record count	% of 998
GUANGZHOU GEN HOSP	1	0.1
GUANGZHOU GEN HOSP GUANGZHOU MIL COMMAND	1	0.1
GUANGZHOU GEN HOSP GUANGZHOU MIL REG	1	0.1
GUANGZHOU INST DERMATOL	1	0.1
GUANGZHOU MIL COMMAND	1	0.1
GUANGZHOU UNIVERSITY OF CHINESE MEDICINE	1	0.1
GUANGZHOU WOMEN CHILDRENS MED CTR	1	0.1
GUIZHOU PROV PEOPLES HOSP	1	0.1
GULLIVER PREPARATORY SCH	1	0.1
HAINAN ACAD AGR SCI	1	0.1
HAINAN MEDICAL UNIVERSITY	1	0.1
HAINAN PROV PEOPLES HOSP	1	0.1
HALLYM UNIVERSITY	1	0.1
HANGZHOU HOSP	1	0.1
HANGZHOU NORMAL UNIVERSITY	1	0.1
HARBIN INSTITUTE OF TECHNOLOGY	1	0.1
HARBIN MED UNIV CANC HOSP	1	0.1
HEBEI ENGN UNIV	1	0.1
HEBEI PROV HOSP TRADIT CHINESE MED	1	0.1
HEILONGJIANG ACAD TRADIT CHINESE MED	1	0.1
HEINRICH HEINE UNIVERSITY DUSSELDORF	1	0.1
HELMHOLTZ CENTER FOR ENVIRONMENTAL RESEARCH UFZ	1	0.1
HENAN ENGN LAB BIOENGN DRUG DEV	1	0.1
HENAN NORMAL UNIVERSITY	1	0.1
HEZE CTR DIS CONTROL PREVENT	1	0.1
HOSP JINGXING CTY	1	0.1
HOSPITAL INFANTIL DE MEXICO FEDERICO GOMEZ	1	0.1
HOSPITAL VALME	1	0.1
HOUSTON FIRE DEPT	1	0.1
HOWARD UNIVERSITY	1	0.1
HUAQIAO UNIVERSITY	1	0.1
HUBEI PROV ACAD PREVENT MED	1	0.1
HUBEI PROV KEY LAB ALLERGY IMMUNOL	1	0.1

Table S2: Continued

Institutions	Record count	% of 998
HUBEI UNIVERSITY OF ARTS SCIENCE	1	0.1
HUDSONALPHA INSTITUTE FOR BIOTECHNOLOGY	1	0.1
HUIZHOU HOSP TRADIT CHINESE MED	1	0.1
HUMANITAS CLIN RES CTR	1	0.1
HUMANITAS UNIVERSITY	1	0.1
HUNAN HYBRID RICE RES CTR	1	0.1
HUNAN PROV KEY LAB GENET IMPROVEMENT DOMEST ANIM	1	0.1
ILLINOIS COLL	1	0.1
INDIAN INST INFORMAT TECHNOL ALLAHABAD	1	0.1
INDIAN INSTITUTE OF CHEMICAL BIOLOGY	1	0.1
INDIAN INSTITUTE OF TECHNOLOGY IIT KHARAGPUR	1	0.1
INDIAN INSTITUTE OF TECHNOLOGY SYSTEM IIT SYSTEM	1	0.1
INDIAN NATIONAL CENTRE FOR OCEAN INFORMATION SERVICES INCOIS	1	0.1
INDIANA UNIVERSITY BLOOMINGTON	1	0.1
INDIANA UNIVERSITY SYSTEM	1	0.1
INFECT DIS HOSP BENGBU CITY	1	0.1
INNER MONGOLIA KEY LAB MONGOLIAN MED PHARMACOL CA	1	0.1
INNER MONGOLIA UNIVERSITY FOR NATIONALITIES	1	0.1
INRIA	1	0.1
INST BIOCOMPUTAT PHYS COMPLEX SYST	1	0.1
INST CARDIOVASC REGENERAT	1	0.1
INST VEGETABLE RES XINING	1	0.1
INSTITUT NATIONAL POLYTECHNIQUE DE GRENOBLE	1	0.1
INSTITUT UNIVERSITAIRE DE FRANCE	1	0.1
INSTITUTE OF BASIC MEDICAL SCIENCES CAMS	1	0.1
INSTITUTE OF BIOCHEMISTRY GENETICS OF UFA SCIENCE CENTRE OF THE	1	0.1
INSTITUTE OF BIOORGANIC CHEMISTRY OF THE POLISH ACADEMY OF SCIE	1	0.1
INSTITUTE OF BIOPHYSICS CAS	1	0.1
INSTITUTE OF BOTANY CAS	1	0.1
INSTITUTE OF COMPUTING TECHNOLOGY CAS	1	0.1
INSTITUTE OF HEMATOLOGY BLOOD DISEASES HOSPITAL CAMS	1	0.1
INSTITUTE OF LIFE SCIENCES INDIA ILS	1	0.1
INSTITUTE OF MOLECULAR CELL BIOLOGY	1	0.1

Table S2: Continued

Institutions	Record count	% of 998
INSTITUTE OF RADIATION MEDICINE CAMS	1	0.1
INSTITUTE OF ZOOLOGY CAS	1	0.1
INSTITUTO FEDERAL DO RIO GRANDE DO SUL IFRS	1	0.1
INSTITUTO POLITECNICO NACIONAL MEXICO	1	0.1
INSTITUTUL CLINIC FUNDENI	1	0.1
IRCCS SANTA LUCIA	1	0.1
ISTANBUL UNIVERSITY	1	0.1
ISTITUTO DI BIOLOGIA CELLULARE E NEUROBIOLOGIA IBCN CNR	1	0.1
ISTITUTO DI CHIMICA DELLA MATERIA CONDENSATA E DI TECNOLOGIE PE	1	0.1
IULIU HATIEGANU UNIVERSITY OF MEDICINE PHARMACY	1	0.1
JACKSON STATE UNIVERSITY	1	0.1
JAPAN AEROSPACE EXPLORATION AGENCY JAXA	1	0.1
JIAMUSI UNIVERSITY	1	0.1
JIANGNAN UNIVERSITY	1	0.1
JIANGSU HLTH VOCAT COLL	1	0.1
JIANGSU INST PLANNED PARENTHOOD RES	1	0.1
JIANGSU JIANKANG VOCAT COLL	1	0.1
JIANGSU KEY LAB HORT CROP GENET IMPROVEMENT	1	0.1
JIANGSU KEY LAB PREVENT TRANSLAT MED GENET DIS	1	0.1
JIANGSU PROV PEOPLES HOSP	1	0.1
JIANGSU VOCAT COLL MED	1	0.1
JIANGSU XILAIYUAN ECOL AGR CO LTD TAIZHOU	1	0.1
JIANGXI PROV CHILDRENS HOSP	1	0.1
JILIN MEDICAL UNIVERSITY	1	0.1
JILIN PROV TUMOR HOSP	1	0.1
JIMEI UNIVERSITY	1	0.1
JINAN MATERN CHILD CARE HOSP	1	0.1
JINAN UNIV	1	0.1
JINING 1 PEOPLES HOSP	1	0.1
JINING MEDICAL UNIVERSITY	1	0.1
JOHNS HOPKINS BLOOMBERG SCHOOL OF PUBLIC HEALTH	1	0.1
JOHNS HOPKINS MEDICINE	1	0.1
JOHNS HOPKINS ONCOLOGY CENTER	1	0.1

Table S2: Continued

**Institutions**	**Record count**	**% of 998**
KALYANI UNIVERSITY	1	0.1
KAROLINSKA UNIVERSITY HOSPITAL	1	0.1
KEIO UNIVERSITY	1	0.1
KEY LAB ANIM HLTH AQUACULTURE ENVIRONM CONTROL	1	0.1
KEY LAB NEUROONCOL LIAONING PROV	1	0.1
KEY LAB REPROD MED EMBRYO GANSU	1	0.1
KU LEUVEN	1	0.1
KUNMING ANGEL WOMENS CHILDRENS HOSP	1	0.1
KUNMING ENGN TECHNOL CTR DIGEST DIS	1	0.1
KUNSAN NATL UNIV	1	0.1
KUWAIT MED GENET CTR	1	0.1
KYUSHU UNIVERSITY	1	0.1
LA TROBE UNIVERSITY	1	0.1
LAKE ERIE COLL	1	0.1
LAKE ERIE COLL OSTEOPATH MED	1	0.1
LAWRENCE BERKELEY NATIONAL LABORATORY	1	0.1
LI HUI LI HOSP	1	0.1
LIBIN CARDIOVASCULAR INSTITUTE OF ALBERTA	1	0.1
LINGNAN NORMAL UNIVERSITY	1	0.1
LINYI PEOPLES HOSP	1	0.1
LIYANG PEOPLES HOSP	1	0.1
LOMONOSOV MOSCOW STATE UNIVERSITY	1	0.1
LONDON HEALTH SCIENCES CENTRE	1	0.1
LONDON SCHOOL OF HYGIENE TROPICAL MEDICINE	1	0.1
LOUISIANA STATE UNIVERSITY HEALTH SCIENCES CENTER NEW ORLEANS	1	0.1
LUDONG UNIVERSITY	1	0.1
LUDWIG BOLTZMANN INSTITUTE	1	0.1
LUDWIG MAXIMILIAN UNIV MUNICH LMU	1	0.1
LUXEMBOURG INSTITUTE OF HEALTH	1	0.1
MAASTRICHT UNIVERSITY	1	0.1
MACKAY JUNIOR COLLEGE OF MEDICINE NURSING MANAGEMENT	1	0.1
MACKAY MEDICAL COLLEGE	1	0.1
MACKAY MEMORIAL HOSPITAL	1	0.1
MACQUARIE UNIVERSITY	1	0.1
MADURAI KAMARAJ UNIVERSITY	1	0.1

Table S2: Continued

Institutions	Record count	% of 998
MED CTR ASSESSMENT PREVENT TREATMENT BONE JOI	1	0.1
MEHARRY MEDICAL COLLEGE	1	0.1
MEMORIAL SLOAN KETTERING CANCER CENTER	1	0.1
MICHIGAN STATE UNIVERSITY	1	0.1
MINISTRY OF EARTH SCIENCES MOES INDIA	1	0.1
MINISTRY OF EDUCATION CHINA	1	0.1
MONASH UNIV MALAYSIA	1	0.1
MUNICIPAL AMBULANCE SERV VIENNA	1	0.1
NAGOYA UNIVERSITY	1	0.1
NANJING CHEST HOSP	1	0.1
NANJING FIRST HOSP	1	0.1
NANJING FOREIGN LANGUAGE SCH	1	0.1
NANJING GENERAL HOSPITAL	1	0.1
NANJING MATERN CHILD HLTH CARE HOSP	1	0.1
NANJING UNIVERSITY OF POSTS TELECOMMUNICATIONS	1	0.1
NANTONG TUMOR HOSP	1	0.1
NASA GODDARD SPACE FLIGHT CENTER	1	0.1
NATIONAL AERONAUTICS SPACE ADMINISTRATION NASA	1	0.1
NATIONAL HEALTH RESEARCH INSTITUTES TAIWAN	1	0.1
NATIONAL INSTITUTE OF ADVANCED INDUSTRIAL SCIENCE TECHNOLOGY AI	1	0.1
NATIONAL INSTITUTE OF TECHNOLOGY CALICUT	1	0.1
NATIONAL OCEANIC ATMOSPHERIC ADMIN NOAA USA	1	0.1
NATIONAL YANG MING UNIVERSITY	1	0.1
NATL BIOTECHNOL CTR CSIC	1	0.1
NATL CLIN RES CTR DIGEST DIS	1	0.1
NATL CLIN RES CTR STOMATOL	1	0.1
NATL CTR PROT SCI	1	0.1
NATL ENGN CTR BIOCHIP SHANGHAI	1	0.1
NATL HLTH FAMILY PLANNING COMMISS	1	0.1
NATL LOCAL JOINT ENGN RES CTR LIVESTOCK BREEDING	1	0.1
NATL LOCAL UNITED ENGN RES CTR SANQI RESOURCES	1	0.1
NEENAH MENASHA FIRE DEPT	1	0.1
NEUROSCIENCE RESEARCH AUSTRALIA	1	0.1

Table S2: Continued

Institutions	Record count	% of 998
NEW YORK UNIVERSITY	1	0.1
NEWCASTLE UNIVERSITY UK	1	0.1
NHS BLOOD TRANSPLANT NHSBT	1	0.1
NIH NATIONAL CANCER INSTITUTE NCI	1	0.1
NIH NATIONAL LIBRARY OF MEDICINE NLM	1	0.1
NINGBO 2 HOSP	1	0.1
NINGBO 9 HOSP	1	0.1
NINGHAI CTY CTR DIS CONTROL PREVENT	1	0.1
NORTH CAROLINA STATE UNIVERSITY	1	0.1
NORTH SICHUAN MEDICAL UNIVERSITY	1	0.1
NORTHEAST AGRICULTURAL UNIVERSITY CHINA	1	0.1
NORTHEASTERN UNIVERSITY	1	0.1
NORTHERN INTERTRIBAL HLTH AUTHOR	1	0.1
NORWEGIAN UNIVERSITY OF SCIENCE TECHNOLOGY NTNU	1	0.1
NRC INSTITUTE OF IMMUNOLOGY FMBA OF RUSSIA	1	0.1
NYANZA REPROD HLTH SOC	1	0.1
OCEAN UNIVERSITY OF CHINA	1	0.1
ODENSE UNIVERSITY HOSPITAL	1	0.1
OHIO STATE UNIVERSITY	1	0.1
OIL CROPS RESEARCH INSTITUTE	1	0.1
OKAYAMA UNIVERSITY	1	0.1
OSAKA UNIVERSITY	1	0.1
PEIHUA UNIV	1	0.1
PEOPLES HOSP GAOZHOU	1	0.1
PEOPLES HOSP YANGZHONG	1	0.1
POLISH ACADEMY OF SCIENCES	1	0.1
POZNAN UNIVERSITY OF MEDICAL SCIENCES	1	0.1
PREGNANCY RES CTR	1	0.1
PURDUE UNIVERSITY	1	0.1
PURDUE UNIVERSITY SYSTEM	1	0.1
QIAGEN GMBH	1	0.1
QIANNAN NORMAL UNIV NATIONALITIES	1	0.1
QINGDAO HISER MED CTR	1	0.1

Table S2: Continued

Institutions	Record count	% of 998
QUEENSLAND UNIVERSITY OF TECHNOLOGY QUT	1	0.1
REPROCELL USA	1	0.1
RES CTR AIR POLLUT HLTH	1	0.1
RESEARCH CENTER JULICH	1	0.1
RICE UNIVERSITY	1	0.1
RIGSHOSPITALET	1	0.1
RIKKYO UNIVERSITY	1	0.1
ROYAL WOMENS HOSP	1	0.1
RUDJER BOSKOVIC INSTITUTE	1	0.1
RUSSIAN ACADEMY OF MEDICAL SCIENCE	1	0.1
SAARLAND UNIVERSITY	1	0.1
SCH PUBL HLTH ANDRIJA STAMPAR	1	0.1
SCOTTISH NATL BLOOD TRANSFUS SERV	1	0.1
SECHENOV FIRST MOSCOW STATE MEDICAL UNIVERSITY	1	0.1
SECOND PEOPLE S HOSPITAL OF SHENZHEN	1	0.1
SECOND PEOPLES HOSP SHAANXI PROV	1	0.1
SECOND PEOPLES HOSP YUNNAN PROV	1	0.1
SEOUL NATIONAL UNIVERSITY HOSPITAL	1	0.1
SHAANXI UNIVERSITY OF CHINESE MEDICINE	1	0.1
SHAANXI UNIVERSITY OF SCIENCE TECHNOLOGY	1	0.1
SHANDONG MED COLL	1	0.1
SHANGHAI BIOTECHNOL CORP	1	0.1
SHANGHAI CENTER FOR DISEASE CONTROL PREVENTION	1	0.1
SHANGHAI INST HEAD TRAUMA	1	0.1
SHANGHAI KEY LAB REPROD MED	1	0.1
SHANGHAI KEY LAB STOMATOL	1	0.1
SHANGHAI RES INST STOMATOL	1	0.1
SHANGHAI TENTH PEOPLE S HOSP	1	0.1
SHANGQIU NORMAL UNIVERSITY	1	0.1
SHANTOU UNIVERSITY	1	0.1
SHANXI AGRICULTURAL UNIVERSITY	1	0.1
SHANXI MEDICAL UNIVERSITY	1	0.1
SHANXI PEOPLE S HOSPITAL	1	0.1

Table S2: Continued

Institutions	Record count	% of 998
SHENZHEN LONGHUA DIST CENT HOSP	1	0.1
SHENZHEN PEOPLES HOSP	1	0.1
SHIRAZ UNIVERSITY	1	0.1
SHOUGUANG PEOPLE HOSP	1	0.1
SICHUAN PROVINCIAL PEOPLE S HOSPITAL	1	0.1
SIMUNYE PRIMARY HLTH CARE	1	0.1
SIXTH PEOPLES HOSP	1	0.1
SORBONNE UNIVERSITE	1	0.1
SOUTH CENTRAL UNIVERSITY FOR NATIONALITIES	1	0.1
SOUTH CHINA BOTANICAL GARDEN CAS	1	0.1
SOUTH CHINA COLLABORAT INNOVAT CTR POULTRY DIS CO	1	0.1
SOUTH DAKOTA STATE UNIVERSITY	1	0.1
SOUTHERN UNIVERSITY OF SCIENCE TECHNOLOGY	1	0.1
SOUTHWEST UNIVERSITY FOR NATIONALITIES CHINA	1	0.1
SPANISH NETWORK MULTIPLE SCLEROSIS	1	0.1
SPIN LTDA EPP	1	0.1
ST VINCENTS HOSPITAL SYDNEY	1	0.1
STANFORD BIOMED INFORMAT TRAINING PROGRAM	1	0.1
STANFORD DEPT BIOCHEM	1	0.1
STATE KEY LAB CULTIVAT BASE CELL DIFFERENTIAT REG	1	0.1
STATE KEY LAB REPROD MED	1	0.1
STATE UNIVERSITY OF NEW YORK SUNY BUFFALO	1	0.1
STATE UNIVERSITY OF NEW YORK SUNY SYSTEM	1	0.1
STATE UNIVERSITY SYSTEM OF FLORIDA	1	0.1
SUMC	1	0.1
SUZHOU KEY LAB MOL CANC GENET	1	0.1
TAIZHOU UNIVERSITY	1	0.1
TEXAS A M UNIVERSITY COLLEGE STATION	1	0.1
TEXAS A M UNIVERSITY SYSTEM	1	0.1
THERMO FISHER SCIENTIFIC	1	0.1
THIRD HOSP NANCHANG	1	0.1
THIRD PEOPLES HOSP ZHENJIANG	1	0.1
TIANJIN INTERNATIONAL JOINT ACADEMY OF BIOMEDICINE	1	0.1

Table S2: Continued

**Institutions**	**Record count**	**% of 998**
TIANJIN UNIVERSITY	1	0.1
TORONTO GENERAL HOSPITAL	1	0.1
TRADIT CHINESE MED HOSP BEIJING HUAIROU BEIJING	1	0.1
TRANSLATIONAL GENOMICS RESEARCH INSTITUTE	1	0.1
TRINITY COLLEGE DUBLIN	1	0.1
TUMOR HOSP YUNNAN PROV	1	0.1
TUMOUR HOSP LIAOCHENG	1	0.1
UC3M BS INST FINANCIAL BIG DATA IFIBID	1	0.1
UCLA JONSSON COMPREHENSIVE CANCER CENTER	1	0.1
UIT THE ARCTIC UNIVERSITY OF TROMSO	1	0.1
UMEA UNIVERSITY	1	0.1
UNICANCER	1	0.1
UNICEF	1	0.1
UNITED STATES DEPARTMENT OF ENERGY DOE	1	0.1
UNITED STATES DEPARTMENT OF THE INTERIOR	1	0.1
UNITED STATES GEOLOGICAL SURVEY	1	0.1
UNIV NORTH TEXAS	1	0.1
UNIVERSIDAD CARLOS III DE MADRID	1	0.1
UNIVERSIDAD CATOLICA DEL NORTE	1	0.1
UNIVERSIDAD DE CHILE	1	0.1
UNIVERSIDADE FEDERAL DE SAO PAULO UNIFESP	1	0.1
UNIVERSITE CLERMONT AUVERGNE ASSOCIES	1	0.1
UNIVERSITE DE LYON COMUE	1	0.1
UNIVERSITE DE MONTPELLIER	1	0.1
UNIVERSITI PUTRA MALAYSIA	1	0.1
UNIVERSITI SAINS MALAYSIA	1	0.1
UNIVERSITY COLLEGE DUBLIN	1	0.1
UNIVERSITY HEALTH NETWORK TORONTO	1	0.1
UNIVERSITY HOSPITAL DONOSTIA	1	0.1

Table S2: Continued

Institutions	Record count	% of 998
UNIVERSITY OF BERN	1	0.1
UNIVERSITY OF CAGLIARI	1	0.1
UNIVERSITY OF CALGARY	1	0.1
UNIVERSITY OF CALIFORNIA BERKELEY	1	0.1
UNIVERSITY OF CALIFORNIA DAVIS	1	0.1
UNIVERSITY OF CALIFORNIA SAN FRANCISCO	1	0.1
UNIVERSITY OF CAPE TOWN	1	0.1
UNIVERSITY OF CHICAGO	1	0.1
UNIVERSITY OF COLOGNE	1	0.1
UNIVERSITY OF COLORADO HEALTH SCIENCE CENTER	1	0.1
UNIVERSITY OF ERLANGEN NUREMBERG	1	0.1
UNIVERSITY OF EXETER	1	0.1
UNIVERSITY OF FERRARA	1	0.1
UNIVERSITY OF FLORIDA	1	0.1
UNIVERSITY OF GEORGIA	1	0.1
UNIVERSITY OF GLASGOW	1	0.1
UNIVERSITY OF GOTTINGEN	1	0.1
UNIVERSITY OF GUELPH	1	0.1
UNIVERSITY OF HAWAII MANOA	1	0.1
UNIVERSITY OF HAWAII SYSTEM	1	0.1
UNIVERSITY OF HYDERABAD	1	0.1
UNIVERSITY OF KIEL	1	0.1
UNIVERSITY OF LAUSANNE	1	0.1
UNIVERSITY OF LEEDS	1	0.1
UNIVERSITY OF LJUBLJANA	1	0.1

Table S2: Continued

Institutions	Record count	% of 998
UNIVERSITY OF MIAMI	1	0.1
UNIVERSITY OF MILAN	1	0.1
UNIVERSITY OF NEBRASKA LINCOLN	1	0.1
UNIVERSITY OF NEBRASKA SYSTEM	1	0.1
UNIVERSITY OF NOTRE DAME	1	0.1
UNIVERSITY OF PADUA	1	0.1
UNIVERSITY OF PALERMO	1	0.1
UNIVERSITY OF REGENSBURG	1	0.1
UNIVERSITY OF ROME TOR VERGATA	1	0.1
UNIVERSITY OF SANTIAGO DE COMPOSTELA	1	0.1
UNIVERSITY OF SOUTHERN CALIFORNIA	1	0.1
UNIVERSITY OF SUSSEX	1	0.1
UNIVERSITY OF SYDNEY	1	0.1
UNIVERSITY OF TECHNOLOGY SYDNEY	1	0.1
UNIVERSITY OF TEXAS SCHOOL PUBLIC HEALTH	1	0.1
UNIVERSITY OF TOKYO	1	0.1
UNIVERSITY OF TOLEDO	1	0.1
UNIVERSITY OF TOYAMA	1	0.1
UNIVERSITY OF TSUKUBA	1	0.1
UNIVERSITY OF WASHINGTON	1	0.1
UNIVERSITY OF WASHINGTON SEATTLE	1	0.1
UNIVERSITY OF WISCONSIN MADISON	1	0.1

Table S2: Continued

Institutions	Record count	% of 998
UNIVERSITY OF YORK UK	1	0.1
UNIVERSITY OF ZAGREB	1	0.1
UNIVERSITY TOWN OF SHENZHEN	1	0.1
VETAGRO SUP	1	0.1
VIRGEN DEL ROCIO UNIVERSITY HOSPITAL	1	0.1
VIRGINIA COMMONWEALTH UNIVERSITY	1	0.1
VIRGINIA INSTITUTE OF MARINE SCIENCE	1	0.1
VITA SALUTE SAN RAFFAELE UNIVERSITY	1	0.1
VU UNIVERSITY MEDICAL CENTER	1	0.1
WAKE FOREST UNIVERSITY	1	0.1
WARMAN MED COLL	1	0.1
WEI FANG TRADIT CHINESE HOSP	1	0.1
WEIFANG MENTAL HLTH CTR	1	0.1
WEIFANG TRADIT CHINESE HOSP	1	0.1
WEILL CORNELL MEDICAL COLLEGE QATAR	1	0.1
WENZHOU PEOPLES HOSP	1	0.1
WESTERN UNIVERSITY UNIVERSITY OF WESTERN ONTARIO	1	0.1
WHITEHEAD INSTITUTE	1	0.1
WILLIAM MARY	1	0.1
WILLIAM S MIDDLETON MEM VA HOSP	1	0.1
WUHAN HOSP PREVENT TREATMENT OCCUPAT DIS	1	0.1
WUHAN TONGJI REPROD MED HOSP	1	0.1
WUXI PLA 101 HOSP	1	0.1

Table S2: Continued

Institutions	Record count	% of 998
XIAMEN MUNICIPAL KEY LAB GASTROINTESTINAL ONCOL	1	0.1
XIANGTAN BUR ANIM HUSB VET MED AQUAT PROD	1	0.1
XIANYANG CENT HOSP	1	0.1
XINJIANG MEDICAL UNIVERSITY	1	0.1
XINXIANG MEDICAL UNIVERSITY	1	0.1
XUHUI CENT HOSP	1	0.1
YALE UNIVERSITY	1	0.1
YANAN PEOPLES HOSP	1	0.1
YANAN UNIVERSITY	1	0.1
YANTAI UNIVERSITY	1	0.1
YESHIVA UNIVERSITY	1	0.1
YIDU CENT HOSP WEIFANG	1	0.1
YOKOHAMA CITY UNIVERSITY	1	0.1
YUNNAN ANIM SCI VET INST	1	0.1
YUNNAN INNOVAT TEAM STANDARDIZAT APPLICAT RES T	1	0.1
YUNNAN UNIV TRADIT CHINESE MED	1	0.1
ZHEJIANG FIRST HOSP	1	0.1
ZHEJIANG PROV KEY LAB OPHTHALMOL	1	0.1
ZHEJIANG PROV KEY LAB PATHOPHYSIOL TECHNOL RES	1	0.1
ZHEJIANG TAIZHOU MUNICIPAL HOSP	1	0.1
ZHEJIANG UNIVERSITY OF SCIENCE TECHNOLOGY	1	0.1
ZIBO CENT HOSP	1	0. 1
ZUNYI MEDICAL COLLEGE	1	0. 1
ZUSE INST BERLIN	1	0. 1

Table S3: All journals that published research related to circular RNA

Source titles	Record count	% of 998
BIOCHEMICAL AND BIOPHYSICAL RESEARCH COMMUNICATIONS	64	6.413
CELLULAR PHYSIOLOGY AND BIOCHEMISTRY	37	3.707
ONCOTARGET	36	3.607
SCIENTIFIC REPORTS	35	3.507
ADVANCES IN EXPERIMENTAL MEDICINE AND BIOLOGY	28	2.806
CIRCULAR RNAS BIOGENESIS AND FUNCTIONS	27	2.705
RNA BIOLOGY	23	2.305
PLOS ONE	20	2.004
NUCLEIC ACIDS RESEARCH	16	1.603
BIOMEDICINE PHARMACOTHERAPY	15	1.503
MOLECULAR CANCER	14	1.403
CANCER LETTERS	13	1.303
GENE	13	1.303
INTERNATIONAL JOURNAL OF CLINICAL AND EXPERIMENTAL PATHOLOGY	12	1.202
BMC GENOMICS	11	1.102
EPIGENOMICS	11	1.102
EUROPEAN REVIEW FOR MEDICAL AND PHARMACOLOGICAL SCIENCES	11	1.102
ONCOLOGY LETTERS	11	1.102
AGING US	10	1.002
MOLECULAR THERAPY NUCLEIC ACIDS	10	1.002
GENOME BIOLOGY	9	0.902
MEDICAL SCIENCE MONITOR	9	0.902
MOLECULAR CELL	9	0.902
MOLECULAR MEDICINE REPORTS	9	0.902
ONCOTARGETS AND THERAPY	9	0.902
AMERICAN JOURNAL OF TRANSLATIONAL RESEARCH	8	0.802
AMERICAN JOURNAL OF CANCER RESEARCH	7	0.701
BIOINFORMATICS	7	0.701
BIOMED RESEARCH INTERNATIONAL	7	0.701
CANCER MANAGEMENT AND RESEARCH	7	0.701
CELL CYCLE	7	0.701
CELL DEATH DISEASE	7	0.701
INTERNATIONAL JOURNAL OF MOLECULAR MEDICINE	7	0.701
INTERNATIONAL JOURNAL OF MOLECULAR SCIENCES	7	0.701

Table S3: Continued

**Source titles**	**Record count**	**% of 998**
JOURNAL OF CLINICAL LABORATORY ANALYSIS	7	0.701
CANCER BIOMARKERS	6	0.601
INTERNATIONAL JOURNAL OF ONCOLOGY	6	0.601
NATURE COMMUNICATIONS	6	0.601
ONCOLOGY REPORTS	6	0.601
RNA	6	0.601
THERANOSTICS	6	0.601
TUMOR BIOLOGY	6	0.601
BRIEFINGS IN BIOINFORMATICS	5	0.501
FRONTIERS IN PLANT SCIENCE	5	0.501
GENES	5	0.501
JOURNAL OF CANCER	5	0.501
JOURNAL OF CELLULAR BIOCHEMISTRY	5	0.501
JOURNAL OF EXPERIMENTAL CLINICAL CANCER RESEARCH	5	0.501
JOURNAL OF TRANSLATIONAL MEDICINE	5	0.501
ONCOGENE	5	0.501
CANCER MEDICINE	4	0.401
CANCER RESEARCH	4	0.401
CELL DEATH AND DIFFERENTIATION	4	0.401
CELL REPORTS	4	0.401
CHEMICAL COMMUNICATIONS	4	0.401
CLINICA CHIMICA ACTA	4	0.401
COMPUTATIONAL BIOLOGY AND CHEMISTRY	4	0.401
DISEASE MARKERS	4	0.401
FRONTIERS IN GENETICS	4	0.401
INTERNATIONAL JOURNAL OF BIOLOGICAL SCIENCES	4	0.401
JOURNAL OF HEMATOLOGY ONCOLOGY	4	0.401
JOURNAL OF MARINE SCIENCE AND ENGINEERING	4	0.401
JOURNAL OF THEORETICAL BIOLOGY	4	0.401
PEERJ	4	0.401
PROCEEDINGS OF THE NATIONAL ACADEMY OF SCIENCES OF THE UNITED STATES O	4	0.401
AUTOPHAGY	3	0.301
BIOSCIENCE REPORTS	3	0.301
CELL	3	0.301

Table S3: Continued

**Source titles**	**Record count**	**% of 998**
DNA AND CELL BIOLOGY	3	0.301
EBIOMEDICINE	3	0.301
EXPERIMENTAL CELL RESEARCH	3	0.301
FEBS JOURNAL	3	0.301
FEBS LETTERS	3	0.301
FRONTIERS IN IMMUNOLOGY	3	0.301
FRONTIERS IN MOLECULAR NEUROSCIENCE	3	0.301
FRONTIERS IN ONCOLOGY	3	0.301
GENES DEVELOPMENT	3	0.301
INTERNATIONAL JOURNAL OF CLINICAL AND EXPERIMENTAL MEDICINE	3	0.301
JAIDS JOURNAL OF ACQUIRED IMMUNE DEFICIENCY SYNDROMES	3	0.301
JOURNAL OF MOLECULAR AND CELLULAR CARDIOLOGY	3	0.301
MEDICINE	3	0.301
MOLECULAR IMMUNOLOGY	3	0.301
MOLECULAR NEUROBIOLOGY	3	0.301
STEM CELL RESEARCH THERAPY	3	0.301
TRANSLATIONAL CANCER RESEARCH	3	0.301
WILEY INTERDISCIPLINARY REVIEWS RNA	3	0.301
WORLD JOURNAL OF GASTROENTEROLOGY	3	0.301
ASIAN AUSTRALASIAN JOURNAL OF ANIMAL SCIENCES	2	0.2
BIOCHIMICA ET BIOPHYSICA ACTA GENE REGULATORY MECHANISMS	2	0.2
BIOMARKERS IN MEDICINE	2	0.2
BMC BIOINFORMATICS	2	0.2
BMC CANCER	2	0.2
BRIEFINGS IN FUNCTIONAL GENOMICS	2	0.2
CELL BIOLOGY INTERNATIONAL	2	0.2
CLINICAL CANCER RESEARCH	2	0.2
CURRENT GENOMICS	2	0.2
DATABASE THE JOURNAL OF BIOLOGICAL DATABASES AND CURATION	2	0.2
DNA RESEARCH	2	0.2
EUROPEAN HEART JOURNAL	2	0.2
EXPERIMENTAL BIOLOGY AND MEDICINE	2	0.2
FEBS OPEN BIO	2	0.2

Table S3: Continued

Source titles	Record count	% of 998
FRONTIERS IN PHYSIOLOGY	2	0.2
GENOMICS	2	0.2
GENOMICS PROTEOMICS BIOINFORMATICS	2	0.2
HEPATOLOGY	2	0.2
INTERNATIONAL JOURNAL OF GENOMICS	2	0.2
JOURNAL OF BIOTECHNOLOGY	2	0.2
JOURNAL OF CANCER RESEARCH AND CLINICAL ONCOLOGY	2	0.2
JOURNAL OF CELLULAR AND MOLECULAR MEDICINE	2	0.2
JOURNAL OF CELLULAR PHYSIOLOGY	2	0.2
JOURNAL OF GENERAL VIROLOGY	2	0.2
JOURNAL OF MEDICAL GENETICS	2	0.2
JOURNAL OF MOLECULAR MEDICINE JMM	2	0.2
JOVE JOURNAL OF VISUALIZED EXPERIMENTS	2	0.2
METHODS IN ENZYMOLOGY	2	0.2
MOLECULAR AND CELLULAR BIOLOGY	2	0.2
MOLECULAR THERAPY	2	0.2
NEW PHYTOLOGIST	2	0.2
OCEAN MODELLING	2	0.2
PLANTA	2	0.2
PLOS GENETICS	2	0.2
PLOS PATHOGENS	2	0.2
RESUSCITATION	2	0.2
RNA A PUBLICATION OF THE RNA SOCIETY	2	0.2
RUSSIAN JOURNAL OF BIOORGANIC CHEMISTRY	2	0.2
SCIENCE	2	0.2
TRENDS IN GENETICS	2	0.2
ACTA BIOCHIMICA POLONICA	1	0.1
ACTA DIABETOLOGICA	1	0.1
ACTA PHARMACOLOGICA SINICA	1	0.1
ADVANCES IN APPLIED MATHEMATICS	1	0.1
AGING AND AGING RELATED DISEASES MECHANISMS AND INTERVENTIONS	1	0.1
ANATOLIAN JOURNAL OF CARDIOLOGY	1	0.1
ANGEWANDTE CHEMIE INTERNATIONAL EDITION	1	0.1

Table S3: Continued

Source titles	Record count	% of 998
ARCHIVES OF BIOCHEMISTRY AND BIOPHYSICS	1	0.1
ARCHIVES OF BIOLOGICAL SCIENCES	1	0.1
ARTIFICIAL LIFE	1	0.1
BIOCHIMICA ET BIOPHYSICA ACTA MOLECULAR BASIS OF DISEASE	1	0.1
BIOCHIMICA ET BIOPHYSICA ACTA MOLECULAR CELL RESEARCH	1	0.1
BIOCHIMICA ET BIOPHYSICA ACTA REVIEWS ON CANCER	1	0.1
BIOLOGICAL CHEMISTRY	1	0.1
BIOLOGICAL RESEARCH	1	0.1
BIOLOGY OF REPRODUCTION	1	0.1
BIOSYSTEMS	1	0.1
BIOTECHNOLOGY AND BIOENGINEERING	1	0.1
BIOTECHNOLOGY BIOTECHNOLOGICAL EQUIPMENT	1	0.1
BJOG AN INTERNATIONAL JOURNAL OF OBSTETRICS AND GYNAECOLOGY	1	0.1
BLOOD	1	0.1
BLOOD CANCER JOURNAL	1	0.1
BMC BIOLOGY	1	0.1
BMC GENETICS	1	0.1
BMC PLANT BIOLOGY	1	0.1
BMC SYSTEMS BIOLOGY	1	0.1
BRAIN AND BEHAVIOR	1	0.1
BRAZILIAN JOURNAL OF MEDICAL AND BIOLOGICAL RESEARCH	1	0.1
BREAST CANCER	1	0.1
BRITISH JOURNAL OF CANCER	1	0.1
BULLETIN OF EXPERIMENTAL BIOLOGY AND MEDICINE	1	0.1
BULLETIN OF THE AMERICAN METEOROLOGICAL SOCIETY	1	0.1
CALCIFIED TISSUE INTERNATIONAL	1	0.1
CANCER BIOLOGY THERAPY	1	0.1
CANCER CELL INTERNATIONAL	1	0.1
CANCER RESEARCH AND TREATMENT	1	0.1
CANCERS	1	0.1
CARCINOGENESIS	1	0.1
CARDIOVASCULAR RESEARCH	1	0.1
CELL AND BIOSCIENCE	1	0.1

Table S3: Continued

**Source titles**	**Record count**	**% of 998**
CELL RESEARCH	1	0.1
CELLS	1	0.1
CELLULAR AND MOLECULAR BIOLOGY	1	0.1
CELLULAR AND MOLECULAR LIFE SCIENCES	1	0.1
CHEMOSPHERE	1	0.1
CHINESE JOURNAL OF CANCER RESEARCH	1	0.1
CHINESE MEDICAL JOURNAL	1	0.1
CIRCULATION	1	0.1
CLINICAL AND EXPERIMENTAL HYPERTENSION	1	0.1
CLINICAL AND EXPERIMENTAL IMMUNOLOGY	1	0.1
CLINICAL CHEMISTRY	1	0.1
CLINICAL CHEMISTRY AND LABORATORY MEDICINE	1	0.1
CLINICAL EPIGENETICS	1	0.1
CLINICAL LABORATORY	1	0.1
CLINICAL NEUROLOGY AND NEUROSURGERY	1	0.1
CLINICAL SCIENCE	1	0.1
CLINICAL TRANSLATIONAL ONCOLOGY	1	0.1
COASTAL ENGINEERING	1	0.1
COMPUTATIONAL AND STRUCTURAL BIOTECHNOLOGY JOURNAL	1	0.1
CRITICAL REVIEWS IN BIOCHEMISTRY AND MOLECULAR BIOLOGY	1	0.1
CURRENT MICROBIOLOGY	1	0.1
CURRENT OPINION IN GENETICS DEVELOPMENT	1	0.1
DEVELOPMENT	1	0.1
DIABETES VASCULAR DISEASE RESEARCH	1	0.1
DISCRETE MATHEMATICS	1	0.1
ELIFE	1	0.1
EMBO JOURNAL	1	0.1
ENVIRONMENTAL FLUID MECHANICS	1	0.1
ENVIRONMENTAL HEALTH AND PREVENTIVE MEDICINE	1	0.1
EUROPEAN JOURNAL OF HEART FAILURE	1	0.1
EXPERIMENTAL AND MOLECULAR MEDICINE	1	0.1
EXPERIMENTAL AND THERAPEUTIC MEDICINE	1	0.1
EXPERIMENTAL DERMATOLOGY	1	0.1

Table S3: Continued

Source titles	Record count	% of 998
EXPERT OPINION ON THERAPEUTIC PATENTS	1	0.1
FASEB JOURNAL	1	0.1
FISH SHELLFISH IMMUNOLOGY	1	0.1
FORENSIC SCIENCE MEDICINE AND PATHOLOGY	1	0.1
FRONTIERS IN CELL AND DEVELOPMENTAL BIOLOGY	1	0.1
FRONTIERS IN CELLULAR NEUROSCIENCE	1	0.1
FRONTIERS IN MOLECULAR BIOSCIENCES	1	0.1
FRONTIERS IN PHARMACOLOGY	1	0.1
GENES GENOMICS	1	0.1
GENOME RESEARCH	1	0.1
GIGASCIENCE	1	0.1
HAEMATOLOGICA	1	0.1
HEPATOLOGY RESEARCH	1	0.1
HISTOLOGY AND HISTOPATHOLOGY	1	0.1
HUMAN CELL	1	0.1
HUMAN MOLECULAR GENETICS	1	0.1
IMMUNITY	1	0.1
IMMUNITY AGEING	1	0.1
IMMUNOLOGY	1	0.1
INSECT BIOCHEMISTRY AND MOLECULAR BIOLOGY	1	0.1
INTERNATIONAL BRAZ J UROL	1	0.1
INTERNATIONAL JOURNAL OF APPLIED EARTH OBSERVATION AND GEOINFORMATION	1	0.1
INTERNATIONAL JOURNAL OF BIOCHEMISTRY CELL BIOLOGY	1	0.1
INTERNATIONAL JOURNAL OF HYDROGEN ENERGY	1	0.1
INTERNATIONAL JOURNAL OF LEGAL MEDICINE	1	0.1
INTERNATIONAL JOURNAL OF MEDICAL SCIENCES	1	0.1
INTERNATIONAL JOURNAL OF NEUROSCIENCE	1	0.1
INVESTIGATIVE OPHTHALMOLOGY VISUAL SCIENCE	1	0.1
IRANIAN JOURNAL OF BASIC MEDICAL SCIENCES	1	0.1
JNCI JOURNAL OF THE NATIONAL CANCER INSTITUTE	1	0.1
JOURNAL OF ANIMAL SCIENCE AND BIOTECHNOLOGY	1	0.1
JOURNAL OF BIOSCIENCE AND BIOENGINEERING	1	0.1
JOURNAL OF BREAST CANCER	1	0.1

Table S3: Continued

Source titles	Record count	% of 998
JOURNAL OF CLINICAL MICROBIOLOGY	1	0.1
JOURNAL OF COASTAL RESEARCH	1	0.1
JOURNAL OF DAIRY SCIENCE	1	0.1
JOURNAL OF DERMATOLOGICAL SCIENCE	1	0.1
JOURNAL OF EXTRACELLULAR VESICLES	1	0.1
JOURNAL OF FLOOD RISK MANAGEMENT	1	0.1
JOURNAL OF HEPATOLOGY	1	0.1
JOURNAL OF HUAZHONG UNIVERSITY OF SCIENCE AND TECHNOLOGY MEDICAL SCIEN	1	0.1
JOURNAL OF HYDRAULIC ENGINEERING	1	0.1
JOURNAL OF HYDRAULIC ENGINEERING ASCE	1	0.1
JOURNAL OF IMMUNOLOGY RESEARCH	1	0.1
JOURNAL OF MARINE SYSTEMS	1	0.1
JOURNAL OF MOLECULAR BIOLOGY	1	0.1
JOURNAL OF MOLECULAR NEUROSCIENCE	1	0.1
JOURNAL OF NEURO ONCOLOGY	1	0.1
JOURNAL OF NEUROCHEMISTRY	1	0.1
JOURNAL OF NEUROSCIENCE	1	0.1
JOURNAL OF NEUROTRAUMA	1	0.1
JOURNAL OF OVARIAN RESEARCH	1	0.1
JOURNAL OF PAIN RESEARCH	1	0.1
JOURNAL OF PATHOLOGY	1	0.1
JOURNAL OF PERIODONTOLOGY	1	0.1
JOURNAL OF PHYSICAL CHEMISTRY A	1	0.1
JOURNAL OF PHYSIOLOGY AND BIOCHEMISTRY	1	0.1
JOURNAL OF PROTEOME RESEARCH	1	0.1
JOURNAL OF SCIENTIFIC COMPUTING	1	0.1
JOURNAL OF STATISTICAL SOFTWARE	1	0.1
JOURNAL OF THE AMERICAN CHEMICAL SOCIETY	1	0.1
JOURNAL OF THE AMERICAN HEART ASSOCIATION	1	0.1
JOURNAL OF THE EUROPEAN ACADEMY OF DERMATOLOGY AND VENEREOLOGY	1	0.1
JOURNAL OF THE NEUROLOGICAL SCIENCES	1	0.1
JOURNAL OF THORACIC DISEASE	1	0.1

Table S3: Continued

Source titles	Record count	% of 998
LABORATORY INVESTIGATION	1	0.1
LEUKEMIA RESEARCH	1	0.1
MARINE GENOMICS	1	0.1
MECHANISMS OF AGEING AND DEVELOPMENT	1	0.1
METABOLISM CLINICAL AND EXPERIMENTAL	1	0.1
MOLECULAR BIOLOGY	1	0.1
MOLECULAR BIOSYSTEMS	1	0.1
MOLECULAR CANCER RESEARCH	1	0.1
MOLECULAR CARCINOGENESIS	1	0.1
MOLECULAR MEDICINE	1	0.1
MOLECULAR METABOLISM	1	0.1
MOLECULAR ONCOLOGY	1	0.1
MOLECULAR VISION	1	0.1
MOLECULES	1	0.1
MONTHLY WEATHER REVIEW	1	0.1
NATURE	1	0.1
NATURE BIOTECHNOLOGY	1	0.1
NATURE NEUROSCIENCE	1	0.1
NATURE PLANTS	1	0.1
NATURE REVIEWS GENETICS	1	0.1
NATURE REVIEWS MOLECULAR CELL BIOLOGY	1	0.1
NATURE STRUCTURAL MOLECULAR BIOLOGY	1	0.1
NEUROGENETICS	1	0.1
NEUROSCIENCE LETTERS	1	0.1
NPJ GENOMIC MEDICINE	1	0.1
OCEAN DYNAMICS	1	0.1
OLIGONUCLEOTIDES	1	0.1
OPHTHALMIC RESEARCH	1	0.1
OXIDATIVE MEDICINE AND CELLULAR LONGEVITY	1	0.1

Table S3: Continued

Source titles	Record count	% of 998
PHARMACOLOGY THERAPEUTICS	1	0.1
PHYSIOLOGIA PLANTARUM	1	0.1
PHYSIOLOGICAL GENOMICS	1	0.1
PHYTOCHEMICAL ANALYSIS	1	0.1
PLANT MOLECULAR BIOLOGY	1	0.1
PLANT SCIENCE	1	0.1
PLOS COMPUTATIONAL BIOLOGY	1	0.1
POSTHARVEST BIOLOGY AND TECHNOLOGY	1	0.1
POWER	1	0.1
REPRODUCTION FERTILITY AND DEVELOPMENT	1	0.1
REPRODUCTIVE SCIENCES	1	0.1
RESPIRATORY RESEARCH	1	0.1
RIBOSWITCH DISCOVERY STRUCTURE AND FUNCTION	1	0.1
ROYAL SOCIETY OPEN SCIENCE	1	0.1
SAMJ SOUTH AFRICAN MEDICAL JOURNAL	1	0.1
SCANDINAVIAN JOURNAL OF GASTROENTEROLOGY	1	0.1
SCIENCE CHINA LIFE SCIENCES	1	0.1
SENSORS AND MATERIALS	1	0.1
STROKE	1	0.1
THORACIC CANCER	1	0.1
TRANSLATIONAL ONCOLOGY	1	0.1
TRENDS IN NEUROSCIENCES	1	0.1
VIRUSES BASEL	1	0.1
VISUALIZING RNA DYNAMICS IN THE CELL	1	0.1
WORLD JOURNAL OF GASTROINTESTINAL ONCOLOGY	1	0.1
WORLD JOURNAL OF SURGICAL ONCOLOGY	1	0.1
WORLD NEUROSURGERY	1	0.1
YONSEI MEDICAL JOURNAL	1	0.1

Table S4: All funding agencies that published research related to circular RNA

Funding agency	Record count	% of 998
NATIONAL NATURAL SCIENCE FOUNDATION OF CHINA	455	45.5
NATIONAL INSTITUTES OF HEALTH NIH USA	67	6.7
UNITED STATES DEPARTMENT OF HEALTH HUMAN SERVICES	67	6.7
NATURAL SCIENCE FOUNDATION OF JIANGSU PROVINCE	31	3.1
FUNDAMENTAL RESEARCH FUNDS FOR THE CENTRAL UNIVERSITIES	30	3
NATIONAL BASIC RESEARCH PROGRAM OF CHINA	30	3
CHINA POSTDOCTORAL SCIENCE FOUNDATION	26	2.6
GERMAN RESEARCH FOUNDATION DFG	21	2.1
NATIONAL NATURAL SCIENCE FOUNDATION OF GUANGDONG PROVINCE	21	2.1
CHINESE ACADEMY OF SCIENCES	18	1.8
NATIONAL KEY RESEARCH AND DEVELOPMENT PROGRAM OF CHINA	18	1.8
JIANGSU PLANNED PROJECTS FOR POSTDOCTORAL RESEARCH FUNDS	16	1.6
NATURAL SCIENCE FOUNDATION OF ZHEJIANG PROVINCE	15	1.5
K C WONG MAGNA FUND IN NINGBO UNIVERSITY	13	1.3
NATIONAL SCIENCE FOUNDATION NSF	13	1.3
APPLIED RESEARCH PROJECT ON NONPROFIT TECHNOLOGY OF ZHEJIANG PR	12	1.2
BEIJING NATURAL SCIENCE FOUNDATION	12	1.2
NIH NATIONAL CANCER INSTITUTE NCI	12	1.2
EUROPEAN RESEARCH COUNCIL ERC	11	1.1
MINISTRY OF SCIENCE AND TECHNOLOGY CHINA	11	1.1
PRIORITY ACADEMIC PROGRAM DEVELOPMENT OF JIANGSU HIGHER EDUCATI	11	1.1
CANADIAN INSTITUTES OF HEALTH RESEARCH CIHR	10	1
NATIONAL KEY R D PROGRAM OF CHINA	10	1
SCIENTIFIC INNOVATION TEAM PROJECT OF NINGBO	10	1
CHINA SCHOLARSHIP COUNCIL		0.9
EUROPEAN UNION EU		0.9
NATURAL SCIENCE FOUNDATION OF SHANDONG PROVINCE		0.9
NIH NATIONAL INSTITUTE OF GENERAL MEDICAL SCIENCES NIGMS		0.9
LUNDBECKFONDEN		0.8
MINISTRY OF EDUCATION CULTURE SPORTS SCIENCE AND TECHNOLOGY JAP		0.8
NATIONAL HIGH TECHNOLOGY RESEARCH AND DEVELOPMENT PROGRAM OF CH		0.8
DET FRIE FORSKNINGSRAD DFF		0.7
HEART STROKE FOUNDATION OF ONTARIO		0.7
NATURAL SCIENCE FOUNDATION OF HUNAN PROVINCE		0.7
NOVO NORDISK		0.7
BREAST CANCER FOUNDATION OF ONTARIO		0.6
FEDERAL MINISTRY OF EDUCATION RESEARCH BMBF		0.6
HOWARD HUGHES MEDICAL INSTITUTE		0.6
NATURAL SCIENCE FOUNDATION OF NINGBO		0.6

Table S4: Continued

Funding agency	Record count	% of 998
NATURAL SCIENCES AND ENGINEERING RESEARCH COUNCIL OF CANADA		0.6
NIH NATIONAL INSTITUTE ON AGING NIA		0.6
PROVINCE NATURAL SCIENCE FOUNDATION OF JIANGXI PROVINCE		0.6
SCIENCE AND TECHNOLOGY PROGRAM OF GUANGZHOU		0.6
SCIENCE TECHNOLOGY COMMISSION OF SHANGHAI MUNICIPALITY STCSM		0.6
ZHEJIANG PROVINCIAL HIGH EDUCATION TEACHING REFORM PROJECT		0.6
FOUNDATION FOR DISTINGUISHED YOUNG SCIENTISTS OF JIANGXI PROVIN		0.5
MINISTRY OF SCIENCE AND TECHNOLOGY TAIWAN		0.5
NATIONAL HEALTH AND MEDICAL RESEARCH COUNCIL OF AUSTRALIA		0.5
NATIONAL KEY TECHNOLOGY R D PROGRAM		0.5
NATURAL SCIENCE FOUNDATION OF FUJIAN PROVINCE		0.5
NATURAL SCIENCE FOUNDATION OF HUBEI PROVINCE		0.5
PRIORITY ACADEMIC PROGRAM DEVELOPMENT OF JIANGSU HIGHER EDUCATI		0.5
SHANGHAI PUJIANG PROGRAM		0.5
SHANGHAI YOUTH TALENT SUPPORT PROGRAM	5	0.5
SPECIALIZED RESEARCH FUND FOR THE DOCTORAL PROGRAM OF HIGHER ED	5	0.5
ZHEJIANG MEDICAL SCIENTIFIC RESEARCH FOUNDATION	5	0.5
BURROUGHS WELLCOME FUND	4	0.4
CAMS INNOVATION FUND FOR MEDICAL SCIENCES	4	0.4
CHINA AGRICULTURE RESEARCH SYSTEM	4	0.4
DEFENCE RESEARCH DEVELOPMENT ORGANISATION DRDO	4	0.4
DEVELOPMENT OF MEDICAL SCIENCE AND TECHNOLOGY FOUNDATION OF NAN	4	0.4
FOUNDATION FOR HEPATITIS PREVENTION AND CONTROL PROJECT OF CHIN	4	0.4
FOUNDATION OF SCIENTIFIC TECHNICAL RESEARCH PROJECT OF JIANGXI	4	0.4
FOUNDATION OF ZHEJIANG KEY LABORATORY OF PATHOPHYSIOLOGY	4	0.4
JIANGSU COLLABORATIVE INNOVATION CENTER FOR MODERN CROP PRODUCT	4	0.4
MINISTERIO DE ECONOMIA Y COMPETITIVIDAD OF SPAIN	4	0.4
MINISTERO DELL ISTRUZIONE DELL UNIVERSITA E DELLA RICERCA MIUR	4	0.4
MINISTRY OF SCIENCE AND TECHNOLOGY MOST KOREA	4	0.4
NATIONAL HEALTH RESEARCH INSTITUTES TAIWAN	4	0.4
NATURAL SCIENCE FOUNDATION OF HEILONGJIANG PROVINCE	4	0.4
NATURAL SCIENCE FOUNDATION OF LIAONING PROVINCE	4	0.4
SCIENCE AND TECHNOLOGY PLANNING PROJECT OF GUANGDONG PROVINCE	4	0.4
SHANGHAI MUNICIPAL COMMISSION OF HEALTH AND FAMILY PLANNING	4	0.4
SHANGHAI SAILING PROGRAM	4	0.4
SOCIAL DEVELOPMENT RESEARCH PROJECT OF NINGBO	4	0.4
STATE KEY DEVELOPMENT PROGRAM FOR BASIC RESEARCH OF CHINA	4	0.4
UNIVERSITY OF PENNSYLVANIA	4	0.4
ACADEMIA SINICA TAIWAN	3	0.3
ADMINISTRATION OF TRADITIONAL CHINESE MEDICINE OF GUANGDONG PRO	3	0.3

Table S4: Continued

Funding agency	Record count	% of 998
BASIC RESEARCH PROGRAM OF SHENZHEN INNOVATION COUNCIL OF CHINA	3	0.3
CANCER PREVENTION RESEARCH INSTITUTE OF TEXAS	3	0.3
CARLSBERG FOUNDATION	3	0.3
CENTRE NATIONAL DE LA RECHERCHE SCIENTIFIQUE CNRS	3	0.3
FRENCH NATIONAL RESEARCH AGENCY ANR	3	0.3
GUANGDONG PROVINCE	3	0.3
GUANGDONG PROVINCIAL SCIENCE AND TECHNOLOGY KEY PROJECT	3	0.3
GUANGDONG SCIENCE AND TECHNOLOGY DEVELOPMENT FUND	3	0.3
HEALTH AND FAMILY PLANNING COMMISSION RESEARCH PROJECT OF HEILO	3	0.3
HEALTH PUBLIC WELFARE INDUSTRY SPECIAL SCIENTIFIC RESEARCH PROJ	3	0.3
INNOVATION CAPABILITY DEVELOPMENT PROJECT OF JIANGSU PROVINCE	3	0.3
JAPAN SOCIETY FOR THE PROMOTION OF SCIENCE	3	0.3
KEY CLINICAL SPECIALTY DISCIPLINE CONSTRUCTION PROGRAM	3	0.3
MAX PLANCK SOCIETY	3	0.3
MEDICAL AND HEALTH RESEARCH PROJECT OF ZHEJIANG PROVINCE	3	0.3
MEDICAL RESEARCH PROJECT OF THE AFFILIATED HOSPITAL OF NINGBO U	3	0.3
MINISTRY OF EDUCATION CHINA	3	0.3
MINISTRY OF EDUCATION TAIWAN	3	0.3
NANJING MEDICAL SCIENCE AND TECHNOLOGY DEVELOPMENT FOUNDATION	3	0.3
NANJING MEDICAL SCIENCE AND TECHNOLOGY DEVELOPMENT PROJECT	3	0.3
NATIONAL HEALTH AND FAMILY PLANNING COMMISSION OF CHINA	3	0.3
NATIONAL INSTITUTE ON AGING INTRAMURAL RESEARCH PROGRAM OF THE	3	0.3
NATIONAL KEY CLINICAL SPECIALIST CONSTRUCTION PROGRAMS OF CHINA	3	0.3
NATURAL SCIENCE FOUNDATION OF ANHUI PROVINCE	3	0.3
NATURAL SCIENCE FOUNDATION OF JILIN PROVINCE	3	0.3
NATURAL SCIENCE FOUNDATION OF SHANGHAI	3	0.3
NATURAL SCIENCE FOUNDATION OF TIANJIN	3	0.3
NETHERLANDS ORGANIZATION FOR SCIENTIFIC RESEARCH NWO	3	0.3
PROVINCIAL INITIATIVE PROGRAM FOR EXCELLENCY DISCIPLINES OF JIA	3	0.3
REGIONAL MEDICAL CENTER PROJECT OF ZHEJIANG PROVINCE	3	0.3
RESEARCH FUND OF PUBLIC WELFARE IN THE HEALTH INDUSTRY	3	0.3
RUSSIAN ACADEMY OF SCIENCES	3	0.3
RUSSIAN FOUNDATION FOR BASIC RESEARCH RFBR	3	0.3
RUSSIAN SCIENCE FOUNDATION RSF	3	0.3
SCIENTIFIC RESEARCH FOUNDATION FOR THE RETURNED OVERSEAS CHINES	3	0.3
SHANGHAI JIAO TONG UNIVERSITY MEDICINE ENGINEERING FUND	3	0.3
SHANGHAI JIAOTONG UNIVERSITY MEDICAL ENGINEERING CROSS RESEARCH	3	0.3
SHANGHAI PUJIANG PROGRAM OF SHANGHAI SCIENCE AND TECHNOLOGY COM	3	0.3

Table S4: Continued

Funding agency	Record count	% of 998
SIMUNYE PRIMARY HEALTH CARE	3	0.3
SIX TALENT PEAK PROJECT OF JIANGSU PROVINCE	3	0.3
AGENCY FOR SCIENCE TECHNOLOGY RESEARCH ASTAR	2	0.2
AGRICULTURAL SCIENCE AND TECHNOLOGY INNOVATION PROGRAM	2	0.2
ALFRED P SLOAN FOUNDATION	2	0.2
AMERICAN HEART ASSOCIATION	2	0.2
APPLIED BASIC RESEARCH PROGRAMS OF QINGDAO CHINA	2	0.2
BAXTER FAMILY FELLOWSHIP	2	0.2
BEIJING MUNICIPAL ADMINISTRATION OF HOSPITALS ASCENT PLAN	2	0.2
BEIJING MUNICIPAL ADMINISTRATION OF HOSPITALS YOUTH PROGRAMME	2	0.2
BEIJING MUNICIPAL SCIENCE TECHNOLOGY COMMISSION	2	0.2
BIH	2	0.2
BILL AND MELINDA GATES FOUNDATION BMGF THROUGH POPULATION SERVI	2	0.2
CANARY FOUNDATION	2	0.2
CAPITAL SCIENCE AND TECHNOLOGY DEVELOPMENT FUND	2	0.2
CHINA AGRICULTURE RESEARCH SYSTEM PROJECT	2	0.2
COUNCIL OF SCIENTIFIC INDUSTRIAL RESEARCH CSIR INDIA	2	0.2
DAMON RUNYON CANCER RESEARCH FOUNDATION	2	0.2
DEPARTMENT OF THORACIC SURGERY AFFILIATED HOSPITAL OF QINGDAO U	2	0.2
DEVELOPMENT FUND FOR SHANGHAI TALENTS	2	0.2
DFG GRADUATE SCHOOL COMPUTATIONAL SYSTEMS BIOLOGY	2	0.2
DZHK GERMAN CENTRE FOR CARDIOVASCULAR RESEARCH	2	0.2
EARMARKED FUND FOR MODERN AGRO INDUSTRY TECHNOLOGY RESEARCH SYS	2	0.2
ENGINEERING PHYSICAL SCIENCES RESEARCH COUNCIL EPSRC	2	0.2
EPIGEN EPIGENOMICS FLAGSHIP PROJECT	2	0.2
EUROPEAN COMMISSION JOINT RESEARCH CENTRE	2	0.2
EYE ENT HOSPITAL	2	0.2
FONDAZIONE ROMA	2	0.2
FONDAZIONE TELETHON	2	0.2
FOUNDATION FOR HIGH LEVEL TALENTS IN HIGHER EDUCATION IN GUANGD	2	0.2
GERMAN CENTER FOR CARDIOVASCULAR RESEARCH DZHK	2	0.2
GERMAN ISRAELI FOUNDATION FOR SCIENTIFIC RESEARCH AND DEVELOPME	2	0.2
GUANGZHOU HEALTH AND FAMILY PLANNING COMMISSION PROGRAM	2	0.2
HEED OPHTHALMIC FOUNDATION	2	0.2
HEILONGJIANG POSTDOCTORAL SCIENCE FOUNDATION	2	0.2
HUMAN FRONTIERS SCIENCE PROGRAM AWARD	2	0.2
IFB TX	2	0.2
INNOVATION PROGRAM OF SHANGHAI MUNICIPAL EDUCATION COMMISSION	2	0.2
INNOVATIVE SCIENCE FOUNDATION OF HARBIN MEDICAL UNIVERSITY	2	0.2

Table S4: Continued

Funding agency	Record Count	% of 998
JAPAN SCIENCE TECHNOLOGY AGENCY JST	2	0.2
JIANGSU OVERSEAS RESEARCH AND TRAINING PROGRAM FOR UNIVERSITY P	2	0.2
JIANGSU PROVINCE S KEY PROVINCIAL TALENTS PROGRAM	2	0.2
JIANGSU PROVINCIAL MEDICAL YOUTH TALENT	2	0.2
JIANGXI KEY RESEARCH AND DEVELOPMENT PROGRAM	2	0.2
KEY RESEARCH PROGRAM FOR THE SOCIAL DEVELOPMENT OF JIANGSU PROV	2	0.2
LEDUCQ FOUNDATION CADGENOMICS	2	0.2
LIAONING BAIQIANWAN TALENTS PROGRAM	2	0.2
LIAONING CLINICAL RESEARCH CENTER FOR COLORECTAL CANCER	2	0.2
LIAONING SCIENCE AND TECHNOLOGY PLAN PROJECT	2	0.2
LOEWE PROGRAM MEDICAL RNOMICS STATE OF HESSEN	2	0.2
MAJOR PROGRAM OF NATURAL SCIENCE FOUNDATION OF JIANGSU PROVINCE	2	0.2
MEDICAL AND HEALTH RESEARCH PROGRAM OF ZHEJIANG PROVINCE	2	0.2
MEDICAL AND HEALTH SCIENCE AND TECHNOLOGY PROJECT OF PANYU DIST	2	0.2
MEDICAL RESEARCH COUNCIL UK MRC	2	0.2
MEDICAL TRAINING PROGRAMME FOUNDATION FOR THE TALENTS BY JIANGS	2	0.2
MINISTRY OF EDUCATION AND SCIENCE SPAIN	2	0.2
MINISTRY OF EDUCATION CHINA 111 PROJECT	2	0.2
MINISTRY OF HEALTH ITALY	2	0.2
MINISTRY OF SCIENCE AND TECHNOLOGY MOST TAIWAN	2	0.2
NATIONAL KEY RD PROGRAM	2	0.2
NATIONAL KEY RESEARCH AND DEVELOPMENT PROGRAM	2	0.2
NATIONAL KEY RESEARCH AND DEVELOPMENT PROGRAM OF THE MINISTRY O	2	0.2
NATIONAL NATURAL SCIENCE FOUNDATION OF GUANGXI PROVINCE	2	0.2
NATIONAL PROGRAM ON KEY RESEARCH PROJECT	2	0.2
NATIONAL SCIENCE AND TECHNOLOGY MAJOR PROJECT	2	0.2
NATIONAL SCIENCE TECHNOLOGY PILLAR PROGRAM OF CHINA	2	0.2
NIGMS GRANT	2	0.2
NIH NATIONAL HEART LUNG BLOOD INSTITUTE NHLBI	2	0.2
OPENING FOUNDATION OF JIANGSU KEY LABORATORY OF ANESTHESIOLOGY	2	0.2
ORDINARY UNIVERSITY GRADUATE STUDENT RESEARCH INNOVATION PROJEC	2	0.2
PEKING UNION MEDICAL COLLEGE YOUTH FUND	2	0.2
POSTGRADUATE INNOVATIVE RESEARCH PROJECT OF HARBIN MEDICAL UNIV	2	0.2
POSTGRADUATE RESEARCH PRACTICE INNOVATION PROGRAM OF JIANGSU PR	2	0.2
PROGRAM FOR ACADEMIC LEADERS OF SHANGHAI	2	0.2
PROGRAM FOR NEW CENTURY EXCELLENT TALENTS IN UNIVERSITY NCET	2	0.2
PROGRAM OF THE COMMITTEE OF SCIENCE AND TECHNOLOGY	2	0.2
PROJECT OF JIANGSU PROVINCIAL MEDICAL TALENT	2	0.2
REBIRTH EXCELLENCE CLUSTER	2	0.2

Table S4: Continued

Funding agency	Record count	% of 998
SCIENCE AND TECHNOLOGY DEPARTMENT OF HENAN PROVINCE	2	0.2
SCIENCE AND TECHNOLOGY INNOVATION FUND OF FUJIAN AGRICULTURE AN	2	0.2
SCIENCE AND TECHNOLOGY PLAN PROJECT OF THE EDUCATION DEPARTMENT	2	0.2
SCIENCE AND TECHNOLOGY PLANNING PROJECT OF FUJIAN PROVINCE	2	0.2
SCIENCE AND TECHNOLOGY PROJECT OF HEALTH AND FAMILY PLANNING CO	2	0.2
SCIENCE TECHNOLOGY BUREAU OF GUANGDONG PROVINCE	2	0.2
SCIENCE TECHNOLOGY DEPARTMENT OF ZHEJIANG PROVINCE	2	0.2
SCIENTIFIC RESEARCH INNOVATION PROJECT FOR GRADUATE STUDENTS IN	2	0.2
SHAANXI SCIENCE AND TECHNOLOGY INNOVATION PROJECT PLAN	2	0.2
SHANDONG POSTDOCTORAL INNOVATION SPECIAL FUND	2	0.2
SHANGHAI MUNICIPAL COMMISSION OF AGRICULTURE	2	0.2
SOCIAL DEVELOPMENT MAJOR PROJECTS OF NINGBO	2	0.2
SOCIAL DEVELOPMENT PROJECT IN JIANGSU PROVINCE	2	0.2
SPECIAL FUND FOR AGRO SCIENTIFIC RESEARCH IN THE PUBLIC INTERES	2	0.2
SUZHOU AGRICULTURAL SCIENCE AND TECHNOLOGY INNOVATION PROJECT	2	0.2
SWEDISH RESEARCH COUNCIL	2	0.2
SWISS NATIONAL SCIENCE FOUNDATION SNSF	2	0.2
TAISHAN SCHOLARS PROGRAM	2	0.2
TALENT TRAINING PROJECT OF HIGH LEVEL HEALTH TECHNOLOGY FROM BE	2	0.2
TATEMATSU FOUNDATION	2	0.2
TIANJIN PUBLIC HEALTH KEY RESEARCH PROJECT	2	0.2
TOP UNIVERSITY GRANT OF NATIONAL CHENG KUNG UNIVERSITY	2	0.2
UNITED STATES DEPARTMENT OF DEFENSE	2	0.2
UNIVERSITY OF NEVADA	2	0.2
US DEPARTMENT OF VETERAN AFFAIRS	2	0.2
VILLUM FONDEN	2	0.2
VILLUM FOUNDATION	2	0.2
WELLCOME TRUST	2	0.2
YOUNG TALENTS PROGRAM OF JIANGSU CANCER HOSPITAL	2	0.2
YOUTH INNOVATION PROMOTION ASSOCIATION CAS	2	0.2
YUNNAN APPLIED BASIC RESEARCH PROJECTS	2	0.2
ZHEJIANG PROVINCIAL MEDICINE AND HEALTH SCIENCE RESEARCH FOUNDA	2	0.2
1000 TALENT PLAN OF CHINA	1	0.1
1000 YOUNG TALENTS PLAN PROGRAM OF CHINA	1	0.1
111 PROJECT OF MOE CHINA	1	0.1
1351 PERSONNEL TRAINING PROGRAM OF BEIJING CHAO YANG HOSPITAL A	1	0.1
13TH FIVE YEAR PLAN NATIONAL SCIENCE AND TECHNOLOGY SUPPORTING	1	0.1
2013 SAIL PLAN INTRODUCTION OF THE SHORTAGE OF TOP NOTCH TALENT	1	0.1
2018 SCIENCE RESEARCH FOUNDATION IN AFFILIATED HOSPITAL OF YANG	1	0.1

Table S4: Continued

Funding agency	Record count	% of 998
333 PROJECT FOUNDATION OF JIANGSU PROVINCE	1	0.1
863 PROGRAM OF CHINA	1	0.1
AARHUS UNIVERSITY GSST PHD SCHOOL	1	0.1
ACADEMIC CLIMBING PROJECT OF ZHEJIANG PROVINCIAL UNIVERSITIES D	1	0.1
ACADEMIC EXCELLENCE ALLIANCE GRANT	1	0.1
ADVANCED HEALTH CARE PROFESSIONALS DEVELOPMENT PROJECT OF BEIJI	1	0.1
AGRICULTURAL SCIENCE AND TECHNOLOGY INDEPENDENT INNOVATION PROJ	1	0.1
AGRICULTURE MAJOR NEW VARIETY CREATION PROJECT IN JIANGSU PROVI	1	0.1
AIMING FOR THE TOP UNIVERSITY PROGRAM OF THE NATIONAL CHIAO TUN	1	0.1
ALFRED SLOAN RESEARCH FELLOWSHIP	1	0.1
AMERICAN CANCER SOCIETY	1	0.1
AMERICAN DIABETES ASSOCIATION	1	0.1
AMERICAN ITALIAN CANCER FOUNDATION	1	0.1
AMERICAN PHYSIOLOGICAL SOCIETY SHIH CHUN WANG YOUNG INVESTIGATO	1	0.1
ANDREW RACHLEFF ENDOWED FELLOWSHIP	1	0.1
ANHUI MEDICAL UNIVERSITY EARLY CONTACT RESEARCH OF CLINICAL MED	1	0.1
ANHUI PROVINCIAL EDUCATION DEPARTMENT	1	0.1
ANIMAL FREE RESEARCH	1	0.1
APPLICATION INFRASTRUCTURE PROJECTS OF SICHUAN PROVINCE	1	0.1
APPLIED BASIC RESEARCH PROGRAM OF CHANGZHOU	1	0.1
APPLIED RESEARCH PROJECT ON NON PROFIT TECHNOLOGY OF ZHEJIANG P	1	0.1
APPRECIATED THE BEAUTY OF LIFE INC	1	0.1
ARISLA ARCI	1	0.1
ARISLA FULL GRANT ARCI	1	0.1
ARIZONA DEPARTMENT OF HEALTH SERVICES	1	0.1
ARVID NILSSONS FOND	1	0.1
ASSOCIAZIONE ITALIANA PER LA RICERCA SUL CANCRO AIRC	1	0.1
ASTRAZENECA	1	0.1
ATIP AVENIR	1	0.1
AUSTRALIAN RESEARCH COUNCIL	1	0.1
BASIC AND CLINICAL COOPERATION TOPIC OF CAPITAL MEDICAL UNIVERS	1	0.1
BASIC MEDICAL RESEARCH CENTER	1	0.1
BASIC MEDICAL RESEARCH CENTER OF HUAIHE HOSPITAL OF HENAN UNIVE	1	0.1
BASIC MEDICAL RESEARCH CENTER OF LIAOCHENG PEOPLE S HOSPITAL	1	0.1
BASIC MEDICAL RESEARCH CENTER OF SHANGDONG UNIVERSITY	1	0.1
BASIC MEDICAL RESEARCH CENTER OF ZHEJIANG PROVINCIAL PEOPLE S H	1	0.1
BASIC PROJECT PROGRAM OF BASIC RESEARCH OF THE STATE ACADEMIES	1	0.1
BASIC RESEARCH FUND FOR CENTRAL PUBLIC RESEARCH INSTITUTES OF C	1	0.1
BASIC RESEARCH PROJECTS OF SHANDONG PROVINCE	1	0.1

Table S4: Continued

Funding agency	Record count	% of 998
BAVARIAN MINISTRY OF SCIENCES RESEARCH AND THE ARTS IN THE FRAM	1	0.1
BAYLOR RESEARCH INSTITUTE	1	0.1
BAYLOR SAMMONS CANCER CENTER AND FOUNDATION	1	0.1
BECA MOVILIDAD ESTUDIANTIL UNAM	1	0.1
BEIJING ACADEMY OF AGRICULTURAL AND FORESTRY SCIENCES	1	0.1
BEIJING CHINESE MEDICINE NEW AUSTRIAN AWARD FUND	1	0.1
BEIJING JOINT PROJECT FOR THE CENTRAL AFFILIATED UNIVERSITY	1	0.1
BEIJING MEDICAL AND HEALTH FOUNDATION	1	0.1
BEIJING MUNICIPAL ADMINISTRATION OF HOSPITALS	1	0.1
BEIJING MUNICIPAL ADMINISTRATION OF HOSPITALS CLINICAL MEDICINE	1	0.1
BEIJING MUNICIPAL ADMINISTRATION OF HOSPITALS MISSION PLAN	1	0.1
BEIJING MUNICIPAL COMMISSION OF EDUCATION	1	0.1
BEIJING MUNICIPAL SCIENCE AND TECHNOLOGY COMMISSION RESEARCH FU	1	0.1
BELGIAN FEDERAL SCIENCE POLICY OFFICE	1	0.1
BERLIN INSTITUTE OF HEALTH BERLIN GERMANY	1	0.1
BERLIN INSTITUTE OF HEALTH BIH	1	0.1
BERLIN SCHOOL OF INTEGRATIVE ONCOLOGY	1	0.1
BERLINER KREBSGESELLSCHAFT E V	1	0.1
BETHUNE YOUTH FOUNDATION OF JILIN UNIVERSITY CHINA	1	0.1
BIF	1	0.1
BIH CRG	1	0.1
BIOSIGNATURE RESEARCH GRANT	1	0.1
BIOTECHNOLOGY AND BIOLOGICAL SCIENCES RESEARCH COUNCIL BBSRC	1	0.1
BLADDER CANCER TRANSLATIONAL MEDICINE PROJECT OF LIAONING AND S	1	0.1
BMBF FOR THE BERLIN INSTITUTE FOR MEDICAL SYSTEMS BIOLOGY	1	0.1
BMBF PROJECT SUPR G	1	0.1
BMBF SUPPORT FOR THE DZHK	1	0.1
BMRP GRANT FROM QATAR FOUNDATION	1	0.1
BOEHRINGER INGELHEIM	1	0.1
BRITISH HEART FOUNDATION	1	0.1
CALIFORNIA CITRUS RESEARCH BOARD	1	0.1
CALIFORNIA INSTITUTE FOR REGENERATIVE MEDICINE BRIDGES TO STEM	1	0.1
CALIFORNIA INSTITUTE OF REGENERATIVE MEDICINE CIRM GRANT	1	0.1
CAMS INNOVATION FUND FOR MEDICAL SCIENCE	1	0.1
CANADIAN FOOD INSPECTION AGENCY S MANDATED GENOMICS AND RESEARC	1	0.1
CANARY FOUNDATION HOWARD HUGHES MEDICAL INSTITUTE	1	0.1
CANCER COUNCIL SOUTH AUSTRALIA	1	0.1
CANCER PREVENTION AND RESEARCH INSTITUTE OF TEXAS	1	0.1
CANCER PREVENTION RESEARCH INSTITUTE OF TEXAS HIHRRA	1	0.1

Table S4: Continued

Funding agency	Record count	% of 998
CARDIOVASCULAIR ONDERZOEK NEDERLAND CVON	1	0.1
CARL OG ELLEN HERTZ LEGAT TIL LAEGE OG NATURVI DENSKABEN	1	0.1
CAS GRANTS	1	0.1
CAS PIONEER HUNDRED TALENTS PROGRAM	1	0.1
CCT	1	0.1
CEA IRTELIS FELLOWSHIP	1	0.1
CENTER FOR STROKE RESEARCH BERLIN	1	0.1
CENTER LABORATORY OF JINAN UNIVERSITY	1	0.1
CENTER MEDICAL LABORATORY OF SHANDONG UNIVERSITY	1	0.1
CENTRAL GOVERNMENT OF LIAONING PROVINCE	1	0.1
CENTRAL LABORATORY OF CANGZHOU CENTRAL HOSPITAL	1	0.1
CENTRAL LABORATORY OF ZIBO CENTRAL HOSPITAL	1	0.1
CENTRAL PUBLIC INTEREST SCIENTIFIC INSTITUTION BASAL RESEARCH F	1	0.1
CENTRAL SOUTH UNIVERSITY	1	0.1
CENTRAL SOUTH UNIVERSITY IN CHINA	1	0.1
CENTRE FOR INTEGRATIVE SEQUENCING AT AARHUS UNIVERSITY ISEQ	1	0.1
CHANG GUNG MEMORIAL HOSPITAL	1	0.1
CHANGSHA SCIENCE AND TECHNOLOGY PROJECT	1	0.1
CHANGZHOU SCIENCE AND TECHNOLOGY PROJECT	1	0.1
CHANGZHOU SCIENCE AND TECHNOLOGY SUPPORT PROJECT SOCIAL DEVELOP	1	0.1
CHEN XIAOPING FOUNDATION FOR THE DEVELOPMENT OF SCIENCE AND TEC	1	0.1
CHINA GUANGHUA FOUNDATION	1	0.1
CHINA HEBEI 100 SCHOLARS SUPPORTING PROJECT	1	0.1
CHINA HUMAN PROTEOME PROJECT	1	0.1
CHINA NATIONAL GRAND S T SPECIAL PROJECT	1	0.1
CHINA POSTDOCTORAL SPECIAL FUND	1	0.1
CHINA SCHOLAR COUNCIL	1	0.1
CHINA YANGZHOU KEY RESEARCH PROJECT SOCIAL DEVELOPMENT PLAN	1	0.1
CHINESE ACADEMY OF AGRICULTURAL SCIENCES	1	0.1
CHINESE ACADEMY OF MEDICAL SCIENCES INITIATIVE FOR INNOVATIVE M	1	0.1
CHINESE FOUNDATION FOR HEPATITIS PREVENTION AND CONTROL PROJECT	1	0.1
CHINESE MEDICAL ASSOCIATION	1	0.1
CHONGQING NATURAL SCIENCE FOUNDATION OF CHINA	1	0.1
CIHR BRIDGE FUND	1	0.1
CLINICAL AND REHABILITATION RESEARCH FOUNDATION OF XIANGYA HOSP	1	0.1
CLINICAL CAPABILITY CONSTRUCTION PROJECT FOR LIAONING PROVINCIA	1	0.1
CLINICAL MEDICAL SCIENCE AND TECHNOLOGY DEVELOPMENT FUND OF JIA	1	0.1
CLINICAL RESEARCH FOUNDATION OF TMMU CHINA	1	0.1
CLINICAL SPECIAL FUND OF JIANGSU PROVINCE	1	0.1

Table S4: Continued

Funding agency	Record count	% of 998
CLL MOONSHOT FLAGSHIP PROJECT	1	0.1
CLOUD SEQ BIOTECH LTD CO SHANGHAI CHINA	1	0.1
CNPQ MCTI CNPQ UNIVERSAL	1	0.1
CNPQ PDI	1	0.1
CNPQ PRODUTIVIDADE	1	0.1
COLLEGE OF ANIMAL SCIENCE AND TECHNOLOGY GANSU AGRICULTURAL UNI	1	0.1
COMMITTEE OF SCIENCE AND TECHNOLOGY	1	0.1
CONSTRUCTION ENGINEERING SPECIAL FUND OF TAISHAN SCHOLARS	1	0.1
CONSTRUCTION PROJECT OF MODERN AGRICULTURAL SCIENCE AND TECHNOL	1	0.1
CREATIVE INNOVATION EXPERIMENT PROJECT OF COLLEGE STUDENTS	1	0.1
CREATIVE RESEARCH DEVELOPMENT GRANT FROM THE FIRST AFFILIATED H	1	0.1
CROP GERMPLASM CONSERVATION PROJECT FOUNDATION OF CHINA	1	0.1
CSC	1	0.1
CSCO LIZHU CHINESE MEDICINE CANCER RESEARCH FUND	1	0.1
CSIC OPEN ACCESS PUBLICATION SUPPORT INITIATIVE THROUGH ITS UNI	1	0.1
CULTIVATE NATIONAL SCIENCE FUND FOR DISTINGUISHED YOUNG SCHOLAR	1	0.1
CULTIVATE PROJECT FOR THE INTERDISCIPLINE AND EMERGING DISCIPLI	1	0.1
DAAD PPP PROGRAM	1	0.1
DAGMAR MARSHALLS FOND	1	0.1
DANISH CANCER SOCIETY	1	0.1
DANISH CENTER FOR SCIENTIFIC COMPUTING DCSC DEIC	1	0.1
DANISH MULTIPLE SCLEROSIS SOCIETY	1	0.1
DARTMOUTH STARTUP FUND	1	0.1
DEAN S FELLOWSHIP	1	0.1
DEBRA	1	0.1
DEEP DEUTSCHES EPIGENOM PROGRAMM	1	0.1
DEEP GERMAN EPIGENOME PROGRAMME	1	0.1
DEFENSE ADVANCED RESEARCH PROJECTS AGENCY DARPA	1	0.1
DEPARTMENT INTEGRATION FOUNDATION	1	0.1
DEPARTMENT OF APPLIED SCIENCE OF INDIAN INSTITUTE OF INFORMATIO	1	0.1
DEPARTMENT OF BIOTECHNOLOGY DBT INDIA	1	0.1
DEPARTMENT OF CHEMISTRY VACATION BURSARY SCHEME	1	0.1
DEPARTMENT OF EDUCATION GUANGDONG GOVERNMENT UNDER THE TOP TIER	1	0.1
DEPARTMENT OF EDUCATION IN HENAN PROVINCE	1	0.1
DEPARTMENT OF EDUCATION OF THE BASQUE GOVERNMENT	1	0.1
DEPARTMENT OF HEALTH OF JIANGXI PROVINCE PROJECT	1	0.1
DEPARTMENT OF HEALTH SCIENCES RESEARCH MAYO CLINIC ROCHESTER MN	1	0.1
DEPARTMENT OF INDUSTRY OF THE BASQUE COUNTRY	1	0.1
DEPARTMENT OF NEUROLOGICAL SURGERY UNIVERSITY OF WISCONSIN	1	0.1

Table S4: Continued

Funding agency	Record count	% of 998
DEPARTMENT OF SCIENCE AND TECHNOLOGY IN HENAN PROVINCE	1	0.1
DEPARTMENT OF SCIENCE AND TECHNOLOGY OF HENAN PROVINCE	1	0.1
DEPARTMENT OF SCIENCE AND TECHNOLOGY OF JILIN PROVINCE CHINA	1	0.1
DEPARTMENT OF SCIENCE AND TECHNOLOGY OF SHANDONG PROVINCE	1	0.1
DEPARTMENT OF SCIENCE TECHNOLOGY INDIA	1	0.1
DEPARTMENT OF SCIENCE TECHNOLOGY OF SHANDONG PROVINCE	1	0.1
DEPARTMENT OF SURGERY AT PENN STATE HEALTH MILTON S HERSHEY MED	1	0.1
DEUTSCHE FORSCHUNGSGEMEINSCHAFT EXCELLENCE INITIATIVE	1	0.1
DEUTSCHES EPIGENOM PROGRAMM DEEP	1	0.1
DEVELOPMENT AND REFORM COMMISSION OF HUNAN PROVINCE	1	0.1
DFG CLUSTER OF EXCELLENCE FOR MACROMOLECULAR COMPLEXES GOETHE U	1	0.1
DGK GERMAN SOCIETY OF CARDIOLOGY	1	0.1
DIRECTOR FUND OF JIANGSU KEY LABORATORY OF NEW DRUG RESEARCH AN	1	0.1
DIREKTOR EMIL C HERTZ OG HUSTRU INGER HERTZ FOND	1	0.1
DIVISION OF AIDS NATIONAL INSTITUTE OF ALLERGY AND INFECTIOUS D	1	0.1
DOCTORAL DEGREE ACTIVATION FEE FOR SCIENTIFIC RESEARCH PROJECTS	1	0.1
DOCTORAL INNOVATION FUND PROJECTS FROM SHANGHAI JIAO TONG UNIVE	1	0.1
DOCTORAL SCIENTIFIC RESEARCH FOUNDATION OF LIAONING PROVINCE	1	0.1
DOCTORAL SCIENTIFIC RESEARCH FOUNDATION OF LIAONING PROVINCE IN	1	0.1
DONALD E AND DELIA B BAXTER FOUNDATION	1	0.1
DONALD E AND DELIA B BAXTER FOUNDATION FACULTY SCHOLARS AWARD	1	0.1
DR MIRIAM AND SHELDON G ADELSON MEDICAL RESEARCH FOUNDATION	1	0.1
DUKE NUS SIGNATURE RESEARCH PROGRAM IN EMERGING INFECTIOUS DISE	1	0.1
DUNCAN CANCER CENTER	1	0.1
DUNHILL MEDICAL TRUST	1	0.1
DUTCH SCIENCE FOUNDATION LSBR FELLOWSHIP 1373	1	0.1
DUTCH SCIENCE FOUNDATION VIDI GRANT	1	0.1
E POLYTECHNIQUE	1	0.1
EARMARKED FUND FOR THE CHINA AGRICULTURE RESEARCH SYSTEM	1	0.1
EC CIRCTRAIN ITN NETWORK	1	0.1
EDUCATION AND SCIENTIFIC RESEARCH FUND OF YOUNG TEACHERS OF FUJ	1	0.1
EDUCATION AND SCIENTIFIC RESEARCH PROGRAM FUJIAN MINISTRY OF ED	1	0.1
EDUCATION DEPARTMENT OF HENAN PROVINCE	1	0.1
EDUCATION DEPARTMENT OF JILIN PROVINCE CHINA	1	0.1
EINSTEIN FOUNDATION	1	0.1
ELI EDYTHE BROAD CENTER OF REGENERATIVE MEDICINE AND STEM CELL	1	0.1
ELI LILLY	1	0.1
EMBO YIP IMOS	1	0.1
EMERSON RESEARCH FOUNDATION	1	0.1

Table S4: Continued

Funding agency	Record count	% of 998
ENGINEER RESEARCH AND DEVELOPMENT CENTER U S ARMY CORPS OF ENGI	1	0.1
ENGINEERING RESEARCH CENTER OF ECOLOGY AND AGRICULTURAL USE OF	1	0.1
ENRICHMENT PROGRAM FOR MINORITY SERVING INSTITUTION FACULTY THR	1	0.1
ENTREPRENEURSHIP AND INNOVATION LEADING TALENT PROGRAM OF QINGD	1	0.1
EPIGEN	1	0.1
ERANET RUS	1	0.1
ERC ADVANCED GRANT ANGIOLNC	1	0.1
ERC STG	1	0.1
ESCIENCE PROGRAM OF THE RESEARCH COUNCIL OF NORWAY	1	0.1
ESTATE OF C G JOHNSON	1	0.1
EURATOM FISSION EUROPEAN COMMISSION 7TH FRAMEWORK PROGRAMME DAR	1	0.1
EUROPEAN MOLECULAR BIOLOGY ORGANIZATION EMBO	1	0.1
EUROPEAN RESEARCH COUNCIL CONSOLIDATOR GRANT ERC	1	0.1
EUROPEAN SOCIAL FUND ESF	1	0.1
EUROPEAN UNION EUROPEAN SOCIAL FUND AS PART OF THE HUMAN RESOUR	1	0.1
EUROPEAN UNION SEVENTH FRAMEWORK PROJECT DIXA	1	0.1
EXCELLENT DOCTORAL DISSERTATION CULTIVATING FUND OF HUNAN AGRIC	1	0.1
EXCELLENT TALENT FUND PROJECT OF HIGHER EDUCATION OF LIAONING P	1	0.1
EXCELLENT YOUNG TEACHER PROJECT OF NANJING MEDICAL UNIVERSITY C	1	0.1
FABRIKANT EJNAR WILLUMSENS LEGAT	1	0.1
FACULTY OF HEALTH AND MEDICAL SCIENCES UNIVERSITY OF COPENHAGEN	1	0.1
FAPERGS CAPES DOCFIX	1	0.1
FEDERAL PROGRAM FOR SUPPORT OF SCIENTIFIC SCHOOLS OF THE RUSSIA	1	0.1
FINANCIAL SCHEME FOR APPLICATION TECHNOLOGY RESEARCH AND DEVELO	1	0.1
FIRST AFFILIATED HOSPITAL OF HARBIN MEDICAL UNIVERSITY SCIENCE	1	0.1
FLORIDA SEA GRANT FSG	1	0.1
FONDATION FRANCOPHONE POUR LA RECHERCHE SUR LE DIABETE FEDERATI	1	0.1
FONDATION LEDUCQ MIRVAD	1	0.1
FONDATION LEDUCQ MIRVAD MICRORNA BASED THERAPEUTIC STRATEGIES I	1	0.1
FONDAZIONE CITTA DELLA SPERANZA	1	0.1
FONDEN AF 1870	1	0.1
FOUNDATION FOR DISTINGUISHED YOUNG SCIENTISTS OF JIANGXI PROVIN	1	0.1
FOUNDATION FOR INNOVATION RESEARCH TEAM OF JIMEI UNIVERSITY	1	0.1
FOUNDATION OF DISCIPLINE LEADER IN HEALTH SYSTEMS OF PUDONG NEW	1	0.1
FOUNDATION OF EDUCATION BUREAU OF LIAONING PROVINCE	1	0.1
FOUNDATION OF HE NAN EDUCATIONAL COMMITTEE	1	0.1
FOUNDATION OF HEALTH DEPARTMENT OF HEILONGJIANG PROVINCE	1	0.1
FOUNDATION OF JIANGSU CANCER HOSPITAL	1	0.1
FOUNDATION OF JILIN PROVINCE EDUCATION DEPARTMENT	1	0.1

Table S4: Continued

Funding agency	Record count	% of 998
FOUNDATION OF KEY LABORATORY OF MYOCARDIAL ISCHEMIA MINISTRY OF	1	0.1
FOUNDATION OF MEDICAL SCIENCE AND TECHNOLOGY OF ZHEJIANG PROVIN	1	0.1
FOUNDATION OF NANJING CITY COMMITTEE OF SCIENCE AND TECHNOLOGY	1	0.1
FOUNDATION OF NANJING MEDICAL UNIVERSITY	1	0.1
FOUNDATION OF SCIENCE AND TECHNOLOGY DEPARTMENT OF JILIN PROVIN	1	0.1
FOUNDATION OF SCIENCE AND TECHNOLOGY PLANNING PROJECT OF TAIZHO	1	0.1
FOUNDATION OF SHANGHAI HEALTH BUREAU SHANGHAI PR CHINA	1	0.1
FOUNDATION OF UROLOGIC RESEARCH BERLIN	1	0.1
FOUNDATION OF ZHEJIANG PROVINCIAL KEY LABORATORY OF PATHOPHYSIO	1	0.1
FOURTH AFFILIATED HOSPITAL OF HARBIN MEDICAL UNIVERSITY	1	0.1
FP7 HEALTH INNOVATION 1 COLLABORATIVE PROJECT EPI MIRNA	1	0.1
FRAMEWORK PROGRAMME FP7	1	0.1
FREE STATE OF SAXONY	1	0.1
FREE STATE OF SAXONY WITHIN THE EXCELLENCE INITIATIVE	1	0.1
FRENCH MINISTERE DE L EDUCATION NATIONALE DE LA RECHERCHE ET DE	1	0.1
FUDAN CHILDREN S HOSPITAL AND FUDAN UNIVERSITY	1	0.1
FUJIAN PROVINCIAL MEDICAL INNOVATION PROJECT	1	0.1
FUJIAN PROVINCIAL NATURAL SCIENCE FOUNDATION	1	0.1
FUND FOR FOSTERING YOUNG SCHOLARS OF PEKING UNIVERSITY HEALTH S	1	0.1
FUND FOR SCIENTIFIC RESEARCH OF THE FIRST HOSPITAL OF CHINA MED	1	0.1
FUND OF ANHUI MEDICAL UNIVERSITY DOCTORAL START RESEARCH	1	0.1
FUND OF SOCIAL DEVELOPMENT IN JIANGSU PROVINCE	1	0.1
FUNDAMENTAL RESEARCH FUNDS FOR THE CENTRAL UNIVERSITIES AND SIN	1	0.1
FUNDAMENTAL RESEARCH FUNDS OF SHANDONG UNIVERSITY	1	0.1
FUNDAMENTAL RESEARCH PROGRAM FUNDING OF THE NINTH PEOPLE S HOSP	1	0.1
GATTEGNO FUND	1	0.1
GENERAL JOINT PROJECT OF YUNNAN PROVINCIAL SCIENCE AND TECHNOLO	1	0.1
GENERAL PROJECT OF THE TWELFTH FIVE YEAR PLAN OF PLA	1	0.1
GERMAN MINISTRY FOR EDUCATION AND RESEARCH SATNET PROGRAM	1	0.1
GRADUATE SCHOOL OF HEALTH AARHUS UNIVERSITY	1	0.1
GRADUATE STUDENT INNOVATION FOUNDATION OF JIANGSU PROVINCE	1	0.1
GRADUATE STUDENT S RESEARCH AND INNOVATION FUND OF SICHUAN UNIV	1	0.1
GRADUATES INNOVATION FUND HUAZHONG UNIVERSITY OF SCIENCE AND TE	1	0.1
GRANTS FROM INNOVATION TEAM PROJECT FUND OF JIANGSU PROVINCE	1	0.1
GRANTS IN AID FOR SCIENTIFIC RESEARCH KAKENHI	1	0.1
GUANG DONG NATURAL SCIENTIFIC FOUNDATION	1	0.1
GUANGDONG HIGH LEVEL PERSONNEL OF SPECIAL SUPPORT PROGRAM FOR O	1	0.1
GUANGDONG MEDICAL RESEARCH FOUNDATION	1	0.1
GUANGDONG PROVINCE COMPUTATIONAL SCIENCE INNOVATIVE RESEARCH TE	1	0.1

Table S4: Continued

Funding agency	Record count	% of 998
GUANGDONG PROVINCE SCIENCE AND TECHNOLOGY PROJECT	1	0.1
GUANGDONG PROVINCIAL DEPARTMENT OF SCIENCE AND TECHNOLOGY GUANG	1	0.1
GUANGDONG PROVINCIAL NATURAL SCIENCE FUND	1	0.1
GUANGXI MEDICAL UNIVERSITY TRAINING PROGRAM FOR DISTINGUISHED Y	1	0.1
GUANGZHOU PEOPLE S LIVELIHOOD SCIENCE AND TECHNOLOGY PROJECT	1	0.1
GUANGZHOU SCIENCE AND TECHNOLOGY PROGRAM	1	0.1
GUANGZHOU UNIVERSITY OF CHINESE MEDICINE HIGH LEVEL UNIVERSITY	1	0.1
GULF OF MEXICO RESEARCH INITIATIVE CENTER FOR ADVANCED RESEARCH	1	0.1
HAIYAN FUND OF HARBIN MEDICAL UNIVERSITY CANCER HOSPITAL	1	0.1
HAIYAN RESEARCH FUND OF THE THIRD AFFILIATED HOSPITAL OF HARBIN	1	0.1
HANDAN SCIENCE AND TECHNOLOGY RESEARCH AND DEVELOPMENT PROJECT	1	0.1
HARBIN SPECIAL FUNDS OF INNOVATIVE TALENTS ON SCIENCE AND TECHN	1	0.1
HARBOEFONDEN	1	0.1
HARVESTPLUS PROGRAMME	1	0.1
HBCU UP RESEARCH INITIATION AWARD FROM THE NATIONAL SCIENCE FOU	1	0.1
HEALTH AND FAMILY PLANNING COMMISSION OF CHONGQING CHINA	1	0.1
HEALTH AND FAMILY PLANNING SCIENCE AND TECHNOLOGY KEY PROJECT F	1	0.1
HEALTH MANAGEMENT DEPARTMENT OF JILIN PROVINCE CHINA	1	0.1
HEALTH RESEARCH INSTITUTION PROJECT OF YUNNAN PROVINCE	1	0.1
HEBEI MEDICAL UNIVERSITY RESEARCH DEVELOPMENT FUND	1	0.1
HEBEI PROVINCE HEALTH AND HEALTH COMMISSION MEDICAL SCIENCE RES	1	0.1
HEILONGJIANG POSTDOCTORAL FOUNDATION	1	0.1
HEILONGJIANG POSTDOCTORAL FUND	1	0.1
HEILONGJIANG PROVINCIAL ACADEMY OF MEDICAL SCIENCES	1	0.1
HELMHOLTZ ASSOCIATION FOR THE MDC SYSTEMS BIOLOGY NETWORK MSBN	1	0.1
HENAN PROVINCIAL DEPARTMENT OF EDUCATION	1	0.1
HENAN PROVINCIAL NATURAL SCIENCE FOUNDATION	1	0.1
HENAN SCHOLARSHIP COUNCIL	1	0.1
HIGH LEVEL INNOVATION TALENT GRANT TIANJIN MEDICAL UNIVERSITY	1	0.1
HIGH LEVEL LEADING TALENT INTRODUCTION PROGRAM OF GDAS	1	0.1
HIGH LEVEL TALENT FOUNDATION OF HEBEI PROVINCE	1	0.1
HIGH LEVEL TALENT PROGRAM OF CENTRAL SOUTH UNIVERSITY	1	0.1
HIGH LEVEL TALENT PROJECT OF COLLEGES AND UNIVERSITIES IN GUANG	1	0.1
HIGHER EDUCATION COMMISSION OF PAKISTAN	1	0.1
HIRSCHL WEILL CAULIER TRUST	1	0.1
HONG KONG SCHOLARS PROGRAM	1	0.1
HOPKINS DIGESTIVE DISEASES BASIC AND TRANSLATIONAL RESEARCH COR	1	0.1
HOUSTON ENDOWMENT UNDER THE SEVERE STORM PREDICTION EDUCATION A	1	0.1

Table S4: Continued

Funding agency	Record count	% of 998
HUAZHONG AGRICULTURAL UNIVERSITY SCIENTIFIC TECHNOLOGICAL SELF	1	0.1
HUBEI COLLABORATIVE INNOVATION CENTER FOR GRAIN INDUSTRY	1	0.1
HUBEI PROVINCE KEY LABORATORY OF OCCUPATIONAL HAZARD IDENTIFICA	1	0.1
HUBEI PROVINCIAL NATURAL SCIENCE FOUNDATION FOR INNOVATIVE GROU	1	0.1
HUMAN RESOURCE SUMMIT GRANT OF JIANGSU PROVINCE	1	0.1
HUMANITIES AND SOCIAL SCIENCE RESEARCH PROJECT OF COLLEGES AND	1	0.1
HUNAN PROVINCIAL INNOVATION FOUNDATION FOR POSTGRADUATE	1	0.1
ICAHN INSTITUTE FOR GENOMICS AND MULTISCALE BIOLOGY	1	0.1
ICHIHARA INTERNATIONAL SCHOLARSHIP	1	0.1
INAMORI FOUNDATION	1	0.1
INDEPENDENT RESEARCH FUND DENMARK	1	0.1
INDIAN COUNCIL OF MEDICAL RESEARCH	1	0.1
INDUSTRY ACADEMY RESEARCH COOPERATION PROJECT OF SHANGHAI SCIEN	1	0.1
INDUSTRY UNIVERSITY RESEARCH COOPERATION PROSPECTIVE JOINT RESE	1	0.1
INITIAL FUNDING FOR NEW PI	1	0.1
INITIALIZING FUND OF XUZHOU MEDICAL UNIVERSITY	1	0.1
INITIATIVE AND NETWORKING FUND OF THE HELMHOLTZ ASSOCIATION	1	0.1
INNOVATION AND ENTREPRENEURSHIP LEADING TALENT OF GUANGZHOU DEV	1	0.1
INNOVATION FUND DENMARK	1	0.1
INNOVATION FUND DENMARK DANISH COUNCIL FOR INDEPENDENT RESEARCH	1	0.1
INNOVATION FUND OF JILIN UNIVERSITY	1	0.1
INNOVATION PROJECT OF SHANDONG ACADEMY OF MEDICAL SCIENCE	1	0.1
INNOVATION TEAM DEVELOPMENT PROGRAM OF THE MINISTRY OF EDUCATIO	1	0.1
INNOVATION TEAM FOUNDATION OF FUZHOU GENERAL HOSPITAL	1	0.1
INNOVATIVE ENGINEERING PROJECTS FOR SCIENCE AND TECHNOLOGY COOR	1	0.1
INNOVATIVE FUNDS FOR SMALL AND MEDIUM SIZED ENTERPRISES OF GUAN	1	0.1
INNOVATIVE TEAM PROGRAM OF ZHENGZHOU SCIENCE AND TECHNOLOGY BUR	1	0.1
INSTITUT NATIONAL DE LA SANTE ET DE LA RECHERCHE MEDICALE INSER	1	0.1
INSTITUTE OF WATER RESOURCES	1	0.1
INTERNATIONAL COOPERATION FUND PROJECT OF BEIJING ACADEMY OF AG	1	0.1
INTERNATIONAL COOPERATION PROJECTS OF MOE	1	0.1
INTERNATIONAL FOUNDATION FOR SCIENCE	1	0.1
INTERNATIONAL FOUNDATION OF TRANSLATIONAL MEDICINE FOR ABROAD S	1	0.1
INTERNATIONAL FOUNDATION OF TRANSLATIONAL MEDICINE FOR ABROAD S	1	0.1
INTERNATIONAL HUMAN FRONTIERS SCIENCE PROGRAM ORGANIZATION CDA	1	0.1
INTERNATIONAL POSTDOCTORAL EXCHANGE FELLOWSHIP	1	0.1
INTERNATIONAL SCIENCE AND TECHNOLOGY COOPERATION PROJECT KEY RE	1	0.1
INTERNATIONAL SCIENCE AND TECHNOLOGY COOPERATION PROJECT OF GUA	1	0.1
INTERNATIONAL SCIENCE AND TECHNOLOGY CORPORATION PROGRAM OF CHI	1	0.1

Table S4: Continued

Funding agency	Record count	% of 998
INTERNATIONAL SCIENTIFIC AND TECHNOLOGICAL COOPERATION PROJECTS	1	0.1
INTEROMICS FLAGSHIP PROJECT CONSIGLIO NAZIONALE DELLE RICERCHE	1	0.1
INTRAMURAL RESEARCH PROGRAM OF THE NATIONAL LIBRARY OF MEDICINE	1	0.1
IRISH CANCER SOCIETY	1	0.1
ISRAELI SCIENCE FOUNDATION PERSONAL GRANT	1	0.1
JAMES HUDSON BROWN ALEXANDER BROWN COXE FELLOWSHIP	1	0.1
JAPAN HEALTH FOUNDATION	1	0.1
JBC FELLOWSHIP	1	0.1
JCIC MCP PROJECT	1	0.1
JIANGSU AGRICULTURAL SCIENCE AND TECHNOLOGY INDEPENDENT INNOVAT	1	0.1
JIANGSU CLINICAL RESEARCH CENTER FOR CARDIOVASCULAR SURGERY	1	0.1
JIANGSU COLLABORATIVE INNOVATION CENTER FOR MODERN CROP PRODUCT	1	0.1
JIANGSU HEALTH DEPARTMENT	1	0.1
JIANGSU KEY LABORATORY OF NEW DRUG RESEARCH AND CLINICAL PHARMA	1	0.1
JIANGSU MATERNAL AND CHILD HEALTH RESEARCH PROJECT	1	0.1
JIANGSU PROVINCE FOR OUTSTANDING SCI TECH INNOVATION TEAM IN CO	1	0.1
JIANGSU PROVINCE S KEY DISCIPLINE LABORATORY OF MEDICINE	1	0.1
JIANGSU PROVINCE SCIENCE AND TECHNOLOGY SUPPORT PROGRAM	1	0.1
JIANGSU PROVINCE SIX TALENT PEAK PERSONAL TRAINING PROJECT	1	0.1
JIANGSU PROVINCIAL COMMISSION OF HEALTH AND FAMILY PLANNING	1	0.1
JIANGSU PROVINCIAL DEPARTMENT OF EDUCATION	1	0.1
JIANGSU PROVINCIAL HIGHER EDUCATION NATURAL SCIENCE RESEARCH MA	1	0.1
JIANGSU PROVINCIAL MEDICAL INNOVATION TEAM OF THE PROJECT OF IN	1	0.1
JIANGSU PROVINCIAL MEDICAL YOUTH TALENT PROJECT	1	0.1
JIANGSU PROVINCIAL NATURAL SCIENCE FOUNDATION YOUTH FOUNDATION	1	0.1
JIANGSU PROVINCIAL PROGRAM FOR MEDICAL INNOVATION TEAMS AND LEA	1	0.1
JIANGSU PROVINCIAL SCIENCE AND TECHNOLOGY DEPARTMENT CLINICAL S	1	0.1
JIANGSU PROVINCIAL WOMEN AND CHILDREN HEALTH RESEARCH PROJECT	1	0.1
JIANGSU SIX TYPE TOP TALENTS PROGRAM	1	0.1
JIANGSU SPECIALLY APPOINTED PROFESSOR	1	0.1
JIANGSU SPECIALLY APPOINTED PROFESSOR GRANTS	1	0.1
JIANGSU UNIVERSITY SENIOR PROFESSIONAL SCIENCE FOUNDATION	1	0.1
JIANGSU YOUTH MEDICAL TALENTS	1	0.1
JIANGXI PROVINCE EDUCATION FUND PROJECT	1	0.1
JIANGXI PROVINCE GENERAL PROJECT	1	0.1
JIANGXI PROVINCIAL NATURAL SCIENCE FOUNDATION OF CHINA	1	0.1
JILIN PROVINCIAL HEALTH DEPARTMENT	1	0.1
JILIN PROVINCIAL KEY LABORATORY OF BIOLOGICAL THERAPY	1	0.1
JILIN SCIENCE AND TECHNIQUE INTERNATIONAL COLLABORATION GRANT	1	0.1

Table S4: Continued

Funding agency	Record count	% of 998
JILIN UNIVERSITY XINJIANG MEDICAL UNIVERSITY	1	0.1
JIMB SEED GRANT	1	0.1
JINAN SCIENCE AND TECHNOLOGY BUREAU	1	0.1
JOHNS HOPKINS BLOOMBERG SCHOOL OF PUBLIC HEALTH START UP FUND	1	0.1
JOINT RESEARCH FUND DEVOTED TO CLINICAL PHARMACY AND PRECISION	1	0.1
JUDITH AND JEAN PAPE ADAMS FOUNDATION	1	0.1
JUVENILE DIABETES RESEARCH FOUNDATION JDRF POSTDOCTORAL FELLOWS	1	0.1
K C WONG EDUCATION FOUNDATION HONG KONG	1	0.1
K C WONG MAGNA FUND AT NINGBO UNIVERSITY	1	0.1
K C WONG MAGNA FUND IN NINGBO UNIVERSITY NINGBO SOCIAL DEVELOPM	1	0.1
KAUST THE KING ABDULLAH UNIVERSITY OF SCIENCE AND TECHNOLOGY	1	0.1
KC WONG MAGNA FUND IN NINGBO UNIVERSITY	1	0.1
KEY DISCIPLINE AND SPECIALTY FOUNDATION OF SHANGHAI MUNICIPAL C	1	0.1
KEY DISCIPLINE OF PUDONG NEW AREA SHANGHAI PWZXK2017 18	1	0.1
KEY DRUG DEVELOPMENT PROGRAMME OF MOST	1	0.1
KEY LABORATORIES OF EDUCATION MINISTRY FOR MYOCARDIAL ISCHEMIA	1	0.1
KEY LABORATORY OF ENVIRONMENTAL POLLUTION MONITORING AND DISEAS	1	0.1
KEY LABORATORY OF SHENZHEN	1	0.1
KEY LABORATORY OF YUNNAN PROVINCE FOR OPHTHALMIC RESEARCH AND D	1	0.1
KEY MEDICAL DISCIPLINES AND SPECIALTIES PROGRAM OF GUANGZHOU	1	0.1
KEY NATURAL SCIENCE FOUNDATION OF GUANGDONG	1	0.1
KEY NEW DRUG CREATION AND DEVELOPMENT PROGRAM OF CHINA	1	0.1
KEY PROGRAM FOR INTERNATIONAL S T COOPERATION FROM SCIENCE TECH	1	0.1
KEY PROGRAM FOR THE EXCELLENT YOUTH SCHOLARS OF ANHUI PROVINCE	1	0.1
KEY PROJECT OF CUTTING EDGE CLINICAL TECHNOLOGY OF JIANGSU PROV	1	0.1
KEY PROJECT OF CUTTINGEDGE CLINICAL TECHNOLOGY OF JIANGSU PROVI	1	0.1
KEY PROJECT OF DEPARTMENT OF EDUCATION OF YUNNAN PROVINCE	1	0.1
KEY PROJECT OF JIANGSU PROVINCE	1	0.1
KEY PROJECT OF JIANGXI PROVINCIAL DEPARTMENT OF EDUCATION	1	0.1
KEY PROJECT OF LOGISTICS RESEARCH PLAN OF PLA	1	0.1
KEY PROJECT OF NATIONAL NATURAL SCIENCE FUND GUANGDONG PROVINCE	1	0.1
KEY PROJECT OF SCIENCE AND TECHNOLOGY OF SHANDONG PROVINCE	1	0.1
KEY PROJECT OF SOCIAL DEVELOPMENT IN JIANGSU PROVINCE	1	0.1
KEY PROJECT OF SOCIAL DEVELOPMENT OF XUZHOU CITY CHINA	1	0.1
KEY PROJECTS OF THE XIAMEN SOUTHERN OCEAN RESEARCH CENTER	1	0.1
KEY PROJECTS OF TIANJIN MUNICIPAL COMMISSION OF HEALTH AND FAMI	1	0.1
KEY RESEARCH AND DEVELOPMENT PLAN MODERN AGRICULTURE IN JIANGSU	1	0.1
KEY RESEARCH AND DEVELOPMENT PROGRAM OF JIANGSU PROVINCE	1	0.1
KEY RESEARCH AND DEVELOPMENT PROGRAM OF SHANDONG PROVINCE	1	0.1

Table S4: Continued

Funding agency	Record count	% of 998
KEY RESEARCH DEVELOPMENT PLAN OF SHAANXI PROVINCE OF CHINA GENE	1	0.1
KEY RESEARCH PROJECT OF YANTAI CITY	1	0.1
KEY RESEARCH TECHNOLOGY PROJECTS IN JIANGSU PROVINCE	1	0.1
KEY SCIENCE AND TECHNOLOGY PROGRAM OF SHAANXI PROVINCE CHINA	1	0.1
KEY TECHNOLOGY RESEARCH AND DEVELOPMENT PROGRAM OF THE SICHUAN	1	0.1
KING ABDULLAH UNIVERSITY OF SCIENCE TECHNOLOGY	1	0.1
KLAUS TSCHIRA STIFTUNG GGMBH	1	0.1
KNOWLEDGE INNOVATION PROGRAM OF INSTITUTE OF HYDROBIOLOGY CHINE	1	0.1
KNUT AND ALICE WALLENBERG FOUNDATION AS PART OF THE NATIONAL BI	1	0.1
KOEBMAND KRISTIAN KJAERS FOUNDATION	1	0.1
KOREA HEALTHCARE TECHNOLOGY R D PROJECT MINISTRY OF HEALTH WELF	1	0.1
KOREAN HEALTH TECHNOLOGY RAMP D PROJECT MINISTRY OF HEALTH AND	1	0.1
KURATA MEMORIAL HITACHI SCIENCE AND TECHNOLOGY FOUNDATION	1	0.1
KYUSHU UNIVERSITY INTERDISCIPLINARY PROGRAMS IN EDUCATION	1	0.1
LABEX GRAL	1	0.1
LADIES LEUKEMIA LEAGUE GRANT	1	0.1
LANDSTEINER FOUNDATION OF BLOOD TRANSFUSION RESEARCH	1	0.1
LANZHOU TALENT INNOVATION AND ENTREPRENEURSHIP PROJECT	1	0.1
LEADER TRAINING PROGRAM IN MEDICAL SUBJECTS OF HEALTH AND FAMIL	1	0.1
LEADING ACADEMIC DISCIPLINE PROJECT OF BEIJING EDUCATION BUREAU	1	0.1
LEDUCQ FOUNDATION NETWORK MIRVAD	1	0.1
LEON AND BERTHA GOLBERG FOUNDATION	1	0.1
LEVERHULME TRUST	1	0.1
LEVERHULME TRUST IN THE FORM OF AN EARLY CAREER FELLOWSHIP FOR	1	0.1
LIFE LEIPZIG RESEARCH CENTER FOR CIVILIZATION DISEASES LEIPZIG	1	0.1
LIFE PROJECT EUROPEAN UNION	1	0.1
LIVER AND AIDS FUND OF BEIJING YOUAN HOSPITAL	1	0.1
LOEWE CENTER FOR CELL AND GENE THERAPY STATE OF HESSEN	1	0.1
LOEWE PROGRAMME MEDICAL RNOMICS STATE OF HESSEN	1	0.1
LOUISIANA BIOTECHNOLOGY RESEARCH NETWORK LBRN	1	0.1
LOUISIANA BOR RCS	1	0.1
LUCILLE P MARKEY BIOMEDICAL RESEARCH FELLOWSHIP	1	0.1
LUCILLE P MARKEY CHARITABLE TRUST	1	0.1
LUPUS RESEARCH ALLIANCE	1	0.1
LUXEMBOURG NATIONAL RESEARCH FUND	1	0.1
MAJOR NEW VARIETIES OF AGRICULTURAL PROJECTS IN JIANGSU PROVINC	1	0.1
MAJOR PROJECT OF YUNNAN PROVINCIAL BUREAU OF EDUCATION	1	0.1
MAJOR SCIENCE AND TECHNOLOGY SPECIAL PROJECT OF CHINA TWELFTH F	1	0.1
MAJOR SCIENTIFIC AND TECHNOLOGICAL PROJECT OF CHANGZHOU MUNICIP	1	0.1

Table S4: Continued

Funding agency	Record count	% of 998
MATHEMATICAL SCIENCES RESEARCH INSTITUTE MSRI IN BERKELEY CALIF	1	0.1
MAX DELBRUCK CENTER FOR MOLECULAR MEDICINE GRADUATE PROGRAM	1	0.1
MAX DELBRUCK CENTER MDC	1	0.1
MAYO CLINIC CENTER FOR INDIVIDUALIZED MEDICINE	1	0.1
MCCORMICK GABILAN AND A BAXTER FAMILY FELLOWSHIP	1	0.1
MCCORMICK GABILAN FELLOWSHIP	1	0.1
MCCORMICK GABILAN FELLOWSHIP STANFORD UNIVERSITY SCHOOL OF MEDI	1	0.1
MDC NYU	1	0.1
MDC NYU PHD EXCHANGE PROGRAMME	1	0.1
MEDICAL AND HEALTH TECHNOLOGY PROJECTS OF GUANGZHOU	1	0.1
MEDICAL DEVELOPMENT FUND OF BEIJING	1	0.1
MEDICAL RESEARCH FOUNDATION OF GUANGDONG PROVINCE CHINA	1	0.1
MEDICAL RESEARCH PROJECT OF THE AFFILIATED HOSPITAL OF MEDICAL	1	0.1
MEDICAL SCIENCE AND TECHNOLOGY RESEARCH FOUNDATION OF GUANGDONG	1	0.1
MEDICAL SCIENTIFIC AND TECHNOLOGICAL RESEARCH PROJECT OF HENAN	1	0.1
MEDICAL SCIENTIFIC RESEARCH KEY FOUNDATION OF NANJING COMMAND	1	0.1
MEDICINE AND HEALTH SCIENCE TECHNOLOGY FOUNDATION OF SHANDONG P	1	0.1
MEDJADEN ACADEMY RESEARCH FOUNDATION FOR YOUNG SCIENTISTS	1	0.1
MEGA PROJECTS OF NATIONAL SCIENCE RESEARCH FOR THE 13TH FIVE YE	1	0.1
MERCK COMPANY	1	0.1
MERCK CREAT PROJECT	1	0.1
MEXICAN COUNCIL OF SCIENCES AND TECHNOLOGY CONACYT	1	0.1
MEXICAN FEDERAL FUNDS	1	0.1
MILSTEIN MEDICAL ASIAN AMERICAN PARTNERSHIP MMAAP FOUNDATION RE	1	0.1
MINERVA FOUNDATION	1	0.1
MINISTERO DELLA SALUTE RICERCA CORRENTE 5X1000	1	0.1
MINISTRY OF AGRICULTURE CHINA	1	0.1
MINISTRY OF HEALTH SINGAPORE	1	0.1
MINISTRY OF HIGHER EDUCATION GOVERNMENT OF MALAYSIA	1	0.1
MINISTRY OF HIGHER EDUCATION SCIENTIFIC RESEARCH MHESR	1	0.1
MINISTRY OF HUMAN RESOURCE DEVELOPMENT	1	0.1
MINISTRY OF OCEANS AND FISHERIES KOREA	1	0.1
MINISTRY OF SCIENCE AND INNOVATION SPAIN MICINN	1	0.1
MINISTRY OF SCIENCE AND TECHNOLOGY S HIGH TECH 863 GRANT	1	0.1
MISHIMA KAIUN MEMORIAL FOUNDATION	1	0.1
MJF FOUNDATION	1	0.1
MODERN AGRICULTURAL INDUSTRY TECHNOLOGY SYSTEM	1	0.1

Table S4: Continued

Funding agency	Record count	% of 998
NAJRAN UNIVERSITY SAUDI ARABIA	1	0.1
NANFANG HOSPITAL	1	0.1
NANHU SCHOLARS PROGRAM OF XYNU	1	0.1
NANJING MEDICAL SCIENCE AND TECHNIQUE DEVELOPMENT FOUNDATION	1	0.1
NANJING SCIENCE AND TECHNOLOGY FUND	1	0.1
NANJING SCIENCE AND TECHNOLOGY PROJECT	1	0.1
NANTONG MUNICIPAL SCIENCE AND TECHNOLOGY BUREAU MAJOR PROJECT	1	0.1
NATION KEY RESEARCH AND DEVELOPMENT PROGRAM OF CHINA	1	0.1
NATIONAL 863 PROGRAM OF CHINA	1	0.1
NATIONAL BREAST CANCER FOUNDATION NBCF	1	0.1
NATIONAL CENTER OF COMPETENCE IN RESEARCH NCCR RNA AND DISEASE	1	0.1
NATIONAL CHIAO TUNG UNIVERSITY	1	0.1
NATIONAL CLINICAL KEY SPECIALTY OF CARDIOVASCULAR SURGERY	1	0.1
NATIONAL COLLEGE STUDENTS INNOVATION AND ENTREPRENEURSHIP TRAIN	1	0.1
NATIONAL DEVELOPMENT AND REFORM COMMISSION	1	0.1
NATIONAL HEALTH DEVELOPMENT PLANNING COMMISSION MAJOR DISEASE P	1	0.1
NATIONAL HEALTH RESEARCH INSTITUTES JAPAN	1	0.1
NATIONAL HIGH TECH R D PROGRAM 863 PROGRAM	1	0.1
NATIONAL HIGH TECH R D PROGRAM OF CHINA 863 PROGRAM	1	0.1
NATIONAL INSTITUTE OF ADVANCED INDUSTRIAL SCIENCE AND TECHNOLOG	1	0.1
NATIONAL INSTITUTE OF GENERAL MEDICAL SCIENCES FROM THE NATIONA	1	0.1
NATIONAL INSTITUTE ON AGING NIA OF THE NATIONAL INSTITUTES OF H	1	0.1
NATIONAL KEY BASIC RESEARCH AND DEVELOPMENT PLAN 973	1	0.1
NATIONAL KEY BASIC RESEARCH PROGRAM 973 PROGRAM PEOPLE S REPUBL	1	0.1
NATIONAL KEY BASIC RESEARCH PROGRAM 973 PROJECT FROM THE MINIST	1	0.1
NATIONAL KEY RESEARCH AND DEVELOPMENT PLAN	1	0.1
NATIONAL KEY RESEARCH AND DEVELOPMENT PLAN OF CHINA	1	0.1
NATIONAL KEY RESEARCH AND DEVELOPMENT PLAN PRECISION MEDICINE R	1	0.1
NATIONAL KEY RESEARCH AND DEVELOPMENT PROGRAMS	1	0.1
NATIONAL KEY RESEARCH DEVELOPMENT PROGRAM OF CHINA	1	0.1
NATIONAL KEY RESEARCH PROGRAM OF CHINA	1	0.1
NATIONAL KEY SCI TECH SPECIAL PROJECT OF CHINA	1	0.1
NATIONAL MEDICAL RESEARCH COUNCIL SINGAPORE	1	0.1
NATIONAL NATURAL FUND YOUTH SCIENCE FUND PROJECT	1	0.1
NATIONAL NATURAL SCIENCE FOUNDATION OF CHINA YOUTH FUND PROJECT	1	0.1
NATIONAL NATURAL SCIENCE FOUNDATION OF CHINA YOUTH SCIENCE FOUN	1	0.1
NATIONAL NATURAL SCIENCE FOUNDATION OF JIANGSU	1	0.1
NATIONAL OCEANIC ATMOSPHERIC ADMIN NOAA USA	1	0.1
NATIONAL POSTDOCTORAL PROGRAM FOR INNOVATIVE TALENTS	1	0.1
NATIONAL PROGRAM ON KEY RESEARCH PROJECT OF CHINA	1	0.1

Table S4: Continued

Funding agency	Record count	% of 998
NATIONAL SCI TECH SUPPORT PLAN	1	0.1
NATIONAL SCIENCE AND TECHNOLOGY SUPPORT PROGRAM	1	0.1
NATIONAL SCIENCE FOUNDATION AT THE PITTSBURGH SUPERCOMPUTING CE	1	0.1
NATIONAL SCIENCE FOUNDATION OF EDUCATION DEPARTMENT OF ANHUI PR	1	0.1
NATIONAL SCIENCE FOUNDATION OF HEBEI PROVINCE	1	0.1
NATIONAL SCIENCE FOUNDATION PROJECTS OF GUANGDONG PROVINCE	1	0.1
NATIONAL SCIENCE FUND	1	0.1
NATIONAL SCIENCE TECHNOLOGY SUPPORT PLAN PROJECTS CHINA	1	0.1
NATIONAL SCIENTIFIC FOUNDATION	1	0.1
NATIONAL SCIENTIFIC RESEARCH FOUNDATION OF LIAONING PROVINCE IN	1	0.1
NATIONAL SPARK PLAN	1	0.1
NATIONAL SUPPORT PROGRAM OF CHINA	1	0.1
NATIONAL TRAINING PROGRAMS OF INNOVATION AND ENTREPRENEURSHIP F	1	0.1
NATIONAL TRANSGENIC PROJECT	1	0.1
NATIONAL TRANSGENIC PROJECT OF CHINA	1	0.1
NATIONAL UNDERGRADUATE TRAINING PROGRAM FOR INNOVATION AND ENTR	1	0.1
NATIONAL YOUNG SCHOLARS SCIENCE FOUNDATION OF CHINA	1	0.1
NATIONAL YOUNG TOPNOTCH TALENT PROGRAM	1	0.1
NATURAL SCIENCE BASIC RESEARCH PLAN OF SHAANXI PROVINCE CHINA	1	0.1
NATURAL SCIENCE FOUNDATION FOR COLLEGES AND UNIVERSITIES IN JIA	1	0.1
NATURAL SCIENCE FOUNDATION FOR YOUNG SCHOLARS OF WANNAN MEDICAL	1	0.1
NATURAL SCIENCE FOUNDATION OF HEBEI PROVINCE	1	0.1
NATURAL SCIENCE FOUNDATION OF HENAN PROVINCE	1	0.1
NATURAL SCIENCE FOUNDATION OF HUBEI PROVINCE KEY PROJECTS OF TE	1	0.1
NATURAL SCIENCE FOUNDATION OF HUNAN PROVINCIAL	1	0.1
NATURAL SCIENCE FOUNDATION OF JILIN SCIENCE AND TECHNIQUE	1	0.1
NATURAL SCIENCE FOUNDATION OF SHANXI PROVINCE	1	0.1
NATURAL SCIENCE FOUNDATION OF THE ANHUI PROVINCIAL HIGH EDUCATI	1	0.1
NATURAL SCIENCE FOUNDATION OF THE HIGHER EDUCATION INSTITUTIONS	1	0.1
NATURAL SCIENCE FOUNDATION OF XIAMEN	1	0.1
NATURAL SCIENCE FUNDING OF SHAANXI PROVINCE	1	0.1
NCI FROM THE NIH	1	0.1
NE SOYBEAN BOARD FUNDS	1	0.1
NETHERLANDS CARDIOVASCULAR RESEARCH INITIATIVE	1	0.1
NETHERLANDS GENOMICS INITIATIVE NGI FROM THE NETHERLANDS ORGANI	1	0.1
NEUROSCIENCE RESEARCH AUSTRALIA	1	0.1
NEW XIANGYA TALENT PROJECT OF THE THIRD XIANGYA HOSPITAL OF CEN	1	0.1
NEW XIANGYA TALENT PROJECT OF THE THIRD XIANGYA HOSPITAL OF CEN	1	0.1
NHGRI MODENCODE PROJECT UNDER DOE	1	0.1

Table S4: Continued

Funding agency	Record count	% of 998
NIH FROM THE DEPARTMENT OF DEFENSE	1	0.1
NIH FROM THE TOBACCO RELATED DISEASE RESEARCH PROGRAM	1	0.1
NIH NATIONAL CENTER FOR RESEARCH RESOURCES NCRR	1	0.1
NIH NATIONAL INSTITUTE OF ALLERGY INFECTIOUS DISEASES NIAID	1	0.1
NIH NATIONAL INSTITUTE OF MENTAL HEALTH NIMH	1	0.1
NIH NATIONAL INSTITUTE OF NEUROLOGICAL DISORDERS STROKE NINDS	1	0.1
NIH NATIONAL INSTITUTE ON ALCOHOL ABUSE ALCOHOLISM NIAAA	1	0.1
NIH NATIONAL LIBRARY OF MEDICINE NLM	1	0.1
NIH OFFICE OF RESEARCH INFRASTRUCTURE PROGRAMS	1	0.1
NINGBO NATURAL SCIENCE FOUNDATION	1	0.1
NINGBO SCIENTIFIC INNOVATION TEAM FOR ENVIRONMENTAL HAZARDOUS F	1	0.1
NINGBO UNIVERSITY	1	0.1
NORMAN BETHUNE PROGRAM OF JILIN UNIVERSITY	1	0.1
NORTHWEST AF UNIVERSITY	1	0.1
NORWEGIAN CENTER FOR PREHOSPITAL EMERGENCY CARE NAKOS OSLO UNIV	1	0.1
NSF OFFICE OF THE DIRECTOR OD	1	0.1
NUPTSF	1	0.1
NYU MDC EXCHANGE PROGRAM	1	0.1
OFFICE OF RESEARCH SERVICES UNIVERSITY OF COLORADO DENVER	1	0.1
OPEN FOUNDATION OF NANJING UNIVERSITY	1	0.1
OPEN FUND OF KEY LABORATORY OF POLLINATING INSECT BIOLOGY OF MI	1	0.1
OPEN PROJECT OF BEIJING KEY LABORATORY OF RESPIRATORY AND PULMO	1	0.1
OPEN PROJECT OF STATE KEY LABORATORY OF FRESHWATER ECOLOGY AND	1	0.1
OPEN PROJECT PROGRAM OF ENGINEERING RESEARCH CENTER OF ECOLOGY	1	0.1
OPEN RESEARCH FUND OF STATE KEY LABORATORY OF HYBRID RICE HUNAN	1	0.1
OPENING FOUNDATION OF SHANGHAI KEY LABORATORY OF CRIME SCENE EV	1	0.1
OSU FACULTY DEVELOPMENT FUND	1	0.1
OTHER INDIVIDUAL RESEARCH GRANT	1	0.1
OUTSTANDING POSTGRADUATE DISSERTATION GROWTH FOUNDATION OF NINGBO	1	0.1
OUTSTANDING SCIENTIFIC FUND OF SHENGJING HOSPITAL	1	0.1
OUTSTANDING YOUNG TEACHERS PROJECT IN COLLEGES AND UNIVERSITIES	1	0.1
OUTSTANDING YOUTH PROJECT OF NANJING CITY	1	0.1
OUTSTANDING YOUTH PROJECT OF NATURAL SCIENCE FOUNDATION IN HUBEI	1	0.1
OVERSEAS EXPERTISE INTRODUCTION PROJECT FOR DISCIPLINE INNOVATI	1	0.1
OVERSEAS SCHOLARS EDUCATION DEPARTMENT OF HEILONGJIANG PROVINCE	1	0.1
PARENT PROJECT ITALIA	1	0.1
PARKER INSTITUTE FOR CANCER IMMUNOTHERAPY	1	0.1
PAUL GLENN FOUNDATION	1	0.1
PEKING TSINGHUA CENTER FOR LIFE SCIENCES	1	0.1

Table S4: Continued

Funding agency	Record count	% of 998
PEKING UNIVERSITY SCHOOL AND HOSPITAL OF STOMATOLOGY BEIJING CH	1	0.1
PENNSYLVANIA DEPARTMENT OF HEALTH	1	0.1
PLANT VIROLOGY RESEARCH CENTER COLLEGE OF AGRICULTURE SHIRAZ UN	1	0.1
POC GRANT CANTEMIR COMPETITIVITY OPERATIONAL PROGRAM	1	0.1
POST GRADUATE RESEARCH GRANT	1	0.1
POSTDOCTORAL FELLOWSHIP FROM THE BREAST CANCER FOUNDATION OF ON	1	0.1
POSTDOCTORAL FELLOWSHIP OF PEKING TSINGHUA CENTER FOR LIFE SCIE	1	0.1
POSTDOCTORAL INNOVATION FUND	1	0.1
POSTDOCTORAL SCIENCE FOUNDATION GRANT OF CHINA	1	0.1
POSTDOCTORAL SCIENCE FOUNDATION OF ANHUI PROVINCE CHINA	1	0.1
POSTDOCTORAL SCIENCE FOUNDATION OF CENTRAL SOUTH UNIVERSITY	1	0.1
POSTDOCTORAL SCIENTIFIC RESEARCH DEVELOPMENTAL FUND OF HEILONGJ	1	0.1
POSTGRADUATE INNOVATION PROJECT OF JIANGSU PROVINCE	1	0.1
POSTGRADUATE RESEARCH AND PRACTICE INNOVATION PROGRAM OF JIANGS	1	0.1
POSTGRADUATE RESEARCH AND PRACTICE INNOVATION PROGRAM OF JIANGS	1	0.1
PRESIDENT FOUNDATION OF NANFANG HOSPITAL SOUTHERN MEDICAL UNIVE	1	0.1
PRESIDENT FUND OF BEIJING LUHE HOSPITAL CAPITAL MEDICAL UNIVERS	1	0.1
PRESTO OF JST	1	0.1
PRIORITY ACADEMIC PROGRAM DEVELOPMENT OF JIANGSU HIGHER EDUCATI	1	0.1
PRIORITY ACADEMIC PROGRAM DEVELOPMENT OF JIANGSU HIGHER EDUCATI	1	0.1
PRIORITY ACADEMIC PROGRAM DEVELOPMENT OF JIANGSU HIGHER EDUCATI	1	0.1
PRIORITY ACADEMIC PROGRAM DEVELOPMENT OF JIANGSU HIGHER EDUCATI	1	0.1
PRIORITY ACADEMIC PROGRAM OF DEVELOPMENT OF JIANGSU HIGHER EDUC	1	0.1
PRIORITY ACADEMIC PROGRAM OF JIANGSU HIGHER EDUCATION INSTITUTI	1	0.1
PRODUCTION AND RESEARCH JOINT CULTIVATION PROJECT	1	0.1
PRODUCTIVITY AND RESEARCH GRANT FROM THE CONSELHO NACIONAL DE D	1	0.1
PROGRAM FOR EXCELLENT TALENTS IN HENAN PROVINCE	1	0.1
PROGRAM FOR INNOVATIVE RESEARCH TEAM IN SHAANXI PROVINCE	1	0.1
PROGRAM FOR LIAONING INNOVATIVE RESEARCH TEAM IN UNIVERSITY	1	0.1
PROGRAM FOR SHANGHAI OUTSTANDING MEDICAL ACADEMIC LEADER	1	0.1
PROGRAM FOR STUDENT S SCIENTIFIC RESEARCH AND INNOVATION EXPERI	1	0.1
PROGRAM FOR THE TOP YOUNG INNOVATIVE TALENTS OF SHANXI AGRICULT	1	0.1
PROGRAM OF JIANGSU PROVINCIAL MEDICAL INNOVATION TEAMS	1	0.1
PROGRAM OF NATIONAL INDUSTRIAL TECHNOLOGY SYSTEM FOR BEEF CATTL	1	0.1
PROGRAM OF NATIONAL NATURAL SCIENCE FOUNDATION	1	0.1
PROGRAM OF SHANGHAI ACADEMIC RESEARCH LEADER	1	0.1
PROGRAM OF YOUTH LEARNING BACKBONE TEACHER IN HENAN PROVINCE	1	0.1
PROGRAM OF ZHEJIANG MEDICAL TECHNOLOGY	1	0.1

Table S4: Continued

Funding agency	Record count	% of 998
PROJECT FUND OF HEALTH BUREAU OF JIANGSU PROVINCE	1	0.1
PROJECT FUND OF SCI TECH BUREAU OF HUAI AN CITY	1	0.1
PROJECT FUND OF TECHNOLOGY DEPARTMENT OF JIANGXI PROVINCE	1	0.1
PROJECT FUNDING FOR THE TRAINING OF HIGH LEVEL HEALTH PROFESSIO	1	0.1
PROJECT OF ADMINISTRATION OF TRADITIONAL CHINESE MEDICINE OF GU	1	0.1
PROJECT OF APPLICATION DEMONSTRATION CENTER OF PRECISION MEDICI	1	0.1
PROJECT OF DEPARTMENT OF HEALTH IN YUNNAN PROVINCE	1	0.1
PROJECT OF EDUCATION DEPARTMENT OF JILIN PROVINCE	1	0.1
PROJECT OF LIAONING PROVINCE EDUCATION DEPARTMENT	1	0.1
PROJECT OF NATIONAL CLINICAL KEY DISCIPLINE OF THE CHINESE MINI	1	0.1
PROJECT OF NATIONAL ESSENTIAL DRUG RESEARCH AND DEVELOPMENT OF	1	0.1
PROJECT OF NATIONAL NATURAL SCIENCE FUND OF CHINA	1	0.1
PROJECT OF SCIENCE AND TECHNOLOGY NEW STAR IN ZHUJIANG GUANGZHO	1	0.1
PROJECT OF SCIENTIFIC INNOVATION TEAM OF NINGBO	1	0.1
PROJECT OF SIX PEAK OF TALENTS OF JIANGSU PROVINCE OF CHINA	1	0.1
PROJECT OF SIX TALENT PEAKS OF JIANGSU PROVINCE	1	0.1
PROJECT OF THE AFFILIATED HOSPITAL OF LOGISTICS UNIVERSITY OF C	1	0.1
PROJECT OF TRADITIONAL MEDICAL SCIENCE AND TECHNOLOGY FOR SHAND	1	0.1
PROJECT OF ZHEJIANG EDUCATION DEPARTMENT NATURAL SCIENCES	1	0.1
PROJECTS IN RESEARCH DEVELOPMENT	1	0.1
PROJECTS OF SCIENCE AND TECHNOLOGY PLAN OF ZHANGZHOU CITY	1	0.1
PROPESP UFPA FADESP	1	0.1
PROSTATE CANCER FOUNDATION	1	0.1
PROVINCE NATURAL SCIENCE FOUNDATION OF GUANGDONG PROVINCE	1	0.1
PROVINCIAL GOVERNOR S SPECIAL FUND OF GUIZHOU PROVINCE	1	0.1
PROVINCIAL NATURAL SCIENCE FOUNDATION OF ANHUI	1	0.1
PROVINCIAL NATURAL SCIENCE RESEARCH PROJECT OF COLLEGES AND UNI	1	0.1
PROVINCIAL SCIENCE FUND OF JILIN PROVINCE DEVELOPMENT AND REFOR	1	0.1
PUBLIC SCIENCE AND TECHNOLOGY RESEARCH FUNDS PROJECTS OF OCEAN	1	0.1
PUBLIC TECHNOLOGY APPLIED RESEARCH PROGRAM OF ZHEJIANG PROVINCE	1	0.1
PUBLIC WELFARE PROJECT OF ZHEJIANG SCIENCE AND TECHNOLOGY DEPAR	1	0.1
PUDONG NEW DISTRICT COMMISSION OF HEALTH AND FAMILY PLANNING LE	1	0.1
PUMC YOUTH FUND	1	0.1
QATAR NATIONAL RESEARCH FUND	1	0.1
QING LAN PROJECT OF JIANGSU PROVINCE	1	0.1
QINGDAO SCIENCE AND TECHNOLOGY PLAN FUND	1	0.1
QUEENSLAND DEPARTMENT OF AGRICULTURE AND FISHERIES	1	0.1
RECRUITMENT PROGRAM OF GLOBAL EXPERTS CHINA	1	0.1
RECRUITMENT PROGRAM OF GLOBAL YOUNG EXPERTS 1000 PLAN	1	0.1

Table S4: Continued

Funding agency	Record count	% of 998
RESEARCH AND CREATIVE ACTIVITIES AWARD RACAS CU DENVER	1	0.1
RESEARCH FOUNDATION OF BEIJING FRIENDSHIP HOSPITAL CAPITAL MEDI	1	0.1
RESEARCH FOUNDATION OF HEALTH AND FAMILY PLANNING COMMISSION HU	1	0.1
RESEARCH FOUNDATION OF RIGSHOSPITALET	1	0.1
RESEARCH FOUNDATION OF SCIENCE TECHNOLOGY BUREAU OF LIWAN DISTR	1	0.1
RESEARCH FUND FOR THE DOCTORAL PROGRAM OF HIGHER EDUCATION OF C	1	0.1
RESEARCH FUND OF THE STATE KEY LABORATORY OF GENETIC ENGINEERIN	1	0.1
RESEARCH FUNDS FOR THE INNOVATION TEAM OF IRM CAMS	1	0.1
RESEARCH ON CHRONIC NONCOMMUNICABLE DISEASES PREVENTION AND CON	1	0.1
RESEARCH PROGRAM OF FOUNDATION AND ADVANCED TECHNOLOGY OF HENAN	1	0.1
RESEARCH PROJECT OF HEILONGJIANG PROVINCIAL HEALTH AND FAMILY P	1	0.1
RESEARCH SPECIAL FUND FOR PUBLIC WELFARE INDUSTRY OF HEALTH	1	0.1
RESEARCH SPECIAL FUND FOR PUBLIC WELFARE INDUSTRY OF HEALTH OF	1	0.1
RESEARCH TO PREVENT BLINDNESS RPB	1	0.1
RESEARCH TOPIC FOR DALI BAI AUTONOMOUS PREFECTURE COMMITTEE OF	1	0.1
RISING TIDE FOUNDATION	1	0.1
RISK PROJECT	1	0.1
RITA ALLEN FOUNDATION SCHOLAR	1	0.1
ROSE HILLS FOUNDATION RESEARCH AWARD	1	0.1
ROYAL ADELAIDE HOSPITAL RESEARCH FUND	1	0.1
SA SIBS SCHOLARSHIP PROGRAM	1	0.1
SAIL PLAN FOUNDATION FOR HIGH LEVEL PERSONNEL TRAINING PROJECT	1	0.1
SAN MING PROJECT OF MEDICINE IN SHENZHEN	1	0.1
SANJIN TALENT PROGRAM	1	0.1
SANMING PROJECT OF MEDICINE IN SHENZHEN	1	0.1
SANOFI AVENTIS	1	0.1
SAPERE AUDE PROGRAM OF THE INDEPENDENT RESEARCH FUND DENMARK ME	1	0.1
SC COASTAL EROSION STUDY	1	0.1
SCHIZOPHRENIA RESEARCH INSTITUTE	1	0.1
SCHOOL HOSPITAL JOINT FOSTERING FUND OF ZHENGZHOU UNIVERSITY HE	1	0.1
SCI TECH INNOVATION FOUNDATION OF HENAN AGRICULTURAL UNIVERSITY	1	0.1
SCI TECH RESEARCH PROJECT OF NINGBO	1	0.1
SCIENCE AND EDUCATION PROJECT OF JIANGSU PROVINCIAL COMMISSION	1	0.1
SCIENCE AND TECHNOLOGY COMMISSION FOUNDATION OF TONGZHOU DISTRI	1	0.1
SCIENCE AND TECHNOLOGY COMMITTEE OF CHONGQING CHINA	1	0.1
SCIENCE AND TECHNOLOGY DEVELOPMENT FOUNDATION OF DONGGUAN CITY	1	0.1
SCIENCE AND TECHNOLOGY DEVELOPMENT FOUNDATION OF SHANDONG PROVI	1	0.1
SCIENCE AND TECHNOLOGY DEVELOPMENT FUND OF BENGBU MEDICAL COLLE	1	0.1

Table S4: Continued

Funding agency	Record count	% of 998
SCIENCE AND TECHNOLOGY DEVELOPMENT PROJECT OF NANJING MEDICAL U	1	0.1
SCIENCE AND TECHNOLOGY DEVELOPMENT PROJECT OF NANTONG CITY	1	0.1
SCIENCE AND TECHNOLOGY FOUNDATION OF SICHUAN PROVINCE	1	0.1
SCIENCE AND TECHNOLOGY FUND OF GUANGDONG PROVINCE	1	0.1
SCIENCE AND TECHNOLOGY FUND OF GUANGZHOU	1	0.1
SCIENCE AND TECHNOLOGY INNOVATION FUND OF GUANGDONG MEDICAL UNI	1	0.1
SCIENCE AND TECHNOLOGY KEY PROJECT FOUNDATION OF ZHANJIANG CITY	1	0.1
SCIENCE AND TECHNOLOGY KEY PROJECT OF FUJIAN PROVINCE CHINA	1	0.1
SCIENCE AND TECHNOLOGY OF SHANXI AGRICULTURAL UNIVERSITY SHANXI	1	0.1
SCIENCE AND TECHNOLOGY PLAN PROJECT OF GUANGDONG PROVINCE	1	0.1
SCIENCE AND TECHNOLOGY PLAN PROJECTS OF GUANGDONG PROVINCE	1	0.1
SCIENCE AND TECHNOLOGY PLANNING PROJECT OF DONGGUAN CITY	1	0.1
SCIENCE AND TECHNOLOGY PLANNING PROJECT OF GUANGDONG PROVINCE C	1	0.1
SCIENCE AND TECHNOLOGY PLANNING PROJECT OF GUANGZHOU CITY GUANG	1	0.1
SCIENCE AND TECHNOLOGY PLANNING PROJECTS OF XIAMEN SCIENCE AND	1	0.1
SCIENCE AND TECHNOLOGY PROGRAM OF GUANGXI	1	0.1
SCIENCE AND TECHNOLOGY PROJECT	1	0.1
SCIENCE AND TECHNOLOGY PROJECT OF DONGGUAN	1	0.1
SCIENCE AND TECHNOLOGY PROJECT OF GUANGDONG PROVINCE	1	0.1
SCIENCE AND TECHNOLOGY PROJECT OF QIQIHAR SCIENCE AND TECHNOLOG	1	0.1
SCIENCE AND TECHNOLOGY RESEARCH PROJECT OF HEILONGJIANG PROVINC	1	0.1
SCIENCE AND TECHNOLOGY SERVICE NETWORK INITIATIVE OF CHINESE AC	1	0.1
SCIENCE AND TECHNOLOGY SUPPORT PROJECT OF SICHUAN PROVINCE	1	0.1
SCIENCE FOUNDATION FOR YOUTH SCHOLARS OF XIANGYA HOSPITAL CENTR	1	0.1
SCIENCE FOUNDATION OF FIRST HOSPITAL OF LANZHOU UNIVERSITY	1	0.1
SCIENCE FOUNDATION OF GUANGDONG MEDICAL UNIVERSITY	1	0.1
SCIENCE FOUNDATION OF GUANGDONG SECOND PROVINCIAL GENERAL HOSPI	1	0.1
SCIENCE FOUNDATION OF HEBEI PROVINCE	1	0.1
SCIENCE FOUNDATION OF QILU HOSPITAL OF SHANDONG UNIVERSITY	1	0.1
SCIENCE FOUNDATION OF SHANGHAI 9TH PEOPLE S HOSPITAL	1	0.1
SCIENCE FOUNDATION OF SHANGHAI EXCELLENT YOUTH SCHOLARS	1	0.1
SCIENCE FUND FOR CREATIVE RESEARCH GROUPS	1	0.1
SCIENCE FUNDING OF HEALTH DEPARTMENT SHAANXI PROVINCE	1	0.1
SCIENCE TECHNOLOGY FOUNDATION OF SICHUAN PROVINCE CHINA	1	0.1
SCIENCE TECHNOLOGY PROJECT OF SUZHOU XIANGCHENG DISTRICT	1	0.1
SCIENTIFIC AND TECHNOLOGICAL BUREAU OF SHAANXI	1	0.1
SCIENTIFIC AND TECHNOLOGICAL PROJECTS OF SHAANXI PROVINCE	1	0.1
SCIENTIFIC BENEFIT FOR PEOPLE PROJECT OF NINGBO	1	0.1

Table S4: Continued

Funding agency	Record count	% of 998
SCIENTIFIC INNOVATION TEAM PROJECT OF NINGBO CITY	1	0.1
SCIENTIFIC PROGRAM OF JINTAN DISTRICT	1	0.1
SCIENTIFIC RESEARCH FOUNDATION FOR INTRODUCED TALENTS CANCER HO	1	0.1
SCIENTIFIC RESEARCH FOUNDATION FOR JIANGSU PROVINCIAL COMMISSIO	1	0.1
SCIENTIFIC RESEARCH FOUNDATION OF GRADUATE SCHOOL OF NINGBO UNI	1	0.1
SCIENTIFIC RESEARCH FOUNDATION OF THE GRADUATE SCHOOL OF SOUTHE	1	0.1
SCIENTIFIC RESEARCH PROJECT OF THE GUANGZHOU EDUCATION BUREAU	1	0.1
SCIENTIFIC RESEARCH PROJECT OF THE HEALTH PLANNING COMMITTEE OF	1	0.1
SCIENTIFIC RESEARCH PROJECTS OF HEALTH AND FAMILY PLANNING COMM	1	0.1
SCIENTIFIC RESEARCH START UP FUNDING FOR AD VANCED TALENTS	1	0.1
SCIENTIFIC RESEARCH START UP FUNDING FOR ADVANCED TALENTS	1	0.1
SCIENTIST DEVELOPMENT GRANT	1	0.1
SEED GRANTS FROM THE PKU SCHOOL OF STOMATOLOGY FOR POSTDOC BEIJ	1	0.1
SEJER PERSSON AND LIS KLUVER PERSSONS FOUNDATION	1	0.1
SENATE OF BERLIN GERMANY	1	0.1
SENRI LIFE SCIENCE FOUNDATION	1	0.1
SEU NMU JOINT PROJECT	1	0.1
SHAANXI POSTDOCTORAL SCIENCE FOUNDATION	1	0.1
SHAANXI PROVINCIAL DEPARTMENT OF EDUCATION	1	0.1
SHAANXI SCIENCE AND TECHNOLOGY PROJECT	1	0.1
SHANDONG PROVINCE	1	0.1
SHANDONG PROVINCE KEY RD PLAN	1	0.1
SHANDONG PROVINCE NATURAL SCIENCE OUTSTANDING YOUTH FUND	1	0.1
SHANDONG PROVINCIAL HOSPITAL	1	0.1
SHANDONG SCIENCE AND TECHNOLOGY DEVELOPMENT PROGRAM	1	0.1
SHANGHAI 111 PROJECT	1	0.1
SHANGHAI HEALTH SYSTEM OUTSTANDING YOUNG TALENT TRAINING PLAN	1	0.1
SHANGHAI JIAO TONG UNIVERSITY SCHOOL OF MEDICINE DOCTORAL INNOV	1	0.1
SHANGHAI KEY DISCIPLINES	1	0.1
SHANGHAI KEY LABORATORY OF FEMALE REPRODUCTIVE ENDOCRINE RELATE	1	0.1
SHANGHAI LEADING TALENT PROJECTS	1	0.1
SHANGHAI MUNICIPAL COMMISSION OF HEALTH AND FAMILY PLANNING KEY	1	0.1
SHANGHAI MUNICIPAL EDUCATION COMMISSION SHMEC	1	0.1
SHANGHAI MUNICIPAL HEALTH BUREAU	1	0.1
SHANGHAI MUNICIPAL PLANNING COMMISSION OF SCIENCE AND RESEARCH	1	0.1
SHANGHAI RISING STAR PROGRAM A TYPE	1	0.1
SHANGHAI SCIENCE AND TECHNOLOGY COMMISSION MEDICAL GUIDE PROJEC	1	0.1
SHANGHAI SCIENCE AND TECHNOLOGY COMMISSION WESTERN MEDICINE GUI	1	0.1
SHANXI INTERNATIONAL SCI TECHNOLOGICAL COLLABORATION GRANT	1	0.1

Table S4: Continued

Funding agency	Record count	% of 998
SHENGJING FREE RESEARCHER PROJECT FOUNDATION	1	0.1
SHENYANG SCIENCE AND TECHNOLOGY PLAN PROJECTS	1	0.1
SHENZHEN MUNICIPAL GOVERNMENT OF CHINA	1	0.1
SHENZHEN SCIENCE AND TECHNOLOGY PROJECTOR OF GUANGDONG PROVINCE	1	0.1
SHENZHEN SCIENCE TECHNOLOGY AND INNOVATION COMMISSION	1	0.1
SHENZHEN SCIENTIFIC INNOVATION COMMITTEE FUND	1	0.1
SHOUGUANG PEOPLE S HOSPITAL	1	0.1
SICHUAN MEDICAL LAW RESEARCH CENTER	1	0.1
SICHUAN PROVINCE SCIENCE AND TECHNOLOGY SUPPORT PLAN	1	0.1
SINF GRANT	1	0.1
SIROCCO EU CONSORTIUM	1	0.1
SIX MAJOR TALENT SUMMIT OF JIANGSU PROVINCE	1	0.1
SIX TALENT PEAKS PROGRAM OF JIANGSU PROVINCE	1	0.1
SIX TALENT PEAKS PROJECT OF JIANGSU PROVINCE	1	0.1
SOCIAL DEVELOPMENT FOUNDATION OF JIANGSU PROVINCE CHINA	1	0.1
SOCIAL DEVELOPMENT FOUNDATION OF NANTONG CITY	1	0.1
SOCIAL DEVELOPMENT GUIDANCE PROJECT OF ZHENJIANG	1	0.1
SOCIAL DEVELOPMENT PROJECT OF TAIZHOU CITY CHINA	1	0.1
SOCIAL DEVELOPMENT PROJECTS OF JIANGSU PROVINCE	1	0.1
SOCIAL SCIENCE AND TECHNOLOGY DEVELOPMENT PROJECTS OF DONGGUAN	1	0.1
SOCIETY IN SCIENCE BRANCO WEISS FELLOWSHIP	1	0.1
SOOCHOW SCHOLAR PROJECT OF SOOCHOW UNIVERSITY	1	0.1
SOUTH AUSTRALIAN GOVERNMENT DEPARTMENT OF STATE DEVELOPMENT	1	0.1
SOUTH CAROLINA SEA GRANT CONSORTIUM	1	0.1
SOUTH CHINA AGRICULTURAL UNIVERSITY	1	0.1
SOUTHEAST ASIA BIODIVERSITY RESEARCH INSTITUTE CHINESE ACADEMY	1	0.1
SOUTHERN MEDICAL UNIVERSITY CLINICAL RESEARCH PROJECT	1	0.1
SOUTHWEST HOSPITAL RESEARCH PROJECT	1	0.1
SPANISH MINISTERIO DE CIENCIA INNOVACION Y UNIVERSIDADES	1	0.1
SPANISH MINISTERIO DE ECONOMIA Y COMPETITIVIDAD	1	0.1
SPANISH NETWORK OF MULTIPLE SCLEROSIS REGIONAL COUNCIL OF GIPUZ	1	0.1
SPECIAL CLINICAL MEDICINE RESEARCH OF CHINESE MEDICAL ASSOCIATI	1	0.1
SPECIAL FOUNDATION FOR SCIENCE AND TECHNOLOGY PROGRAM IN LIAONI	1	0.1
SPECIAL FUNDS FOR PUBLIC WELFARE RESEARCH AND CAPACITY BUILDING	1	0.1
SPECIAL INNOVATIVE PROVINCE CONSTRUCTION IN ANHUI PROVINCE	1	0.1
SPECIAL PROGRAM FOR APPLIED RESEARCH ON SUPER COMPUTATION OF TH	1	0.1
SPECIAL PROGRAM FOR SINO RUSSIAN JOINT RESEARCH MINISTRY OF SCI	1	0.1
SPECIAL PROGRAM OF TALENTS DEVELOPMENT FOR EXCELLENT YOUTH SCHO	1	0.1

Table S4: Continued

Funding agency	Record count	% of 998
SPECIFIC PROJECTS IN SCIENCE AND TECHNOLOGY OF HENAN PROVINCE	1	0.1
ST JOHNS RIVER ECONOMIC IMPACT STUDY PHASE 1 ECOSYSTEM SERVICES	1	0.1
STANFORD CENTER FOR COMPUTATIONAL EVOLUTIONARY AND HUMAN GENOMI	1	0.1
STANFORD UNIVERSITY	1	0.1
STATE KEY BASIC RESEARCH PROGRAM PROJECT	1	0.1
STATE KEY LABORATORY FOR BIOLOGY OF PLANT DISEASES AND INSECT P	1	0.1
STATE KEY LABORATORY OF CANCER BIOLOGY FOURTH MILITARY MEDICAL	1	0.1
STATE KEY LABORATORY OF NATURAL AND BIOMIMETIC DRUGS	1	0.1
STATE KEY LABORATORY OF ONCOGENES AND RELATED GENES	1	0.1
STATE KEY LABORATORY PROGRAM	1	0.1
STATE PROJECT FOR ESSENTIAL DRUG RESEARCH AND DEVELOPMENT	1	0.1
STICHTING SPIEREN VOOR SPIEREN	1	0.1
STRATEGIC PRIORITY RESEARCH PROGRAM OF CHINESE ACADEMY OF SCIEN	1	0.1
STRATEGIC PRIORITY RESEARCH PROGRAMS OF THE CHINESE ACADEMY OF	1	0.1
STRATEGIC RESEARCH	1	0.1
STUDENT S PLATFORM FOR INNOVATION AND ENTREPRENEURSHIP TRAINING	1	0.1
STUDIENSTIFTUNG DES DEUTSCHEN VOLKES	1	0.1
SUBEI SCIENCE AND TECHNOLOGY PROGRAM OF JIANGSU PROVINCE OF CHI	1	0.1
SUN YAT SEN UNIVERSITY STARTING FUNDS FOR YOUNG TEACHERS	1	0.1
SUPPORTING PROJECT FOR CLIMBING SCHOLARS IN LIAONING PROVINCIAL	1	0.1
UPR MDACC PARTNERSHIP FOR EXCELLENCE IN CANCER RESEARCH PILOT P	1	0.1
UST UCSD INTERNATIONAL CENTER OF EXCELLENCE IN ADVANCED BIOENGI	1	0.1
UTHEALTH INNOVATION FOR CANCER PREVENTION RESEARCH TRAINING PRO	1	0.1
VETERANS GENERAL HOSPITALS AND UNIVERSITY SYSTEM OF TAIWAN VGHU	1	0.1
VIRAL HEPATITIS AND RELATED LIVER DISEASE RESEARCH INNOVATION T	1	0.1
VOLKSWAGEN	1	0.1
WECHSLER FUND	1	0.1
WEIHAN YU YOUTH SCIENCE FUND PROJECT OF HARBIN MEDICAL UNIVERSI	1	0.1
WENLING CITY SCIENCE AND TECHNOLOGY PROJECT	1	0.1
WENZHOU MEDICAL UNIVERSITY	1	0.1
WENZHOU MUNICIPAL SCIENCE AND TECHNOLOGY BUREAU	1	0.1
WENZHOU SCIENCE AND TECHNOLOGY BUREAU OF CHINA	1	0.1
WENZHOU SCIENCE AND TECHNOLOGY BUREAU PROGRAM	1	0.1
WILD FUND AWARDS	1	0.1
WU JIEPING MEDICAL FOUNDATION SPECIAL RESEARCH FUND FOR CLINICA	1	0.1
WUXI HEALTH AND FAMILY PLANNING COMMISSION	1	0.1
WUXI MEDICAL INNOVATION TEAM	1	0.1
XIAMEN UNIVERSITY	1	0.1
XINMIAO TALENTS PROGRAM OF ZHEJIANG PROVINCE	1	0.1

Table S4: Continued

**Funding agency**	**Record count**	**% of 998**
XUZHOU SCIENCE AND TECHNOLOGY PROJECT	1	0.1
YANGFAN TRAINING PROGRAM OF GUANGDONG PROVINCE	1	0.1
YANGZHOU CITY SCIENCE AND TECHNOLOGY PLAN SOCIAL DEVELOPMENT PR	1	0.1
YANGZHOU UNIVERSITY OF CHINA	1	0.1
YOUNG AND MIDDLE AGED ACADEMIC LEADER OF JIANGSU COLLEGE AND UN	1	0.1
YOUNG AND MIDDLE AGED SCIENCE AND TECHNOLOGY INNOVATION TALENT	1	0.1
YOUNG SCIENTISTS PROGRAM FROM EYE AND ENT HOSPITAL	1	0.1
YOUNG SCIENTISTS PROGRAM FROM EYE ENT HOSPITAL	1	0.1
YOUNG TEACHERS CULTIVATING PROJECT OF SUN YAT SEN UNIVERSITY	1	0.1
YOUTH 1000 TALENT PLAN	1	0.1
YOUTH FOUNDATION OF EDUCATIONAL COMMITTEE OF HEBEI PROVINCE	1	0.1
YOUTH FOUNDATION OF SHANXI PROVINCE	1	0.1
YOUTH FUND OF FUJIAN HEALTH DEPARTMENT	1	0.1
YOUTH INNOVATION FUND PROJECT OF THE FIRST AFFILIATED HOSPITAL	1	0.1
YOUTH SCIENCE FOUNDATION OF HEILONGJIANG PROVINCE	1	0.1
YOUTH SCIENTIFIC FUNDS YOUTH FUND PROJECT	1	0.1
YOUTH TALENT SUPPORT PROGRAM OF HARBIN MEDICAL UNIVERSITY CANCE	1	0.1
YUNNAN HEALTH TRAINING PROJECT OF HIGH LEVEL TALENTS	1	0.1
YUNNAN PROVINCE MAJOR SCIENCE AND TECHNOLOGY PROJECT	1	0.1
YUNNAN PROVINCIAL DEPARTMENT OF SCIENCE AND TECHNOLOGY KUNMING	1	0.1
YUNNAN PROVINCIAL HEALTH AND FAMILY PLANNING COMMISSION MEDICAL	1	0.1
YUNNAN PROVINCIAL SCIENCE AND TECHNOLOGY DEPARTMENT	1	0.1
YUNNAN SCIENCE AND TECHNOLOGY TALENT AND PLATFORM PROGRAM	1	0.1
ZHEJIANG MEDICAL AND HEALTH PROJECT	1	0.1
ZHEJIANG MEDICAL ASSOCIATION CLINICAL RESEARCH FUND	1	0.1
ZHEJIANG PROVINCE NATURE SCIENCE RESEARCH FUNDING	1	0.1
ZHEJIANG PROVINCE SOCIAL DEVELOPMENT RESEARCH PROJECT	1	0.1
ZHEJIANG PROVINCIAL EDUCATION SCIENTIFIC RESEARCH ITEMS	1	0.1
ZHEJIANG PROVINCIAL MEDICAL AND HEALTH SCIENCE AND TECHNOLOGY P	1	0.1
ZHEJIANG PROVINCIAL TRADITIONAL CHINESE MEDICINE FOUNDATION OF	1	0.1
ZHEJIANG UNIVERSITY	1	0.1
ZOLL	1	0.1
ZOLL MEDICAL	1	0.1
ZOLL MEDICAL CORPORATION	1	0.1

Table S5: The citation frequency per paper of all countries/regions that published research related to circular RNA

Rank	Country	Documents	Citations	Citations per paper
1	argentina	1	385	385.00
2	israel	9	1615	179.44
3	germany	45	4798	106.62
4	singapore	5	446	89.20
5	austria	6	494	82.33
6	sweden	6	472	78.67
7	ireland	1	59	59.00
8	norway	4	234	58.50
9	denmark	23	1344	58.43
10	kuwait	1	56	56.00
11	scotland	1	56	56.00
12	qatar	1	52	52.00
13	australia	20	944	47.20
14	usa	181	8460	46.74
15	italy	13	575	44.23
16	mexico	3	128	42.67
17	taiwan	10	407	40.70
18	netherlands	12	449	37.42
19	canada	21	774	36.86
20	japan	14	442	31.57
21	luxembourg	1	28	28.00
22	england	13	340	26.15
23	brazil	6	127	21.17
24	france	12	250	20.83
25	peoples r china	729	13333	18.29
26	chile	2	33	16.50
27	saudi arabia	2	33	16.50
28	slovenia	1	13	13.00
29	pakistan	3	37	12.33

Table S5: Continued

Rank	Country	Documents	Citations	Citations per paper
30	switzerland	2	22	11.00
31	botswana	1	11	11.00
32	zimbabwe	2	21	10.50
33	indonesia	1	10	10.00
34	romania	1	10	10.00
35	russia	9	68	7.56
36	malaysia	7	52	7.43
37	india	11	80	7.27
38	spain	12	65	5.42
39	south korea		20	5.00
40	south africa		15	5.00
41	croatia			2.00
42	iran			1.00
43	belgium			0.00
44	kenya			0.00
45	poland			0.00
46	turkey			0.00

References

1. Glazar P, Papavasileiou P, Rajewsky N. circBase: a database for circular RNAs. RNA. 2014; 20(11): 1666-1670.
2. Dong R, Ma XK, Li GW, Yang L. CIRCpedia v2: An updated database for comprehensive circular RNA annotation and expression comparison. Genomics, Proteomics & Bioinformatics. 2018; 16(4): 226-233.
3. Dudekula DB, Panda AC, Grammatikakis I, De S, Abdelmohsen K, Gorospe M. CircInteractome: a web tool for exploring circular RNAs and their interacting proteins and microRNAs. RNA Biol. 2016; 13(1): 34-42.
4. Li JH, Liu S, Zhou H, Qu LH, Yang JH. starBase v2.0: decoding miRNA-ceRNA, miRNA-ncRNA and protein-RNA interaction networks from large-scale CLIP-Seq data. Nucleic Acids Res. 2014; 42(Database issue): D92-D97.
5. Ghosal S, Das S, Sen R, Basak P, Chakrabarti J. Circ2Traits: a comprehensive database for circular RNA potentially associated with disease and traits. Front Genet. 2013; 4: 283.
6. Xia S, Feng J, Chen K, Ma Y, Gong J, Cai F, et al. CSCD: a database for cancer-specific circular RNAs. Nucleic Acids Res. 2018; 46(D1): D925-D929.
7. Xia S, Feng J, Lei L, Hu J, Xia L, Wang J, et al. Comprehensive characterization of tissue-specific circular RNAs in the human and mouse genomes. Brief Bioinform. 2017; 18(6): 984-992.
8. Liu YC, Li JR, Sun CH, Andrews E, Chao RF, Lin FM, et al. CircNet: a database of circular RNAs derived from transcriptome sequencing data. Nucleic Acids Res. 2016; 44(D1): D209-D215.
9. Zheng LL, Li JH, Wu J, Sun WJ, Liu S, Wang ZL, et al. deepBase v2.0: identification, expression, evolution and function of small RNAs, LncRNAs and circular RNAs from deep-sequencing data. Nucleic Acids Res. 2016; 44(D1): D196-D202.
10. Chen X, Han P, Zhou T, Guo X, Song X, Li Y. circRNADb: a comprehensive database for human circular RNAs with protein-coding annotations. Sci Rep. 2016; 6: 34985.
11. Vo JN, Cieslik M, Zhang Y, Shukla S, Xiao L, Zhang Y, et al. The landscape of Circular RNA in cancer. Cell. 2019; 176(4): 869-881. e13.
12. Li S, Li Y, Chen B, Zhao J, Yu S, Tang Y, et al. exoRBase: a database of circRNA, lncRNA and mRNA in human blood exosomes. Nucleic Acids Res. 2018; 46(D1): D106-D112.
13. Zhao Z, Wang K, Wu F, Wang W, Zhang K, Hu H, et al. circRNA disease: a manually curated database of experimentally supported circRNA-disease associations. Cell Death Dis. 2018; 9(5): 475.
14. Yao D, Zhang L, Zheng M, Sun X, Lu Y, Liu P. Circ2Disease: a manually curated database of experimentally validated circRNAs in human disease. Sci Rep. 2018; 8(1): 11018.
15. Chu Q, Zhang X, Zhu X, Liu C, Mao L, Ye C, et al. PlantcircBase: a database for plant circular RNAs. Mol Plant. 2017; 10(8): 1126-1128.
16. Zhang P, Meng X, Chen H, Liu Y, Xue J, Zhou Y, et al. PlantCircNet: a database for plant circRNA-miRNA-mRNA regulatory networks. Database (Oxford). 2017; 2017.
